# Development and validation of a whole-exome sequencing test for simultaneous detection of point mutations, indels and copy-number alterations for precision cancer care

**DOI:** 10.1038/npjgenmed.2016.19

**Published:** 2016-07-20

**Authors:** Hanna Rennert, Kenneth Eng, Tuo Zhang, Adrian Tan, Jenny Xiang, Alessandro Romanel, Robert Kim, Wayne Tam, Yen-Chun Liu, Bhavneet Bhinder, Joanna Cyrta, Himisha Beltran, Brian Robinson, Juan Miguel Mosquera, Helen Fernandes, Francesca Demichelis, Andrea Sboner, Michael Kluk, Mark A Rubin, Olivier Elemento

**Affiliations:** 1 Caryl and Israel Englander Institute for Precision Medicine, New York Presbyterian Hospital-Weill Cornell Medicine, New York, NY, USA; 2 Department of Pathology and Laboratory Medicine, Weill Cornell Medicine, New York, NY, USA; 3 Institute for Computational Biomedicine, Weill Cornell Medicine, New York, NY, USA; 4 Genomics Core Facility, Weill Cornell Medicine, New York, NY, USA; 5 Centre for Integrative Biology (CIBIO), University of Trento, Trento, Italy; 6 Department of Medicine, Division of Hematology and Medical Oncology, Weill Cornell Medicine, New York, NY, USA

## Abstract

We describe Exome Cancer Test v1.0 (EXaCT-1), the first New York State-Department of Health-approved whole-exome sequencing (WES)-based test for precision cancer care. EXaCT-1 uses HaloPlex (Agilent) target enrichment followed by next-generation sequencing (Illumina) of tumour and matched constitutional control DNA. We present a detailed clinical development and validation pipeline suitable for simultaneous detection of somatic point/indel mutations and copy-number alterations (CNAs). A computational framework for data analysis, reporting and sign-out is also presented. For the validation, we tested EXaCT-1 on 57 tumours covering five distinct clinically relevant mutations. Results demonstrated elevated and uniform coverage compatible with clinical testing as well as complete concordance in variant quality metrics between formalin-fixed paraffin embedded and fresh-frozen tumours. Extensive sensitivity studies identified limits of detection threshold for point/indel mutations and CNAs. Prospective analysis of 337 cancer cases revealed mutations in clinically relevant genes in 82% of tumours, demonstrating that EXaCT-1 is an accurate and sensitive method for identifying actionable mutations, with reasonable costs and time, greatly expanding its utility for advanced cancer care.

## Introduction

Identification of genetic alterations by next-generation sequencing (NGS) has become the standard of care in genomic medicine.^1^ Currently, numerous NGS assays and platforms are commercially available, using either targeted sequencing or whole-genome/-exome sequencing (WES/WGS). WES applies NGS technology to identify genetic variants in the coding regions (exons) of genes, encompassing the majority of disease-causing mutations.^2^ WES has proved useful for constitutional disorders with phenotypic and genotypic heterogeneity, or patients with rare disorders in whom differential diagnostic tests may be lengthy and costly,^3,4^ enabling wide-spectrum screening and easier diagnosis.^1^

Although WES is widely used in clinical genetics, it is only recently starting to gain adoption for cancer care. Currently, NGS-based oncology DNA testing is still primarily performed using ‘hotspot’ or targeted panels of known cancer-associated genes with relevance across a confined scope of cancers, such as solid or haematological neoplasms. Such limited testing is aimed at selecting therapeutics for certain genetic changes.^5^ In this case, high-sequencing depth is achieved at relatively low costs and fast turnaround, but the capacity for detecting recently characterised, novel cancer-associated variants or structural variants is limited.^5,6^ Using WES, it is currently feasible to not only detect a rapidly growing set of known clinically relevant mutations but also identify novel or unexpected important variations, including constitutional mutations in cancer predisposing genes, pharmacogenomics variants impacting therapy and discovery of MHC neoepitope peptides among passenger mutations that may prove informative as biomarkers of immunotherapy response.^7,8^

Thus far, the use of WES in cancer has largely taken place in the setting of large research studies such as The Cancer Genome Atlas (TCGA), Stand Up To Cancer (SU2C)^9^ as well as other non-clinical discovery studies. Integration of WES into precision cancer care has lagged behind primarily owing to challenges that have not been rigorously addressed and validated in the research setting. For example, challenging samples such as small and poor quality from formalin-fixed (FFPE) tissues and low tumour purity samples must routinely be analysed in the context of a clinical grade assay. In research projects such as TCGA, inclusion of such samples can more easily be avoided. Another challenge is that library preparation techniques and computational approaches required to detect the wide-spectrum of mutations and genes queried by WES require a comprehensive validation procedure demonstrating the ability of the test to identify actionable mutations at an acceptable analytical sensitivity. Finally, for many providers and insurance companies WES and WGS are still considered investigational for all indications.

Although these challenges are recognised and despite a wide range of assays and platforms available, WES has not yet been validated for the clinical laboratory and has not been fully characterised in the literature with regards to the analytic validity of the testing and the various types of relevant mutations. The few existing professional guidelines given by the American College of Medical Genetics,^10^ the College of American Pathologist and New York State-Department of Health (NYS-DOH)^11^ give only high-level directions for implementing NGS testing and are geared, primarily towards the use of targeted panels rather than WES. By all accounts, NYS-DOH requirements are among the most rigorous guidelines yet published and are likely to serve as a paradigm for suggested future type of guidelines that might be required by the Food and Drug Administration (FDA).^12^ These efforts are also in line with the new precision medicine initiative announced by the U.S. President Obama and Vice-President Biden’s Moonshot initiative with the intent to bring us closer to curing cancer and give all of us access to a more personalised and genomic-driven medicine.^13^

Here we describe the development and analytical characteristics of NYS-DOH approved clinical Exome Cancer Test Version 1 (EXaCT-1) suitable for simultaneous detection of somatic SNV, indels and CNAs. Our test uses the Agilent HaloPlex capture platform (Agilent Technologies, Santa Clara, CA, USA) and the Illumina HiSeq 2500 system for sequencing. To validate the test, we initially focused on actionable mutations in five principal genes and according to NYS-DOH guidelines. We describe and discuss an automated computational framework for data analysis, variant interpretation and reporting. Our results clearly demonstrate feasibility of integration of WES into precision cancer care.

## Results

### EXaCT-1 overview

**Figure 1:**
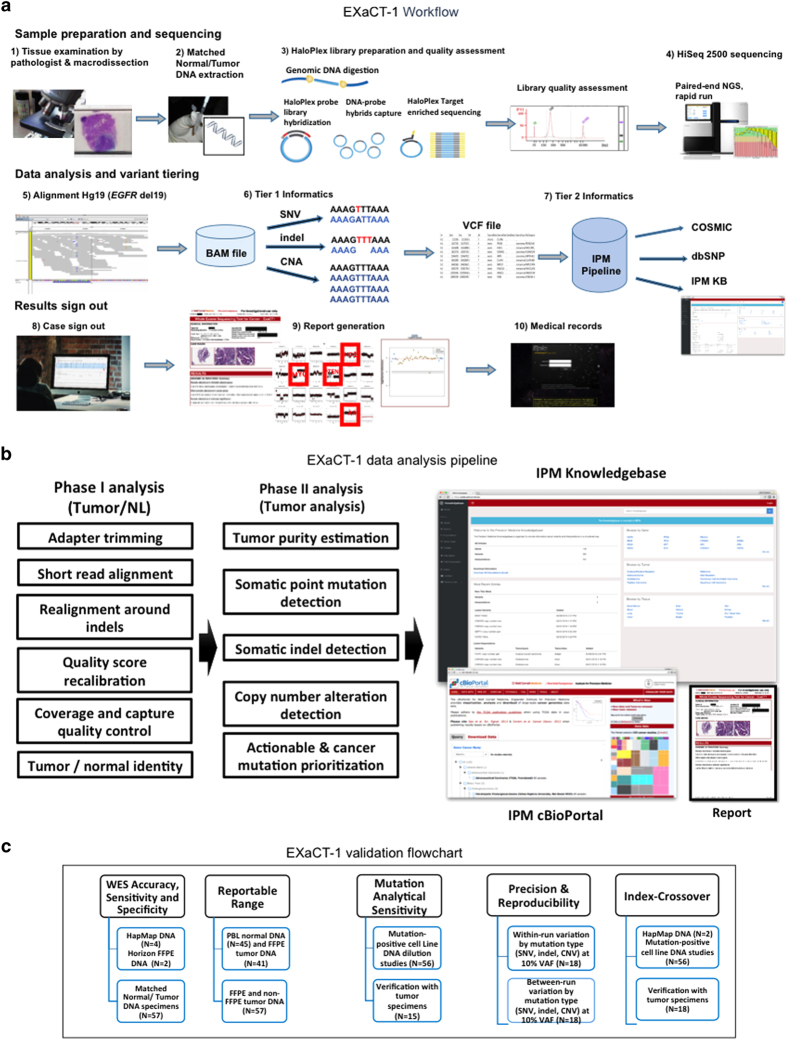
Schematic view of EXaCT-1 assay workflow. (**a**) (1) Slides are assessed by pathologist for neoplastic content, and tumour tissue marked and macrodissected. (2) DNA from fresh or FFPE tumour tissue and matched normal control specimen is extracted. (3) DNA is then enriched for exome sequences (357,999 exons corresponding to 21,522 genes) with HaloPlex technology described in this protocol in four major steps: fragmentation by restriction enzyme digestion, hybridisation to HaloPlex probes and introduction of Illumina sequence motifs, solid-phase capture and DNA ligation, and amplification of targeted fragments by PCR, followed by (4) sequencing on an Illumina HiSeq 2500 rapid mode, four samples per lane. (5) Paired-end reads are aligned to the human genome and (6) variant calls are made. (7) Variants are annotated and classified by our internally developed informatics pipeline, using publicly available and our own developed knowledge base. (8) The case is reviewed and results interpreted by a molecular pathologist who also signs it out in the LIS. (9) A report is then automatically generated and (10) dispensed to medical records. EXaCT-1 data analysis pipeline. (**b**) Schematic view of EXaCT-1 assay validation. (**c**) DNA derived from matched normal/tumour pairs from either fresh or FFPE specimens as well as standardised commercial DNA material were used to demonstrate the accuracy, sensitivity, specificity, reproducibility and precision of the assay using bioinformatics process for the detection of numerous types of variants (SNV, indel and CNV gain), according to NYS-DOH NGS guidelines for somatic genetic variant detection. IPM, Institute for Precision Medicine.

We have developed a comprehensive NGS-based workflow for mutation analysis in cancer, comprising sample processing, sequencing and computational framework for data analysis and reporting (Figure 1a,b). The test uses the HaloPlex Target Enrichment System (Agilent Technologies) for target amplification ((357,999 exons/21,522 genes), followed by sequencing on the Illumina system. Schematics of EXaCT-1 workflow is depicted in Figure 1. Briefly, hematoxylin-and-eosin-stained slides of both FFPE and frozen tumour tissue blocks were reviewed by the pathologists for neoplastic content, and high-density tumour areas were selected for manual macrodissection and DNA extraction. DNA was then extracted from tumours and paired normal tissue, fragmented by restriction enzymes and amplified by PCR following probe-hybridisation and -circularisation of the biotinylated DNA-probe hybrids by ligation. PCR products were then subjected to onboard cluster generation and 100-bp-paired-end sequencing on an Illumina HiSeq 2500 in rapid mode, four samples per lane (150 Mb per lane). Sequencing data are then analysed by our internally developed informatics pipeline. In the first step of the analysis paired-end reads are aligned to the human genome (reference GRC37/hg19) for each pair of tumour-normal specimens and quality metrics are generated. In the second step, tumour purity is assessed by computational methods and gene variants are called and annotated. Mutations are then classified into three tiers based on their clinical relevance and actionability, using publicly available and our own developed knowledge base. Mutation results are then reviewed and interpreted in the context of the clinical and pathological information by a board-certified molecular pathologist, who also releases the results (sign-out) in the laboratory information management system. The turnaround time for the entire assay from specimen acquisition to clinical reporting ranged from 2 to 5 weeks, with an average of 3 weeks.

### Establishment of quality metrics and reportable range

**Figure 2:**
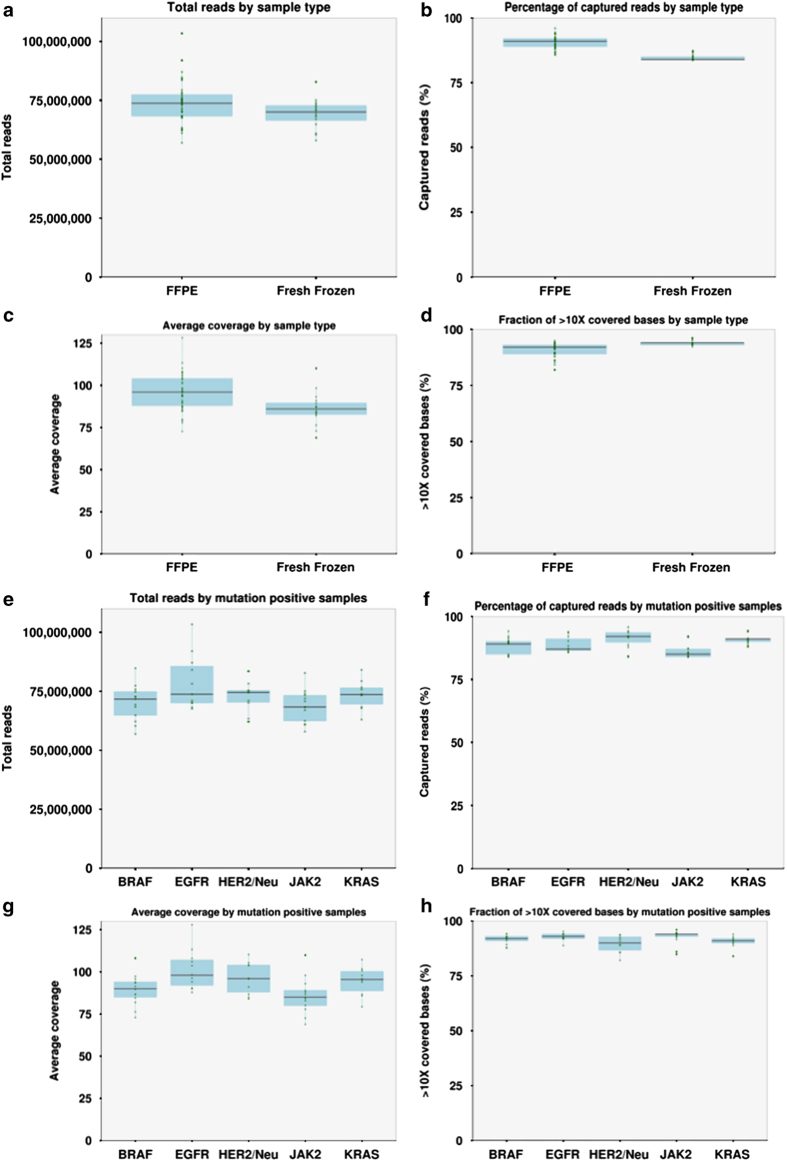
EXaCT-1 sequencing quality metrics. The EXaCT-1 was validated using 57 archival samples with known mutations comprising a diverse representation of solid tumours and haematological cancers. Total reads, percentage of captured reads, average coverage and fraction of >10× covered bases by sample type (**a**–**d**) and mutation-positive sample (**e**–**h**) in FFPE (*n*=41) and fresh (*n*=16) tumour specimens. Additional QC metrics for all cases are available in Supplementary Tables 2 and 3. No statistically significant difference between FFPE and non-FFPE tissue and mutation-harbouring sample was observed in these analyses.

The quality, depth-of-coverage and accuracy metrics of the new HaloPlex-WES assay were first established using HapMap DNA specimens (NA12878/NA19240) and 57 matched normal/tumour samples positive for any of mutation in five genes (*KRAS*, *BRAF*, *JAK2*, *EGFR* and *HER2/neu*), according to NYS-DOH NGS guidelines for somatic genetic variant detection (Figure 1c). The average total number of reads and capture rates were similar: 70.4M vs 72.9M and 86% vs. 91% for the HapMap DNA and clinical specimens, respectively (Supplementary Tables 1 and 2). Similar high quality average coverage of 86× and 93× was obtained for the HapMap and clinical specimens, respectively, with at least 92–94% of the exome covered at ⩾10×. FFPE tissue DNA quality is expected to be highly variable owing to strand breakage and cross-linking by formalin fixation. Assessment of FFPE DNA quality by GAPDH-PCR demonstrated that approximately half of the clinical samples were low quality, requiring increased DNA input (Supplementary Table 3). Read and coverage statistics were similar for sequencing runs of libraries prepared from FFPE, fresh-frozen or blood/bone marrow specimens, indicating that FFPE tissues are suitable for NGS-based analyses (Supplementary Table 4). EXaCT-1 quality metrics (total reads, percentage of captured reads, average coverage, fraction of bases covered >10×, neoplastic content, variant allele frequency (VAF) and strand bias) did not differ significantly by sample type or mutation-positive sample either (Figure 2; Supplementary Figures 1 and 2).

To evaluate the accuracy, specificity and sensitivity of EXaCT-1 across the exome, germline sequence variation calls (homozygous and heterozygous) generated by our pipeline for NA12878 analysed in duplicate were compared with the publicly available whole-genome reference material.^14^ A total of 27.88 million bases in our assay had adequate calls available from the whole-genome reference material.^14^ This represents 76% (27.8 out of 36.7 million bases) of the HaloPlex exon capture regions. Of 27.88 million bases, 27.86 million were called homozygous reference sites, although 17,917 were designated as having non-reference variations in the benchmark data set. Of the latter, 773/17,917 (4.3%) variations were not detected by our pipeline (false negative). Conversely, there were only 156/27,859,024 (<0.001%) bases miscalled as non-reference (false positive) by EXaCT-1 compared with the reference benchmark (Table 1). This resulted in an estimated sensitivity, specificity and positive predictive value of 95.7%, 99.9% and 99.2%, respectively (Table 2). Further assessment of index cross-talk (defined as PCR contamination which can occur when preparing libraries from different DNA samples side by side) by reference material demonstrated a total error rate <0.3%, far below the limit of detection (LoD) of the assay (Supplementary Table 5).

**Figure 3:**
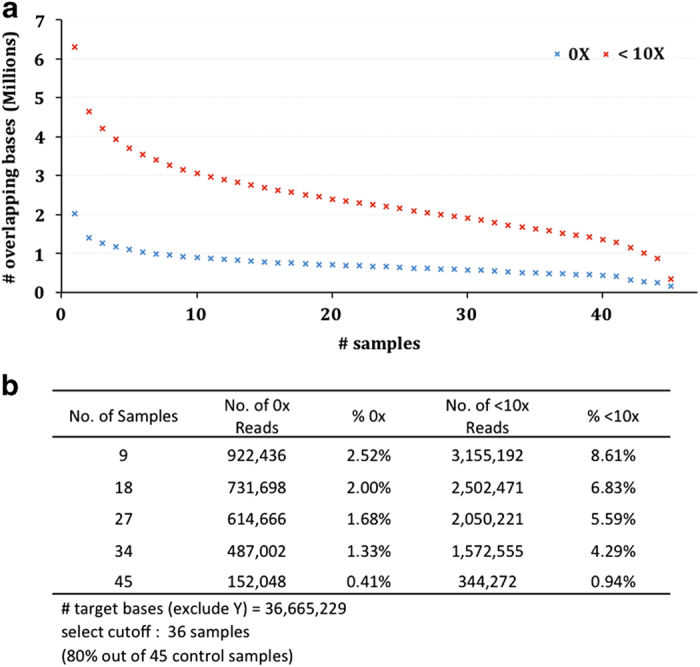
EXaCT-1 reportable range. Number and percentage of common low coverage bases shared by 36 (80%) and all 45 (100%) control (germline) samples obtained from normal tissue. (**a**) The germline samples were expected to have low genomic complexity and therefore provide more accurate assessment of coverage and performance statistics. Over 95% of the HaloPlex exome bases were covered at >10×. (**b**) These guaranteed regions that have constant higher than 10× coverage is our reportable range, which is listed on our website (https://rubinlab.med.cornell.edu/IPMWES/Haloplex_Exome_Sequencing_reportable_region.xlsx). This poorly covered 4.29% genomic area of the HaloPlex exome will be excluded from our reportable range. The reportable range is listed on https://rubinlab.med.cornell.edu/IPMWES/HaloPlex_low_coverage_region.xlsx.

The reportable range, defined by NYS-DOH guidelines as the genomic region in which sequence of an acceptable quality can be derived, was determined as the unique depth of coverage for each base position of the exome across a cohort of 45 constitutional DNA control samples. These samples were expected to have low genomic complexity and therefore provide more accurate assessment of coverage and performance statistics. Of the 37 million HaloPlex exome bases, only 4.29% bases had <10× coverage for at least 36/45 controls (80%), while <1% bases were covered at <10× across all 45 control samples (Figure 3). The poorly covered 4.29% genomic areas were excluded from our reportable range (http://trp.med.cornell.edu/IPMWES/HaloPlex_low_coverage_region.xlsx). Coverage and GC content in the target region for the 45 control samples were not correlated (Pearson coefficient=0.131), suggesting that GC content does not impact coverage significantly. No correlation with GC content was found for the 4.3% poorly covered (<10×) bases (Pearson coefficient=0.041) either.

**Figure 4:**
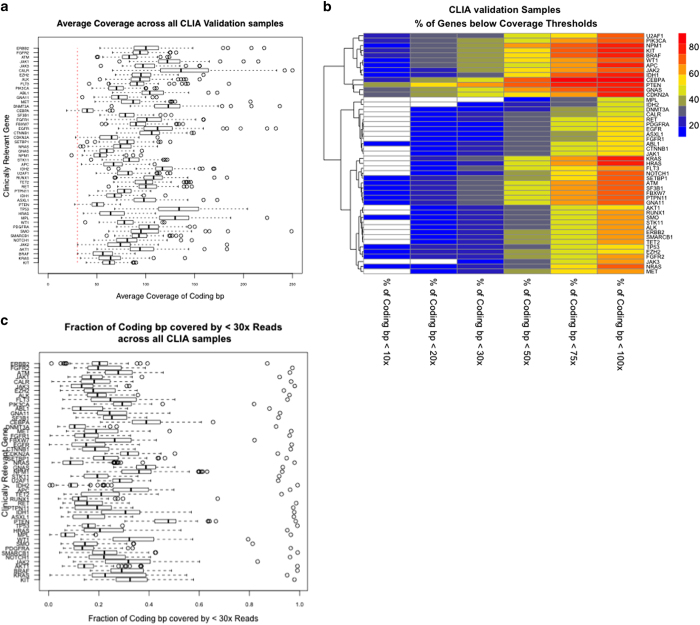
Coverage analysis for 49 Tier 1 clinically relevant genes. (**a**) The barplots show the mean bp coverage per gene across all validation samples (*n*=57). Each row is a gene and the barplot represents the distribution of the mean coverage across all specimens. This metric is measured by dividing the total coverage by the total number of coding bp. The dashed red line is the 30× threshold. (**b**) The heatmap shows percentage of coding bp (averaged across all samples of a group) of a gene (each row) below a specific coverage threshold (each column). The colours of the heatmap represent the percentage of the coding bp of a gene below a coverage threshold. The white colour shows that <10% of the coding bp of a gene are below a coverage threshold. For the majority of genes, only <10–20% of the coding regions are covered at <10×. (**c**) Fraction of coding bp covered by <30× reads across the validation sample cohort. On average, ~20% of the gene-coding regions were covered at <30×.

**Figure 5:**
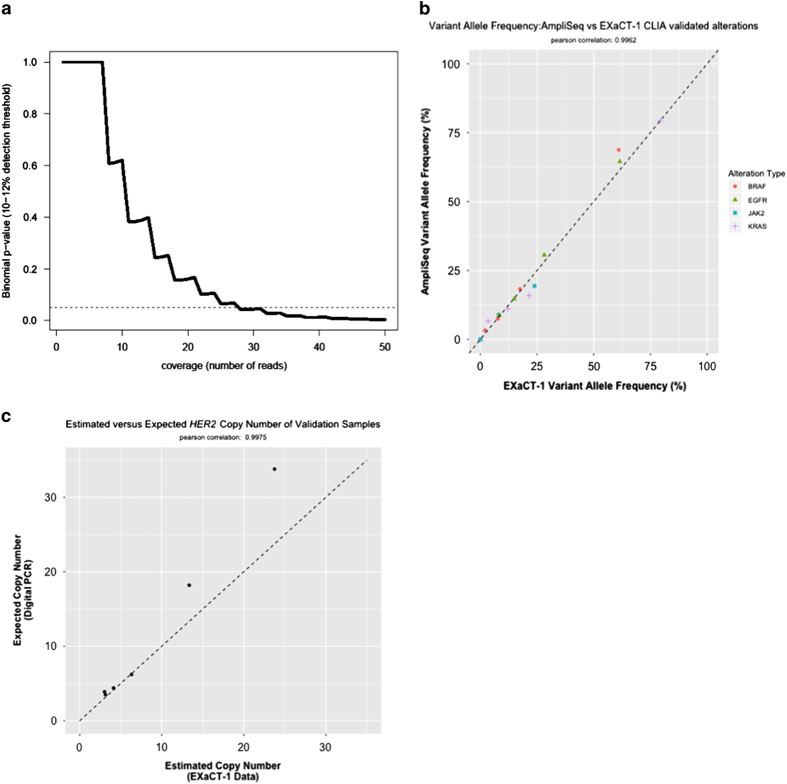
EXaCT-1 analytical sensitivity and power analysis. (**a**) Power analysis shows that at a VAF threshold of 10–12% VAF, at least 28× total coverage is needed (*P*⩽0.05) for avoiding false-negative results if no mutation is present (0 reads). (**b**) Analytical sensitivity for low VAF detection for selected mutations. Synthetic mixed samples were generated from mutation-positive cell lines diluted into HapMap DNA with mixed proportion as determined by Ion Torrent AmpliSeq Cancer Hot Spot assay (*EGFR*, *KRAS*, *BRAF* and *JAK2)* (**a**) and by digital droplet PCR (*HER2* copy number). (**c**) With proportions ranging between 50 and 2%. Experiments were performed in triplicate. The threshold of EXaCT-1 for detecting variants at low allele frequency was established at 10%.

To extend the assessment of coverage depth to clinically relevant loci, we also assessed unique coverage across 49 clinically relevant genes (Supplementary Table 6) in the clinical cohort (*N*=57 samples). A comprehensive literature review as well as discussion with a panel of oncologists and pathologists at our institution was used to select the 49 genes. These genes harbour mutations that are currently clinically actionable via FDA approved therapies, e.g., *HER2* amplification with Herceptin, *BRAF* mutations with vemurafenib, either by providing information about sensitivity or resistance to specific targeted therapies, or by providing prognostic information related to patient outcome. High mean coverage of at least 30× was present throughout the 49-gene capture region (Figure 4a). A heatmap of the corresponding data and analysis of the fraction bp covered by <30× reads showed that only 23% of captured coding bp, on average, did not achieve 30× unique coverage across the validation samples (Figure 4b,c). Power analysis to establish the minimum depth coverage required for accurate negative-mutation calls demonstrated that at a true VAF of 10% at least 28× coverage (*P*⩽0.05), is required for ensuring reliable negative call (Figure 5a). In other words, at less than 28×, there is a non-negligible chance that no mutated reads would be picked by chance even if VAF=10%. Exon-by-exon coverage for the five principal genes (*EGFR*, *KRAS*, *BRAF*, *JAK2 and HER2/Neu)* validated in this study demonstrated good mean coverage depth regardless of sample type, ranging from 58× (s.d.=8.0) for *KRAS* to 103X (s.d.=9.5) for *EGFR* exons (Supplementary Figures 3 and 4; Supplementary Table 7).

### Analytical sensitivity

The analytical sensitivity for detecting variants at low allele frequency (AF) assessed with undiluted DNA and serial dilutions of the mutation-positive DNA samples ranging between 2 to 50% were analysed to estimate the percentage of each mutation detected by the assay. Assay performance statistics of the cell lines employed for the analytical sensitivity studies by EXaCT-1 are presented in Supplementary Table 8. Review of the VAF unique to each cell line and synthetic dilution mix demonstrated good agreement between the observed and expected AFs as established by the AmpliSeq Cancer Hotspot assay (ThermoFisher Scientific, Waltham, MA, USA, Pearson correlation of 0.9962 and 0.9975 for the validated SNVs/indel and *HER2/neu* copy-number, respectively) with an overall LoD of 10% (Figure 5b, Supplementary Table 9). The LoD for *HER2/neu* in this validation yielded an average read count log2 ratio of 0.60, corresponding to 3.5 copies in this sample (on average and assuming a pure tumour sample) as assessed by droplet digital PCR (Figure 5c, Supplementary Table 9). Moreover, precision (within-run variation) and reproducibility (between-run variation) of EXaCT-1 assessed near the stated sensitivity of the assay (~10%) demonstrated 100% concordance in the mutation (SNV/indel) call rate across all runs (Supplementary Table 10).

### Validation of clinically significant alterations in mutation-positive tumour samples

The quality control (QC) metrics and sensitivity of the EXaCT-1 assay to detect clinically significant mutations were also demonstrated by analysing 57 mutation-positive clinical samples, representing a large spectrum of tumours and sample types. The fraction covered at >10× was 92% (s.d.=3%). The total allele count varied widely by variant type, with a mean total and alternate allele counts of 87 (s.d.=72) and 72 (s.d.=38), respectively. Of these, *KRAS* mutations particularly showed low-level coverage with a mean total and alternate alleles counts of 17 and 5, respectively, likely owing to a paucity of restriction enzyme sites in that region (Supplementary Table 2). As shown below, this can result in need to retest this gene using an alternative approach. The observed mean average read count log2 ratio for HER2/Neu was 1.73 (range: 0.68–3.68), corresponding to ~6.5 copies, as measured by droplet digital PCR.

**Figure 6:**
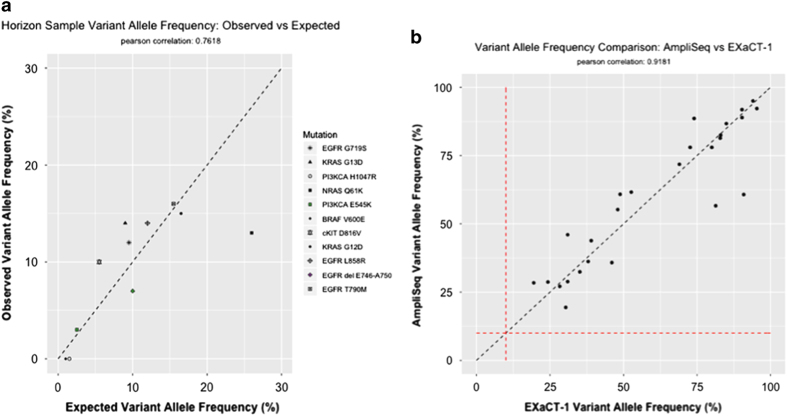
EXaCT-1 mutation correlation studies. (**a**) EXaCT-1 performance was validated using Reference FFPE DNA (Horizon sample) covering 11 onco-relevant variants at allele frequencies ranging from 2–25% across 6 genes commonly mutated in cancer. VAF results demonstrate a good agreement (Pearson correlation=0.76) between EXaCT-1 AF results and the expected reference DNA results. (**b**) Concordance between EXaCT-1 and Ion Torrent AmpliSeq assay across 9 genes (26 mutations) in 22 clinical cancer samples. The results demonstrate a good agreement between both tests over a wide range of allele frequencies with a Pearson correlation values of 0.92.

Additional validation of EXaCT-1 using clinical samples and AmpliSeq assay demonstrated high correlation (Pearson correlation=0.92) in VAF calls between the two methods across additional 22 samples and 26 SNVs/indels in 9 genes (Figure 6a, Supplementary Table 11). The lower correlation (Pearson correlation=0.76) observed for the commercial reference DNA was primarily due to the presence of mutations with VAFs ⩽10% (Figure 6b, Supplementary Table 12). All gene variations were detected using our pipeline when the VAF was ⩾10%. Variations present at lower percentage were not reliably detected by our test (as expected, based on our reported LoD of 10% VAF).

### Report generation

For each patient, a report in PDF format is generated as described in Materials and Methods. Each report includes the entire mutation content divided into three Tiers (clinically actionable mutations as Tier 1, other genomic alterations in cancer genes in Tier 2, variants of unknown significance in Tier 3). Interpretation for actionable events are drawn from an in-house Precision Medicine Knowledge Base (https://pmkb.weill.cornell.edu). The report also include sample identifiers, sample type (FFPE or fresh-frozen) diagnosis and histology, and date of sample collection. It also reports overall QC metrics such as number of reads and average coverage. Finally, a report add-on includes CNA profiles as well as Integrated Genome Viewer automated screenshots for each mutation detected and for the four representative genes (*EGFR*, *KRAS*, *BRAF*, and *JAK2*). Representative examples of a report and a CNA profile in a solid tumour specimen presented as Log2 Ratio of fold change at different chromosomes are presented in Supplementary Files 1 and 2.

### Quality control and quality assurance

In routine clinical runs QC metrics (depth of coverage, allelic read percentage and strand bias) are carefully reviewed to ensure they meet the analysis criteria listed in the Materials and Methods. Each and every run also includes a mutation-positive sensitivity control with at least three variants with AF next to the LoD of 10–20% and a mutation-negative control (NA12878), in addition to a non-template (water) DNA monitoring for PCR contamination. The sensitivity control is rotated between runs to test the principal significant mutations. These controls are also used to monitor for run-to-run variability and reproducibility over time using Levy–Jennings (LJ) plots, allowing identification of trends and patterns. Valid sensitivity control values must fall within ±3 s.d. of the expected mean value. Examples of LJ plots for the mutation-positive sensitivity controls are shown in the Supplementary Data (Supplementary Figure 5).

### Significant alterations in a large clinical cohort

**Figure 7:**
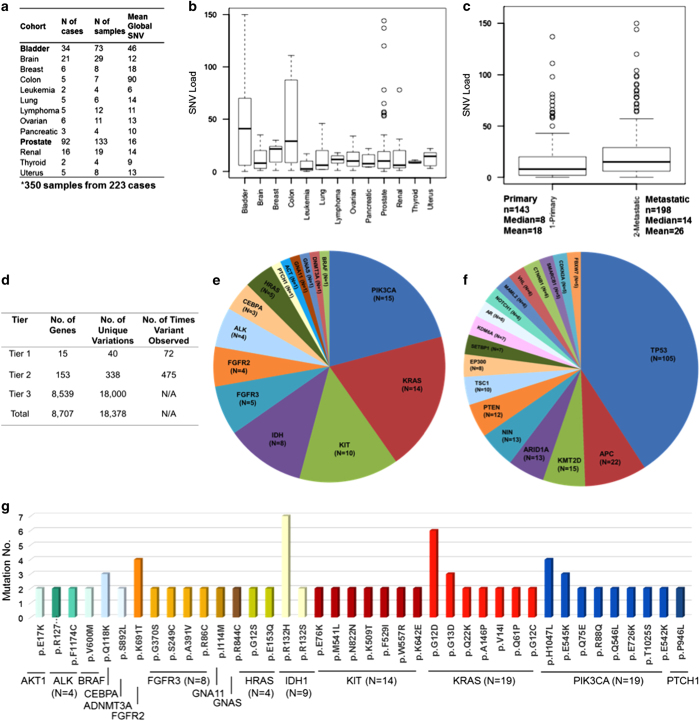
EXaCT-1 mutation testing statistics in IPM patient cohort. (**a**) Number of cases by cancer type, (**b**) mutation load by cancer diagnosis and (**c**) stage. (**d**) Number of gene variations by gene and by tier. (**e**) Commonly mutated Tier 1 and (**f**) Tier 2 genes (shown are 17 most commonly occurring genes out of 153 Tier 2 cancer genes). (**g**) Spectrum of unique mutations (*N*=40) in most commonly mutated Tier 1 cancer genes (*N*=15). IPM, Institute for Precision Medicine.

Using our NYS-DOH accredited EXaCT-1 we have analysed 337 prospective tumour specimens (primary and metastatic) from a wide range of cancer patients with advanced disease received at our Englander Institute for Precision Medicine (IPM) with an average coverage of 86× (Figure 7a–c). Overall, a total of 18,378 unique gene variations (SNV/indels) were identified in 8,707 genes, with an average of 16 mutations/case. Mutations were identified in 168 unique genes out of the 558 cancer genes analysed (30%). Among these, 72 mutations were Tier 1 (40 unique mutations in 15 genes) and 475 were Tier 2 (338 unique variations in 153 genes), accounting for 13% and 69% of the cases, respectively (Figure 7d). In Tier 1, *PIK3CA*, *KRAS* and *KIT* were the most commonly mutated genes, comprising 21%, 20% and 14% of the mutations identified, respectively. By, contrast, T*P53* was most frequently mutated gene in Tier 2, accounting for 22% of the mutations, followed by *APC* and *KMT2D,* comprising 5% and 3% of the mutations, respectively. These results demonstrate that EXaCT-1 can be reliably used to identify hotspot mutations as well as for the identification of less common and less well characterised variants.

## Discussion

NGS technology is poised to radically change clinical practice and trial design for cancer care. To date, most WES for precision cancer care has been performed in a research setting. These studies provide a robust means of making important genomic discoveries. However, the assays and the computational approaches are complex and not well standardised. The computational pipelines vary significantly from study to study and even from sample to sample. Moreover, the new precision medicine initiative by the NIH will need to address the rigors of clinical testing.^13^ These efforts will potentially include new guidelines through the FDA for test performance and approval. Here we describe the development and validation of EXaCT-1 comprised of WES, using a standard protocol and the associated computational pipeline that leads to a CLIA-compliant patient report. The process described for the development of EXaCT-1, due to the rigorous test development requirements by NYS-DOH, more closely resembles the process that will be required by the FDA for genomic testing. Today, only NYS requires rigorous test results as part of a review process prior to approval. Although a number of organisations are concerned that an unreasonable burden will be placed on the laboratories for developing a FDA-approvable test, we demonstrate here that it is manageable and would be the expected requirement in NYS. The new assay is suitable for WES in cancer with an overall assay sensitivity and specificity of 99.9% and reportable range of 95.7% at ⩾30× coverage. This approach incorporates new algorithms for estimation of neoplastic content, testing and for identification, processing and reporting of somatic alterations. Since development, we have used it as part of a proof-of-concept clinical trial for patients with advanced cancer care.^15^ To date we have evaluated 337 patients with EXaCT-1, and have analysed primary tumours as well as metastatic tumours that have undergone treatment following standard of care therapy. Prospective analysis of cases demonstrated the utility of EXaCT-1 in the clinical setting, allowing identification of known actionable mutations, or unanticipated, potentially relevant novel mutations in 82% of cases.

EXaCT-1 is based on HaloPlex library preparation technology^16^ in which circulated biotinylated DNA-probe hybrids are amplified by PCR, providing an enriched and barcoded DNA product for sequencing. The method uses a novel Q5 High-Fidelity DNA Polymerase (New England Biolabs Inc, Ipswich, MA, USA), optimised to reduce errors and produce even amplification of NGS libraries, regardless of GC content. HaloPlex also requires low amounts of starting DNA in par with other hybrid capture methods. In addition, it shows a high capture efficiency as defined by the fraction of mapped reads on target (90.0% on average) and high sensitivity in SNV detection.^17^ Finally, in our hands the HaloPlex-WES assay performed equally well for FFPE and non-FFPE DNA samples, requiring significantly less amounts of starting DNA material and allowing the completion of libraries (1.5 days) and sequencing in as little as 3.5 days. This assay (2×100 bp sequencing based on HiSeq 2500 rapid mode) enables a 7-day turnaround time suitable for sequencing of urgent samples at a reasonable reagent cost (paired normal/tumour) of ~$1,400.

In this study, an average of 225 ng and a minimum of 500 ng of high-molecular-weight (HMW) DNA and FFPE DNA, respectively, were required for achieving high quality sequencing with an average capture and fraction covered ⩾10× of >90%, respectively. Van Allen *et al.*^18^ have reported the use of significantly smaller amounts of FFPE DNA by their hybrid-selection based assay, but to meet their QC metrics of ⩾80% capture rate with at least 20× coverage and ⩾100×, a higher amount of additional sequencing was required due to an increased fraction of duplicate molecules in the library.^18^

EXaCT-1 was also designed to generate sequencing where the percentage of targeted bases with no coverage is very low. Coverage, depending on the total number of reads generated, the adaptor removal algorithm and trimming, is robust and shows little variation between experiments. In our hands, only <1% of the control DNA (*N*=45) samples had <10% coverage. Green *et al*.,^19^ have recently showed that patients’ DNA samples were successfully sequenced after HaloPlex capture, with >99% of targeted nucleotides in the panel covered by >20×.^19^ Similar results were obtained by Berglund *et al*.,^16^ demonstrating that virtually all SNVs in their study (>99%) were covered by at least one sequence read and >96% of the variants were covered at a sequence depth of at least 30×. However, both of these studies have been using HMW DNA and targeted HaloPlex panels. To best of our knowledge, our study is the first WES study evaluating the performance of HaloPlex exome-capture enrichment technology primarily focusing on FFPE DNA samples.

Finally, we recognise that as all assays are improving on a quarterly basis other NGS platforms or versions of assay we tested earlier may now demonstrate similar or better performance characteristics. Recently, Alioto *et al.*^20^ have demonstrated the utility of whole-genome sequencing for cancer genome analysis at an increasing depth of up to 100×, detecting 95% of the mutations analysed. However, usage of such novel assays in clinical practice will require extensive validation. We hope that regular comparison of the data across laboratories will provide important insights to meaningful approach at standardisation that are critical to the precision medicine initiative.^13^

Our comprehensive approach for validating the performance of EXaCT-1 included measuring accuracy, reportable range, analytical sensitivity, specificity and correlation with mutation-positive controls using commercial material and patient samples. The overall performance of EXaCT-1 was high. The overall assay sensitivity, specificity and positive predictive value measured with reference material were 95.7%, 99.9% and 99.2%, respectively, across all exomes. The LoD for validated mutations was 10% at minimum 30× unique coverage with false-negative calls primarily observed with VAFs<10%. Importantly, no false-positive calls in target genes were observed, as the lack of mutations is as important for precision care and for properly directing therapy to the patient. However, we realise that due to tumour heterogeneity and the presence of low frequency mutations, somatic variants present at AFs <10% may be missed by the current pipeline. When convincing evidence of a mutation’s presence exists, further testing using a sensitive approach (droplet digital PCR; AmpliSeq test) is requested and report amended to include orthogonal method testing results.

In routine clinical practice, EXaCT-1 QC is performed at multiple pre- and post-analytical points in the workflow, including review of hematoxylin and eosin-stained tissue sections for assessing sample quality and neoplastic content, enrichment of tumour samples by macrodissection and evaluation of the run QC metrics. Tumour purity is also assessed computationally by CLONET, allowing more accurate estimation of tumour purity and ploidy in the tumour samples, especially in cases of low tumour burden.^21^ In addition, to make review of the EXaCT-1 data effective and accurate, we have curated published gene variants associated with treatment or clinical outcome (http://pmkb.weill.cornell.edu), integrated publicly available and laboratory-developed database that links variant with annotated interpretation based on medical literature and included paired analysis of germline/tumour limiting the number of possible pathogenic variants. Moreover, we integrated the data into electronic reports and cBioPortal for Cancer genomics (http://www.cbioportal.org/), providing means of following patients along the course of their clinical care. This will ensure that when new drugs become available, new understanding of mutations emerge, our tests can be rescanned for evidence of new clinical treatment opportunities. The EXaCT-1 test may also be beneficial for evaluating germline alterations as we sequence both the tumour and normal DNA. One might imagine that the identification of novel variants (single nucleotide polymorphisms) associated with drug response may be useful to patients’ cancer care as well as general clinical care. Finally, other novel biomarkers may emerge from the WES data that could be readily evaluated. For example, immunotherapy shows tremendous promise,^22^ yet biomarkers are lacking. We can envisage developing biomarkers based on the patient’s tumour or germline DNA to help address this unmet challenge.

Although WES has strong advantages, there are also some limitations. Focused sequencing for most of the targeted NGS panels achieves coverage at 500–1,000×, whereas total coverage for WES assays is only 100× or less. The technology also does not cover each and every exon. A small number of exons, such as those buried in stretches of repeats out towards the chromosome tips, or in duplicated regions are not covered. In our hands, ~1% of the HaloPlex exome is poorly covered with <10 reads per target base, likely due to low mapability.^16^ Furthermore, approximately one-fifth (~23% on average) of the captured regions in clinically relevant genes did not achieve the required minimum depth of coverage for accurate negative-mutation calls. This means that it is in some cases difficult to completely exclude the presence of low abundance, near-detection threshold mutations in our assay. This limitation is most acute for tumour suppressor genes, where deleterious mutations could potentially occur anywhere along the entire length of the coding region. In other words, it may be difficult to accurately report such genes as not mutated even when no mutated reads are found. This issue is currently addressed, at least in part, in two ways: (1) by the report add-on appendix (see Report generation, Results section) detailing coverage QC metrics below threshold for any cancer gene-related mutations reviewed by the molecular pathologist attending at sign-out in conjunction with the final report and (2) by maintaining a growing list of tumour-type-specific pertinent negative variants and genes in the Precision Medicine Knowledge Base, and thereby facilitating incorporation of specific warnings and request for confirmatory testing when suboptimal coverage is found in key genes.

Interestingly, 0.41% of the exons in this analysis had no coverage at all. This is possibly owing to coverage gap affected by the location of restriction enzyme sites used for fragmentation and differences in the designed and actual library insert size. Coonrod *et al.*^23^ have demonstrated that the sizes of all sequenced inserts differed from the distribution of the designed ones, potentially leading to underrepresentation in the sequence data if an insert was >300 bp. Future versions of EXaCT-1 will include enriched areas of coverage for the most clinically significant genes. This spiked WES platform should act as a hybrid between WES and targeted testing providing the broad coverage needed for advanced cancer care and taking full advantage of the other feature available thought WES without compromising coverage provided by targeted capture. Finally, despite the increased demand and proven utility of WES, routine whole exome is still associated with many challenges including the data generation and interpretation, and manipulation and storage of the data, increasing the costs of the testing and requiring highly trained health-care professionals as well as special solutions for data management such as cloud storage facilities. This will require the development of new guidelines and standards that address issues of the data analysis, management of the data as well as the data safety.^24^

In summary, we have developed EXaCT-1, an accurate, sensitive and specific method for identifying actionable mutations that guide precision cancer treatment, providing additional genomic sequencing options for precision medicine cancer care. The study also provides a paradigm for WES validation using a broad selection of tumour types, and to our knowledge providing the largest comprehensive study of WES for cancer care in the clinical laboratory.

## Materials and methods

For detailed methods and associated references regarding the samples and controls used the study, sample processing, NGS, data analysis, confirmatory studies and report generation, see Supplementary Methods.

## Figures and Tables

**Table 1:** Accuracy of EXaCT-1 using HapMap DNA 12878 (*n*=2)

	*Benchmark data set*		
	*Non-reference sites (positive)*	*Reference sites (negative)*	*Total*
*EXaCT-1*			
Non-reference sites (positive)	17,144.5	148.5	17,293
Reference Sites (negative)	772.5	27,858,875.5	27,859,648
Total	17,917	27,859,024	27,876,941

Abbreviation: EXaCT-1, Exome Cancer Test v1.0.

**Table 2:** Analytic sensitivity, specificity and positive predictive value of EXaCT-1 vs. Benchmark data set

	*Sensitivity (%)*	*95% CI*	*Specificity (%)*	*95% CI*	*PPV (%)*	*95% CI*
NA12878-1	95.5	95.2–95.8	99.9995	99.9994–99.9996	99.2	99.0–99.3
NA12878–2	95.8	95.5–96.1	99.9994	99.9994–99.9995	99.1	98.9–99.2

Sensitivity, TP/(TP+FN); Specificity, TN/(TN+FN); PPV, TP/(TP+FP).

Abbreviations: CI, confidence interval; EXaCT-1, Exome Cancer Test v1.0; FP, false positive; FN, false negative; PPV, positive predictive value; TN, true negative; TP, true positive.
