# Supplementary material for: Development and validation of a whole-exome sequencing test for simultaneous detection of point mutations, indels and copy-number alterations for precision cancer care
Source: NPJ Genom Med. 2016 Jul 20;1:16019–. doi: 10.1038/npjgenmed.2016.19 (PMC5539963; doi:10.1038/npjgenmed.2016.19)
Supplement: Supplementary Information [file npjgenmed201619-s1.doc]

**Supplementary Information**

**Development and Validation of a Whole Exome Sequencing Test-1 (EXaCT-1) for Simultaneous Detection of Point Mutation, Indels and Copy Number Alterations for Precision Cancer Care**

H. Rennert^1,2^, K. Eng^1,3^, T. Zhang^1,4^, A. Tan^1,4^, J. Xiang^1,4^, A. Romanel^6^, R. Kim^1,2^, W. Tam^2^, Y. Liu^2^, B. Bhinder^1^, J. Cyrta H.^1^, Beltran^1,5^, B. Robinson^1,2^, J.M. Mosquera^1,2^, H. Fernandes^1,2^, F. Demichelis^6^, A. Sboner^1,2,3^, M. Kluk^1,2^, M. A. Rubin^1,2^*, and O. Elemento^1,3^*

^1^ Institute for Precision Medicine, New York Presbyterian Hospital-Weill Cornell Medicine. New York, NY.

^2^ Department of Pathology and Laboratory Medicine, Weill Cornell Medicine, New York, NY.

^3^ Institute for Computational Biomedicine, Weill Cornell Medicine, New York, NY.

^4^ Genomics Core Facility, Weill Cornell Medicine, New York, NY.

^5^ Department of Medicine, Division of Hematology and Medical Oncology, Weill Cornell Medicine, New York, NY.

^6^ CIBIO, University of Trento, Trento, Italy

**Supplementary Figures**

Suppl. Figure 1.


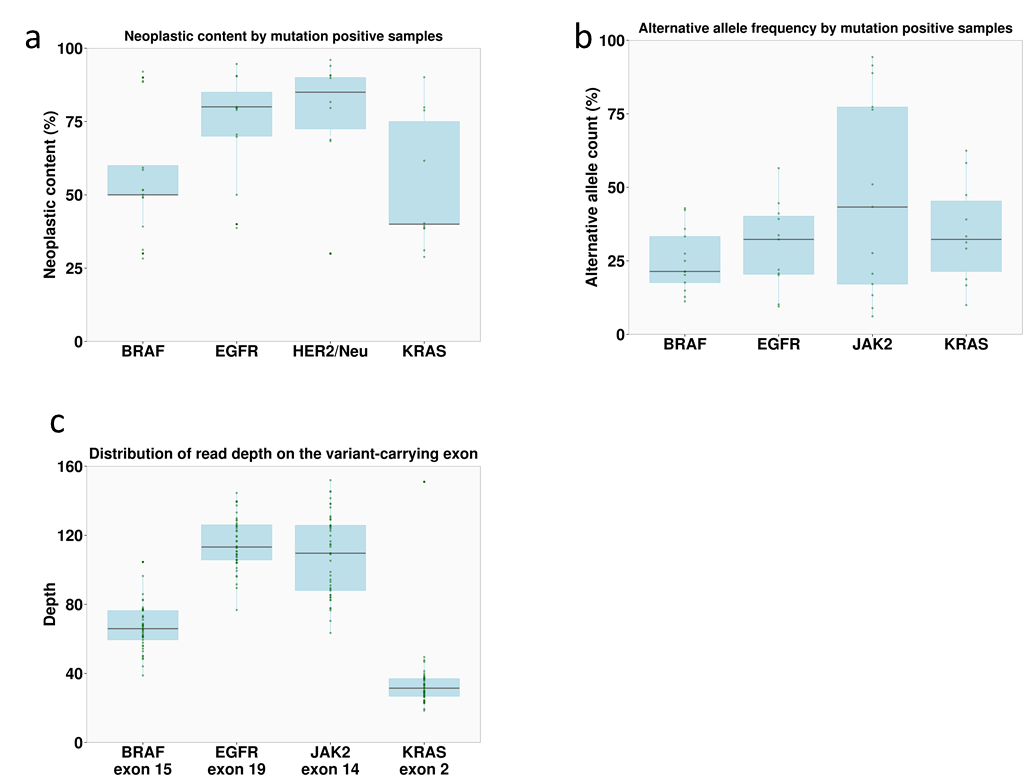


Suppl. Figure 1. Neoplastic content and VAF metrics by mutation-positive sample in 41 FFPE clinical samples. The neoplastic content (a) and VAF (b) ranged between 30%-90% and 10%- 63%, respectively and did not differ significantly by mutation-positive sample. The read depth on the variant-carrying exon was significantly lower for KRAS exon 2 mutations compared to *BRAF*, *EGFR* or *JAK2* mutations (c).

Suppl. Figure 2.


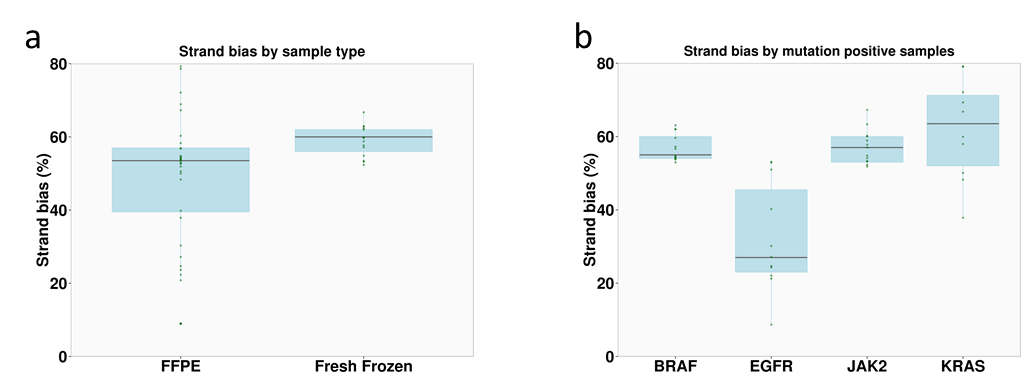


Suppl. Figure 2. EXaCT-1 strand bias by sample type (a) and mutation-positive sample (b). A higher strand bias on average is observed for the *EGFR del19* mutations due to the mutation type (CNV).

Suppl. Figure 3.


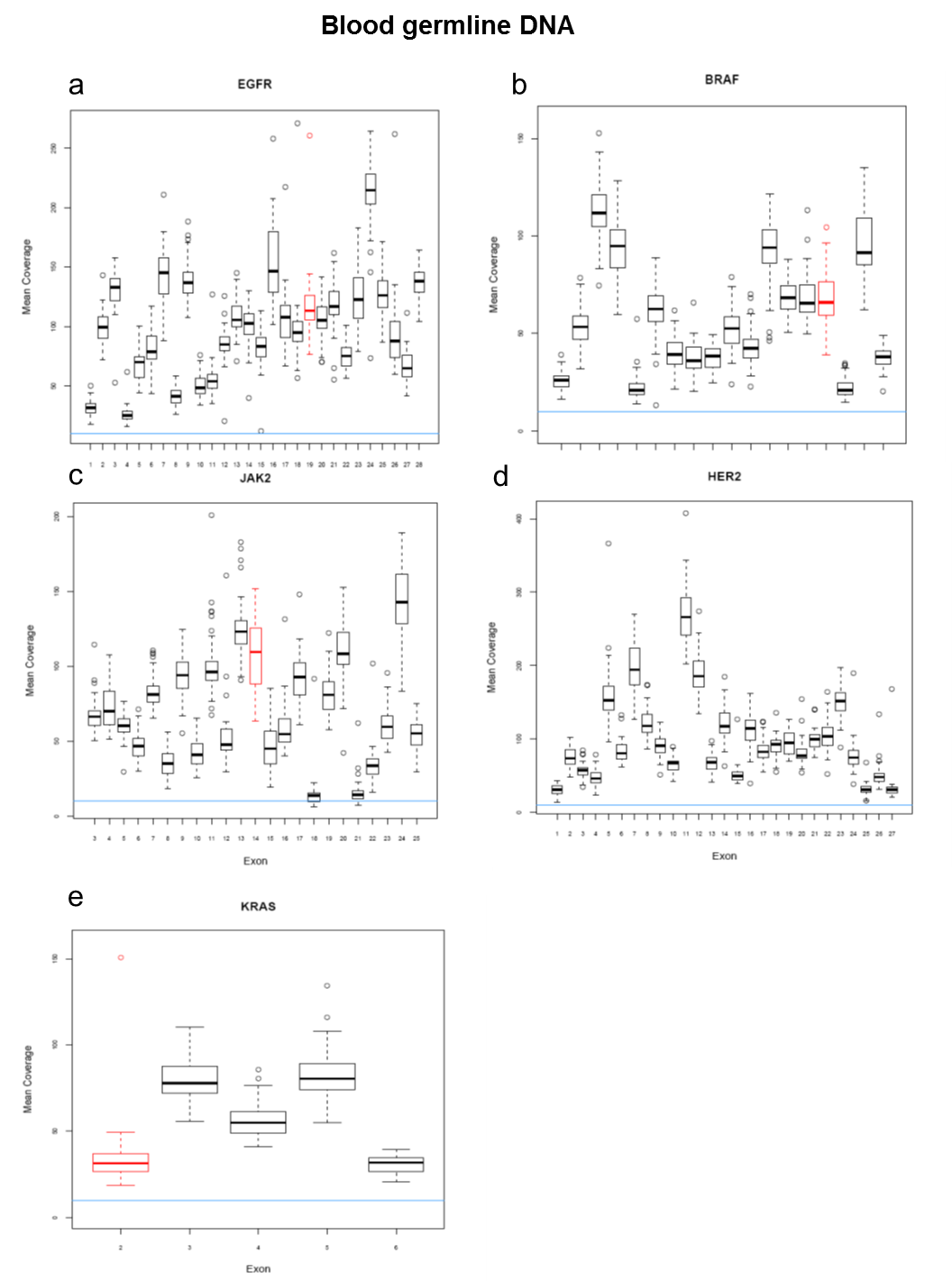


Suppl. Figure 3. EXaCT-1 Validation. *EGFR, BRAF JAK2, HER2* and *KRAS* coverage by exon. The EXaCT-1 WES test was validated using 86 archival samples including 45 HMW gDNA (N=45) and mutation-positive FFPE DNA (N=41) from a diverse representation of solid tumors and hematological cancers. Illustrated here are representative examples of (A) *EGFR*, (B) *HER2*, (C) *JAK2*, (D) *HER2* and (E) *KRAS* coverage by exon in HMW DNA used for NYS CLIA application of ExACT-1. The red box indicates coverage of the Exon-harboring mutations by gene. The blue horizontal line above the x-axis indicates depth coverage of 10X.

Suppl. Figure 4.


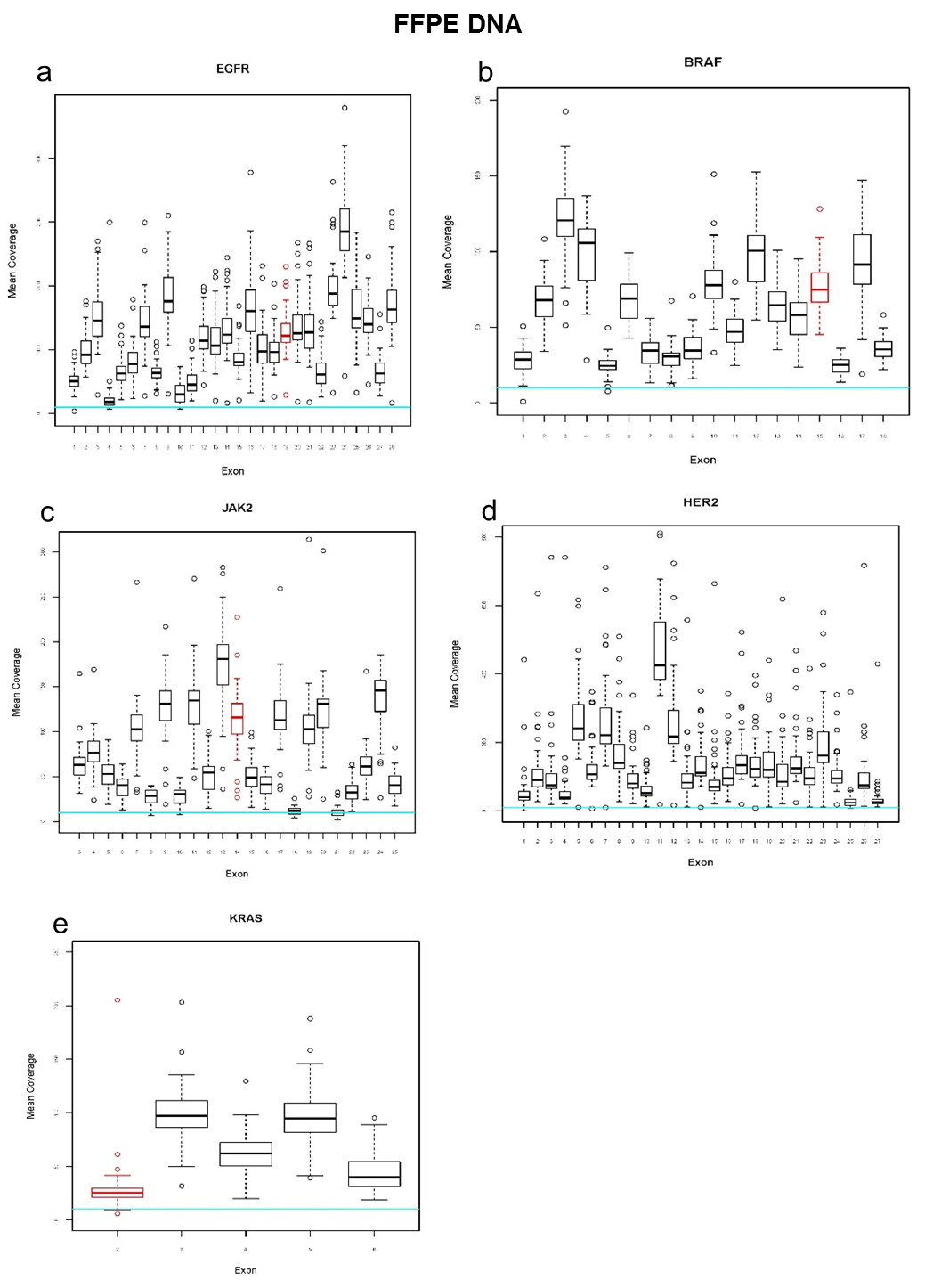


Suppl. Figure 4. EXaCT-1 Validation. *EGFR, BRAF JAK2, HER2* and *KRAS* coverage by exon. The EXaCT-1 WES test was validated using 86 archival samples comprising HMW gDNA (N=45) and mutation-positive FFPE DNA (N=41) from a diverse representation of solid tumors and hematological cancers. Illustrated here are representative examples of (A) *EGFR*, (B) *HER2*, (C) *JAK2*, (D) *HER2* and (E) *KRAS* coverage by exon in FFPE DNA used for NYS CLIA application of ExACT-1. The red box indicates coverage of the Exon-harboring mutations by gene. The blue horizontal line above the x-axis indicates depth coverage of 10X.

Suppl. Figure 5.


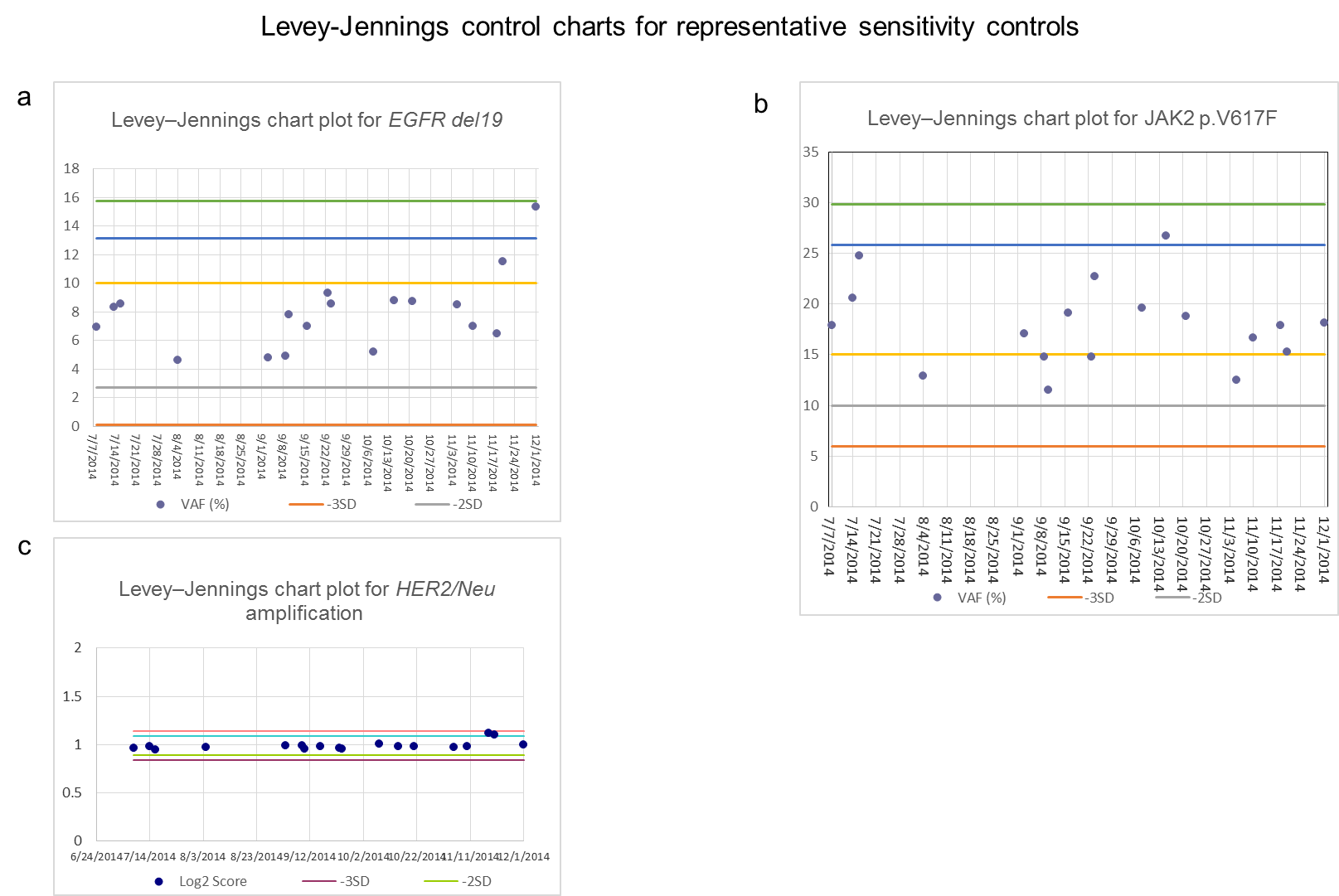


Suppl. Figure 5. Levey-Jennings charts for representative sensitivity controls. The performance of the assay to detect specific genetic alteration (JAK2 p.V617F, *EGFR del19* and *HER2/neu* amplification) has been be closely monitored for variation over time using cell line DNA sensitivity controls. Data values demonstrated consistent assay performance within the allowable limits of measurement error*.* The X axis displays time or sequential run. The Y axis shows the value of each sensitivity control result. A horizontal line above the X axis is centered around the target mean with upper and lower control limits of +/-2 SDs and+/- 3 SDs marked for easy viewing. The sensitivity controls targets were 15%, 10% and a mean read count log2 ratio of 0.9 for *JAK2* mutation, *EGFR del19*, and *HER2/Neu* amplification, respectively.

**Supplementary Tables**

Suppl. Table 1. Quality metrics of the HaloPlex WES assay on the Illumina platform using HAPMap DNA

| Sample No. | Gene | Neoplastic Content (%) | Total Reads | Captured Reads | % Captured Reads | Average Coverage (Read No. Mb) | Fraction Covered>=10X | Strand Bias | MQ<=20 Reads |
| --- | --- | --- | --- | --- | --- | --- | --- | --- | --- |
| 1 | NA12878_1 | N/A | 67980845 | 56688175 | 83% | 81 | 94% | N/A | 0 |
| 2 | NA12878_2 | N/A | 79101538 | 66491297 | 84% | 96 | 94% | N/A | 0 |
| 3 | NA12878_3 | N/A | 66350868 | 55919925 | 84% | 83 | 94% | N/A | 0 |
| 4 | NA19240 | N/A | 68206111 | 57215305 | 84% | 85 | 94% | N/A | 0 |
| Average | HAPMap DNA | N/A | 70409841 | 59078676 | 84% | 86 | 94% | N/A | 0 |
| SD |  |  | 5068957 | 4304396 |  |  |  |  |  |

Suppl. Table 2. Quality metrics and assay performance characteristics for 57 tumors and mutations validated in this study

| Sample No. | Gene | Sample Type | Neoplastic Content (%) | Total Reads | Captured Reads | %Captured Reads | Avg. Cov. | Fraction Covered>=10X | Strand Bias | MQ<=20 Reads | Avg. Read Count Log2 Ratio | Total Allele Count | ALT Allele Count | %ALT  AF |
| --- | --- | --- | --- | --- | --- | --- | --- | --- | --- | --- | --- | --- | --- | --- |
| 1 | *BRAF* | FFPE | 30 | 84812304 | 76169127 | 90% | 108 | 93% | 53% | 0 | N/A | 109 | 30 | 27.5% |
| 2 | *BRAF* | FFPE | 60 | 56891535 | 51732685 | 91% | 73 | 88% | 57% | 0 | N/A | 56 | 12 | 21.4% |
| 3 | *BRAF* | FFPE | 30 | 62207069 | 58647371 | 94% | 87 | 88% | 55% | 0 | N/A | 78 | 10 | 12.8% |
| 4 | *BRAF* | FFPE | 50 | 77374746 | 69096938 | 89% | 97 | 92% | 54% | 0 | N/A | 72 | 18 | 25.0% |
| 5 | *BRAF* | FFPE | 90 | 71685879 | 64343955 | 90% | 90 | 91% | 54% | 0 | N/A | 89 | 10 | 11.2% |
| 6 | *BRAF* | FFPE | 90 | 68408189 | 61104839 | 89% | 85 | 92% | 55% | 0 | N/A | 75 | 16 | 21.3% |
| 7 | *BRAF* | FFPE | 40 | 74808224 | 66836276 | 89% | 94 | 92% | 57% | 0 | N/A | 47 | 7 | 14.9% |
| 8 | *BRAF* | FFPE | 90 | 75792892 | 68312361 | 90% | 96 | 92% | 54% | 0 | N/A | 85 | 15 | 17.6% |
| 9 | *BRAF* | FFPE | 60 | 72804168 | 66631670 | 92% | 93 | 89% | 54% | 0 | N/A | 94 | 19 | 20.2% |
| 10 | *BRAF* | Fresh Frozen | 50 | 72559899 | 60799540 | 84% | 91 | 94% | 63% | 0 | N/A | 92 | 33 | 35.9% |
| 11 | *BRAF* | Fresh Frozen | 50 | 69365236 | 58284130 | 84% | 87 | 94% | 62% | 0 | N/A | 63 | 21 | 33.3% |
| 12 | *BRAF* | Fresh Frozen | 50 | 60340859 | 50810482 | 84% | 76 | 93% | 60% | 0 | N/A | 77 | 33 | 42.9% |
| 13 | *BRAF* | Fresh Frozen | 50 | 64856238 | 54990414 | 85% | 82 | 93% | 62% | 0 | N/A | 52 | 22 | 42.3% |
| 14 | *EGFR* | FFPE | 50 | 68201823 | 63049621 | 92% | 96 | 92% | 53% | 0 | N/A | 460 | 189 | 41.1% |
| 15 | *EGFR* | FFPE | 70 | 67585724 | 63356418 | 94% | 94 | 89% | 40% | 0 | N/A | 58 | 12 | 20.7% |
| 16 | *EGFR* | FFPE | 90 | 78189768 | 70274519 | 90% | 108 | 95% | 51% | 0 | N/A | 118 | 12 | 10.2% |
| 17 | *EGFR* | FFPE | 40 | 70076008 | 64991436 | 93% | 98 | 92% | 53% | 0 | N/A | 101 | 34 | 33.7% |
| 18 | *EGFR* | FFPE | 80 | 71022375 | 61574726 | 87% | 88 | 92% | 22% | 0 | N/A | 82 | 18 | 22.0% |
| 19 | *EGFR* | FFPE | 80 | 73761781 | 63342435 | 86% | 90 | 93% | 24% | 0 | N/A | 99 | 32 | 32.3% |
| 20 | *EGFR* | FFPE | 90 | 84081652 | 73515821 | 87% | 104 | 94% | 9% | 0 | N/A | 61 | 24 | 39.3% |
| 21 | *EGFR* | FFPE | 95 | 70117108 | 61726732 | 88% | 90 | 92% | 25% | 0 | N/A | 108 | 61 | 56.5% |
| 22 | *EGFR* | FFPE | 70 | 87128545 | 74595592 | 86% | 106 | 94% | 30% | 0 | N/A | 126 | 12 | 9.5% |
| 23 | *EGFR* | FFPE | 80 | 92026929 | 79259503 | 86% | 113 | 94% | 27% | 0 | N/A | 109 | 22 | 20.2% |
| 24 | *EGFR* | FFPE | 80 | 103425904 | 89806864 | 87% | 128 | 94% | 21% | 0 | N/A | 177 | 79 | 44.6% |
| 25 | *HER2/Neu* | FFPE | 95 | 75392032 | 66803555 | 89% | 96 | 91% | 50% | 0 | 0.9668 | N/A | N/A | N/A |
| 26 | *HER2/Neu* | FFPE | 70 | 62129177 | 59512026 | 96% | 85 | 82% | 50% | 0 | 3.0988 | N/A | N/A | N/A |
| 27 | *HER2/Neu* | FFPE | 30 | 78258098 | 71776356 | 92% | 110 | 94% | 50% | 0 | 0.6756 | N/A | N/A | N/A |
| 28 | *HER2/Neu* | FFPE | 90 | 74597265 | 68953561 | 92% | 104 | 92% | 50% | 0 | 1.5559 | N/A | N/A | N/A |
| 29 | *HER2/Neu* | FFPE | 90 | 74451749 | 70182371 | 94% | 104 | 89% | 50% | 0 | 1.6296 | N/A | N/A | N/A |
| 30 | *HER2/Neu* | FFPE | 70 | 63375969 | 59653299 | 94% | 87 | 86% | 50% | 0 | 0.9932 | N/A | N/A | N/A |
| 31 | *HER2/Neu* | FFPE | 95 | 83517501 | 73693157 | 88% | 105 | 93% | 50% | 0 | 3.6776 | N/A | N/A | N/A |
| 32 | *HER2/Neu* | FFPE | 80 | 70147884 | 64322359 | 92% | 91 | 86% | 50% | 0 | 0.7607 | N/A | N/A | N/A |
| 33 | *HER2/Neu* | FFPE | 90 | 75278665 | 69112318 | 92% | 96 | 89% | 50% | 0 | 1.4906 | N/A | N/A | N/A |
| 34 | *HER2/Neu* | Fresh Frozen | 80 | 71073768 | 59406611 | 84% | 84 | 94% | 50% | 0 | 2.4592 | N/A | N/A | N/A |
| 35 | *JAK2* | Fresh | N/A | 82788269 | 71655286 | 87% | 110 | 96% | 58% | 0 | N/A | 123 | 11 | 8.9% |
| 36 | *JAK2* | FFPE | N/A | 62498597 | 57603135 | 92% | 80 | 85% | 52% | 0 | N/A | 51 | 26 | 51.0% |
| 37 | *JAK2* | FFPE | N/A | 61013039 | 55896335 | 92% | 78 | 86% | 54% | 0 | N/A | 82 | 14 | 17.1% |
| 38 | *JAK2* | Fresh Frozen | N/A | 74038205 | 62449286 | 84% | 89 | 94% | 67% | 0 | N/A | 106 | 81 | 76.4% |
| 39 | *JAK2* | Fresh Frozen | N/A | 57894144 | 48526984 | 84% | 69 | 92% | 52% | 0 | N/A | 128 | 117 | 91.4% |
| 40 | *JAK2* | Fresh Frozen | N/A | 68289869 | 58004906 | 85% | 83 | 93% | 59% | 0 | N/A | 135 | 18 | 13.3% |
| 41 | *JAK2* | Fresh Frozen | N/A | 73265332 | 62298750 | 85% | 88 | 94% | 57% | 0 | N/A | 123 | 34 | 27.6% |
| 42 | *JAK2* | Fresh Frozen | N/A | 60859606 | 51778604 | 85% | 73 | 93% | 55% | 0 | N/A | 102 | 21 | 20.6% |
| 43 | *JAK2* | Fresh Frozen | N/A | 75084780 | 64544986 | 86% | 93 | 94% | 60% | 0 | N/A | 122 | 115 | 94.3% |
| 44 | *JAK2* | Fresh Frozen | N/A | 71920326 | 62723008 | 87% | 98 | 95% | 53% | 0 | N/A | 163 | 126 | 77.3% |
| 45 | *JAK2* | Fresh | N/A | 68347240 | 57217928 | 84% | 85 | 94% | 53% | 0 | N/A | 104 | 45 | 43.3% |
| 46 | *JAK2* | Fresh | N/A | 67061986 | 56368044 | 84% | 84 | 94% | 63% | 0 | N/A | 82 | 5 | 6.1% |
| 47 | *JAK2* | Fresh | N/A | 70742038 | 59164794 | 84% | 87 | 95% | 60% | 0 | N/A | 125 | 111 | 88.8% |
| 48 | *KRAS* | FFPE | 40 | 73380327 | 66736232 | 91% | 94 | 89% | 50% | 0 | N/A | 8 | 5 | 62.5% |
| 49 | *KRAS* | FFPE | 40 | 67839433 | 60456034 | 89% | 86 | 91% | 67% | 0 | N/A | 12 | 2 | 16.7% |
| 50 | *KRAS* | FFPE | 40 | 79335143 | 71217310 | 90% | 101 | 92% | 48% | 0 | N/A | 23 | 9 | 39.1% |
| 51 | *KRAS* | FFPE | 40 | 76666729 | 69421392 | 91% | 98 | 91% | 79% | 0 | N/A | 19 | 9 | 47.4% |
| 52 | *KRAS* | FFPE | 30 | 73834528 | 67285156 | 91% | 96 | 90% | 69% | 0 | N/A | 16 | 3 | 18.8% |
| 53 | *KRAS* | FFPE | 30 | 73444765 | 66570179 | 91% | 102 | 94% | 72% | 0 | N/A | 20 | 2 | 10.0% |
| 54 | *KRAS* | FFPE | 90 | 75782407 | 66620202 | 88% | 95 | 93% | 79% | 0 | N/A | 24 | 7 | 29.2% |
| 55 | *KRAS* | FFPE | 60 | 63025298 | 56734033 | 90% | 79 | 90% | 60% | 0 | N/A | 15 | 5 | 33.3% |
| 56 | *KRAS* | FFPE | 80 | 68320720 | 64033898 | 94% | 87 | 84% | 58% | 0 | N/A | 12 | 7 | 58.3% |
| 57 | *KRAS* | FFPE | 80 | 84063920 | 76141059 | 91% | 107 | 92% | 38% | 0 | N/A | 16 | 5 | 31.3% |
| Total Average |  |  | 66 | 72548134 | 64388011 | 91% | 93 | 92% | 52% | 0 | 1.73 | 87 | 32 |  |
| SD |  |  | 34 | 8486521 | 7561333 | 3% | 11.2 | 3% | 14% | 0.0 | 0.8 | 72 | 38 |  |

Suppl. Table 3. Formalin-fixed paraffin embedded (FFPE) DNA quality by GAPDH-PCR analysis

| Sample No. | Sample Name | Tumor Type | Tissue Type | Sample Type | Average Ratio of GAPDH | DNA Integrity Category |
| --- | --- | --- | --- | --- | --- | --- |
| 1 | PM106 | Thyroid Cancer | Thyroid | FFPE | 0.19 | B |
| 2 | PM107* | Thyroid Cancer | Thyroid | FFPE | 0.21 | A |
| 3 | PM114 | Thyroid Cancer | Thyroid | FFPE | 0.08 | B |
| 4 | PM70* | Colon Cancer | Colon | FFPE | 0.55 | B |
| 5 | PM71* | Colon Cancer | Colon | FFPE | 0.52 | A |
| 6 | PM74* | Colon Cancer | Colon | FFPE | 0.09 | B |
| 7 | PM81* | Colon Cancer | Colon | FFPE | 0.10 | B |
| 8 | PM82 | Colon Cancer | Colon | FFPE | 0.15 | B |
| 9 | PM83 | Colon Cancer | Colon | FFPE | 0.06 | B |
| 10 | PM178 | Colon Cancer | Colon | Fresh Frozen | NA | NA |
| 11 | PM179 | Colon Cancer | Colon | Fresh Frozen | NA | NA |
| 12 | PM180 | Colon Cancer | Colon | Fresh Frozen | NA | NA |
| 13 | PM181 | Colon Cancer | Colon | Fresh Frozen | NA | NA |
| 14 | PM125 | Lung adenocarcinoma | Lung | FFPE | 0.10 | B |
| 15 | PM126 | Lung adenocarcinoma | Lung | FFPE | 1.00 | A |
| 16 | PM127* | Lung adenocarcinoma | Lung | FFPE | 0.12 | B |
| 17 | PM128 | Lung adenocarcinoma | Lung - bone metastasis | FFPE | 0.10 | B |
| 18 | PM36* | Lung adenocarcinoma | Lung | FFPE | 0.58 | A |
| 19 | PM37* | Lung adenocarcinoma | Lung | FFPE | 0.65 | A |
| 20 | PM38* | Lung adenocarcinoma | Lung | FFPE | 0.22 | A |
| 21 | PM39 | Lung adenocarcinoma | Lung | FFPE | 0.42 | A |
| 22 | PM40* | Lung adenocarcinoma | Lung | FFPE | 0.13 | B |
| 23 | PM41 | Lung adenocarcinoma | Lung | FFPE | 0.35 | A |
| 24 | PM43 | Lung adenocarcinoma | Lung | FFPE | 0.23 | A |
| 25 | PM108 | Ductal carcinoma | Breast - Brain metastasis | FFPE | 0.55 | A |
| 26 | PM111 | Ductal carcinoma | Breast | FFPE | 0.07 | B |
| 27 | PM112 | Gastroesophageal adenocarcinoma | Esophagus and stomach | FFPE | 0.13 | B |
| 28 | PM116 | Ductal carcinoma | Breast | FFPE | 0.10 | B |
| 29 | PM129* | Ductal carcinoma | Breast | FFPE | 0.08 | B |
| 30 | PM130 | Ductal carcinoma | Breast | FFPE | 0.09 | B |
| 31 | PM97* | Gastroesophageal adenocarcinoma | Esophagus and stomach | FFPE | 0.19 | B |
| 32 | PM98* | Ductal carcinoma | Breast | FFPE | 0.32 | A |
| 33 | PM99* | Ductal carcinoma | Breast | FFPE | 0.07 | B |
| 34 | PM137 | Metastatic urothelial carcinoma | Inguinal mass | Fresh Frozen | NA | NA |
| 35 | PM53 | Myeloproliferative neoplasms | Peripheral Blood | Fresh | NA | NA |
| 36 | PM134 | Myeloproliferative neoplasms | Bone marrow | FFPE | 0.22 | A |
| 37 | PM135 | Myeloproliferative neoplasms | Bone marrow | FFPE | 0.19 | B |
| 38 | PM45 | Myeloproliferative neoplasms | Bone marrow | Fresh Frozen | NA | NA |
| 39 | PM46* | Myeloproliferative neoplasms | Bone marrow | Fresh Frozen | NA | NA |
| 40 | PM47 | Myeloproliferative neoplasms | Bone marrow | Fresh Frozen | NA | NA |
| 41 | PM48 | Myeloproliferative neoplasms | Bone marrow | Fresh Frozen | NA | NA |
| 42 | PM49 | Myeloproliferative neoplasms | Bone marrow | Fresh Frozen | NA | NA |
| 43 | PM50 | Myeloproliferative neoplasms | Bone marrow | Fresh Frozen | NA | NA |
| 44 | PM51 | Myeloproliferative neoplasms | Bone marrow | Fresh Frozen | NA | NA |
| 45 | PM165 | Myeloproliferative neoplasms | Peripheral Blood | Fresh | NA | NA |
| 46 | PM166 | Myeloproliferative neoplasms | Peripheral Blood | Fresh | NA | NA |
| 47 | PM167 | Myeloproliferative neoplasms | Peripheral Blood | Fresh | NA | NA |
| 48 | PM101 | Lung Cancer | Lung | FFPE | 0.21 | A |
| 49 | PM102* | Lung Cancer | Lung | FFPE | 0.21 | A |
| 50 | PM103* | Lung Cancer | Lung | FFPE | 0.15 | B |
| 51 | PM104* | Lung Cancer | Lung | FFPE | 0.14 | B |
| 52 | PM105* | Lung Cancer | Lung | FFPE | 0.16 | B |
| 53 | PM113 | Lung Cancer | Lung | FFPE | 0.43 | A |
| 54 | PM86* | Colon Cancer | Colon | FFPE | 0.21 | A |
| 55 | PM76 | Colon Cancer | Colon | FFPE | 0.18 | B |
| 56 | PM84 | Colon Cancer | Colon | FFPE | 0.05 | B |
| 57 | PM85 | Colon Cancer | Colon | FFPE | 0.09 | B |
| 58 | NA12878_1 | Negative | Peripheral Blood | Fresh | NA | NA |
| 59 | NA12878_2 | Negative | Peripheral Blood | Fresh | NA | NA |
| 60 | NA12878_3 | Negative | Peripheral Blood | Fresh | NA | NA |
| 61 | NA19240 | Negative | Peripheral Blood | Fresh | NA | NA |

Suppl. Table 4: Quality control Metrics by sample type across clinical validation dataset

| No. of samples | Sample type | Avg. neoplastic content (Range) | Avg. total reads | Avg. captured reads | Avg. % captured reads (Range) | Avg. coverage (Range) | Avg. fraction covered >=10X (Range) | Avg. strand bias (Range) | Avg. MQ<=20 reads (Range) |
| --- | --- | --- | --- | --- | --- | --- | --- | --- | --- |
| 41 | FFPE | 67%  (30-95%) | 73,823,314 | 66,612,021 | 96% (86-96%) | 96 (73-128) | 91% (82-95%) | 49% (9-79%) | 0 |
| 11 | Blood/BM | N/A | 70,026,527 | 59,521,143 | 85% (84-87%) | 87 (69-110) | 94% (92-96%) | 58% (52-67%) | 0 |
| 5 | fresh tissue | 56% (50-80%) | 67,639,200 | 56,858,235 | 84% (84-85%) | 84 (76-91) | 94% (93-94%) | 59% (50-63%) | 0 |
| Note: the numbers shown in the table were averaged over all samples of a given type: FFPE, Blood/BM and fresh tissue | | | | | | | | | |

Suppl. Table 5. Evaluation of index cross-talk

| Quality Metric | Homozygous reference sites (NA19240) and homozygous nonreference (NA12878) | Homozygous reference sites (NA12878) and homozygous nonreference (NA19240) |
| --- | --- | --- |
| Total positions (No.) | 2954 | 2247 |
| Total Reads/calls | 281679 | 225586 |
| Nonreference calls matching the other HapMap [*n/N* (%)] | 681/281679 (0.242) | 208/225586 (0.092) |
| Other nonreference calls [n/N (%)] | 105/281679 (0.037) | 35/225586 (0.016) |
| Fraction of any nonreference bases to total bases (%) | 0.279 | 0.108 |

Suppl. Table 6. Clinically actionable genes (Tier 1)

| *ABL1* | *BRCA1* | *ERBB2* | *GNAS* | *MCL1* |
| --- | --- | --- | --- | --- |
| *ABL2* | *BRCA2* | *ERBB3* | *HRAS* | *MET* |
| *AKT1* | *CD79B* | *ERBB4* | *IDH1* | *NRAS* |
| *AKT2* | *CDK4* | *FGFR1* | *IDH2* | *PDGFRA* |
| *AKT3* | *CDK6* | *FGFR2* | *IKZF1* | *PIK3CA* |
| *ALK* | *CDKN2A* | *FGFR3* | *JAK2* | *PTCH1* |
| *AR* | *CEBPA* | *FGFR4* | *KIT* | *PTEN* |
| *AURKA* | *CRKL* | *FLT3* | *KRAS* | *SMO* |
| *BCL2* | *DNMT3A* | *GNA11* | *MAP2K1* | *TSC1* |
| *BRAF* | *EGFR* | *GNAQ* | *MAP2K2* |  |

Suppl. Table 7. Distribution of coverage depth by gene (N=57)

| Gene | No. of Exons | Coverage Depth (X+SD) | Range |  |
| --- | --- | --- | --- | --- |
| *EGFR* | 28 | 103.3+9.52 | [77.5, 121.7] |  |
| *KRAS* | 5 | 57.5+8.00 | [44.6, 80.8] |  |
| *BRAF* | 18 | 60.3+6.43 | [43.3, 74.5] |  |
| *JAK2* | 23 | 70.9+8.18 | [54.3, 90.5] |  |
| *HER2/Neu* | 27 | 90.2+11.63 | [64.8, 121.2] |  |
| Note: |  |  |  |  |
| *JAK2* consists of 25 exons, the first two exons are not covered by the HaloPlex reagent; *KRAS* consists of 6 exons, the first exon is not covered by the HaloPlex reagent | | | | |
|  | | | | |

Suppl. Table 8. Cancer cell lines employed in mutation sensitivity studies

| Cell Lines | Catalog # | Mutation | Genomic Location | Total Reads | Captured Reads | Average Coverage | Fraction Covered >= 10X | Avg. Read Count Log2 Ratio | Total Allele Count | ALT Allele Count | ALT Allele Fraction |
| --- | --- | --- | --- | --- | --- | --- | --- | --- | --- | --- | --- |
| HEL 92.1.7 | ATCC-TIB-180 | *JAK2* p.V617F | chr9, 5073770 | 64303525 | 86.16% | 79X | 91% | N/A | 309 | 308 | 99.68% |
| PC-9 | BRC: RCB4455 | *EGFR* p.E746_A750del5 | chr7, 55241900-55243100 | 71033868 | 83.79% | 85X | 94% | N/A | 257 | 158 | 61.52% |
| SK-CO-1 | ATCC-HTB-39 | *KRAS* p.G12V | chr12, 25398284 | 72073078 | 83.47% | 86X | 93% | N/A | 53 | 42 | 79.25% |
| COLO-205 | ATCC-CCL-222 | *BRAF* p.V600E | chr7, 140453136 | 50526950 | 85.42% | 62X | 90% | N/A | 41 | 25 | 60.98% |
| BT474 | ATCC-HTB-20 | *HER2/neu* amplification | chr17, 37844393-37884915 | 60186725 | 85.37% | 74X | 91% | 3.5744 | N/A | N/A | N/A |

Suppl. Table 9. Analytical sensitivity studies by mutation

| Sample Mix | Gene (Mutation) | Tumor Percentage Mutation (Expected) by Ion Torrent | Calibrated Mutation Percentage | Total Reads | Captured Reads | Average Coverage | Total Allele Count | ALT Allele Count | ALT Allele Fraction (Observed) | Mean | SD |
| --- | --- | --- | --- | --- | --- | --- | --- | --- | --- | --- | --- |
| Mutant | *JAK2* p.V617F | 100.00% | 100.00% | 64303525 | 86.16% | 79X | 309 | 308 | 99.70% | / | / |
| Normal+ Mutant | *JAK2* p.V617F | 12.50% | 50.50% | 81889105 | 84.67% | 100X | 125 | 61 | 48.80% | 52.65% | 7.24% |
| Normal+ Mutant | *JAK2* p.V617F |  |  | 66394083 | 84.63% | 80X | 81 | 39 | 48.15% |  |  |
| Normal+ Mutant | JAK2 p.V617F |  |  | 64193084 | 84.40% | 77X | 100 | 61 | 61.00% |  |  |
| Normal+ Mutant | JAK2 p.V617F | 5.00% | 19.40% | 67570606 | 84.52% | 82X | 106 | 33 | 31.13% | 23.88% | 6.53% |
| Normal+ Mutant | *JAK2* p.V617F |  |  | 68635141 | 84.32% | 83X | 109 | 24 | 22.02% |  |  |
| Normal+ Mutant | *JAK2* p.V617F |  |  | 67088249 | 83.67% | 80X | 92 | 17 | 18.48% |  |  |
| Normal+ Mutant | *JAK2* p.V617F | 2.50% | 8.90% | 60412028 | 86.04% | 75X | 76 | 2 | 2.63% | 8.13% | 5.00% |
| Normal+ Mutant | J*AK2* p.V617F |  |  | 63764082 | 84.51% | 77X | 96 | 9 | 9.38% |  |  |
| Normal+ Mutant | *JAK2* p.V617F |  |  | 88321472 | 84.39% | 107X | 113 | 14 | 12.39% |  |  |
| Normal | N/A | 0.00% | / | 66350868 | 84.28% | 83X | 124 | 0 | 0.00% | / | / |
| Mutant | *BRAF* p.V600E | 100.00% | 68.70% | 50526950 | 85.42% | 62X | 41 | 25 | 61.00% | / | / |
| Normal+ Mutant | *BRAF* p.V600E | 60.00% | 36.60% | 67886975 | 94.00% | 85X | 113 | 39 | 34.51% | 33.01% | 2.74% |
| Normal+ Mutant | *BRAF* p.V600E |  |  | 69831047 | 94.00% | 87X | 75 | 26 | 34.67% |  |  |
| Normal+ Mutant | *BRAF* p.V600E |  |  | 71707603 | 94.00% | 89X | 67 | 20 | 29.85% |  |  |
| Normal+ Mutant | *BRAF* p.V600E | 30.00% | 18.30% | 67792013 | 94.00% | 85X | 73 | 12 | 16.44% | 17.44% | 7.12% |
| Normal+ Mutant | *BRAF* p.V600E |  |  | 70442911 | 94.00% | 88X | 46 | 5 | 10.87% |  |  |
| Normal+ Mutant | *BRAF* p.V600E |  |  | 69696199 | 94.00% | 87X | 68 | 17 | 25.00% |  |  |
| Normal+ Mutant | BRAF p.V600E | 12.50% | 7.50% | 76090968 | 84.75% | 91X | 58 | 5 | 8.62% | 7.73% | 2.70% |
| Normal+ Mutant | *BRAF* p.V600E |  |  | 68103384 | 85.10% | 82X | 81 | 8 | 9.88% |  |  |
| Normal+ Mutant | *BRAF* p.V600E |  |  | 70646482 | 84.98% | 85X | 85 | 4 | 4.71% |  |  |
| Normal+ Mutant | *BRAF* p.V600E | 5.00% | 3.30% | 59639856 | 84.47% | 72X | 62 | 1 | 1.61% | 2.08% | 1.00% |
| Normal+ Mutant | *BRAF* p.V600E |  |  | 74664018 | 84.66% | 90X | 71 | 1 | 1.41% |  |  |
| Normal+ Mutant | *BRAF* p.V600E |  |  | 72100183 | 84.86% | 87X | 62 | 2 | 3.23% |  |  |
| Normal+ Mutant | *BRAF* p.V600E | 2.50% | 1.60% | 67952667 | 84.42% | 81X | 47 | 2 | 4.26% | 2.62% | 1.42% |
| Normal+ Mutant | *BRAF* p.V600E |  |  | 75018062 | 84.75% | 90X | 59 | 1 | 1.69% |  |  |
| Normal+ Mutant | *BRAF* p.V600E |  |  | 70012696 | 83.91% | 84X | 52 | 1 | 1.92% |  |  |
| Normal | N/A | 0.00% | / | 66350868 | 84.28% | 83X | 53 | 0 | 0.00% | / | / |
| Mutant | *KRAS* p.G12V | 100.00% | 79.20% | 72073078 | 83.47% | 86X | 53 | 42 | 79.20% | / | / |
| Normal+ Mutant | *KRAS* p.G12V | 30.00% | 39.00% | 67886975 | 94.00% | 85X | 34 | 12 | 35.29% | 34.88% | 1.38% |
| Normal+ Mutant | *KRAS* p.G12V |  |  | 69831047 | 94.00% | 87X | 25 | 9 | 36.00% |  |  |
| Normal+ Mutant | *KRAS* p.G12V |  |  | 71707603 | 94.00% | 89X | 27 | 9 | 33.33% |  |  |
| Normal+ Mutant | *KRAS* p.G12V | 12.50% | 16.00% | 76090968 | 84.75% | 91X | 26 | 5 | 19.23% | 21.47% | 5.54% |
| Normal+ Mutant | *KRAS* p.G12V |  |  | 68103384 | 85.10% | 82X | 18 | 5 | 27.78% |  |  |
| Normal+ Mutant | *KRAS* p.G12V |  |  | 70646482 | 84.98% | 85X | 23 | 4 | 17.39% |  |  |
| Normal+ Mutant | *KRAS* p.G12V | 8.50% | 11.00% | 67792013 | 94.00% | 85X | 25 | 5 | 20.00% | 12.08% | 7.11% |
| Normal+ Mutant | *KRAS* p.G12V |  |  | 70442911 | 94.00% | 88X | 20 | 2 | 10.00% |  |  |
| Normal+ Mutant | *KRAS* p.G12V |  |  | 69696199 | 94.00% | 87X | 16 | 1 | 6.25% |  |  |
| Normal+ Mutant | *KRAS* p.G12V | 5.00% | 6.70% | 59639856 | 84.47% | 72X | 10 | 0 | 0.00% | 3.41% | 3.05% |
| Normal+ Mutant | *KRAS* p.G12V |  |  | 74664018 | 84.66% | 90X | 23 | 1 | 4.35% |  |  |
| Normal+ Mutant | *KRAS* p.G12V |  |  | 72100183 | 84.86% | 87X | 17 | 1 | 5.88% |  |  |
| Normal+ Mutant | *KRAS* p.G12V | 2.50% | 3.20% | 67952667 | 84.42% | 81X | 30 | 0 | 0.00% | 2.02% | 3.50% |
| Normal+ Mutant | *KRAS* p.G12V |  |  | 75018062 | 84.75% | 90X | 33 | 2 | 6.06% |  |  |
| Normal+ Mutant | K*RAS* p.G12V |  |  | 70012696 | 83.91% | 84X | 14 | 0 | 0.00% |  |  |
| Normal | N/A | 0.00% | / | 66350868 | 84.28% | 83X | 22 | 0 | 0.00% | / | / |
| Mutant | EGFRp.E746_A750del5 | 100.00% | 64.50% | 71033868 | 83.79% | 85X | 257 | 158 | 61.48% | / | / |
| Normal+ Mutant | *EGFR* p.E746_A750del5 | 12.50% | 30.60% | 76090968 | 84.75% | 91X | 179 | 51 | 28.49% | 28.21% | 4.47% |
| Normal+ Mutant | *EGFR* p.E746_A750del5 |  |  | 68103384 | 85.10% | 82X | 161 | 38 | 23.60% |  |  |
| Normal+ Mutant | *EGFR* p.E746_A750del5 |  |  | 70646482 | 84.98% | 85X | 166 | 54 | 32.53% |  |  |
| Normal+ Mutant | *EGFR* p.E746_A750del5 | 5.00% | 14.80% | 76090968 | 84.75% | 91X | 99 | 16 | 16.16% | 14.94% | 1.14% |
| Normal+ Mutant | *EGFR* p.E746_A750del5 |  |  | 68103384 | 85.10% | 82X | 108 | 15 | 13.89% |  |  |
| Normal+ Mutant | *EGFR* p.E746_A750del5 |  |  | 70646482 | 84.98% | 85X | 115 | 17 | 14.78% |  |  |
| Normal+ Mutant | *EGFR* p.E746_A750del5 | 2.50% | 8.70% | 67952667 | 84.42% | 81X | 108 | 11 | 10.19% | 8.32% | 1.64% |
| Normal+ Mutant | *EGFR* p.E746_A750del5 |  |  | 75018062 | 84.75% | 90X | 113 | 8 | 7.08% |  |  |
| Normal+ Mutant | *EGFR* p.E746_A750del5 |  |  | 70012696 | 83.91% | 84X | 104 | 8 | 7.69% |  |  |
| Normal | N/A | 0.00% | / | 66350868 | 84.28% | 83X | 52 | 0 | 0.00% | / | / |

Suppl. Table 10. Summary of Inter-assay and Intra-assay data

| Assay | Gene/Mutation | Replicate#/Run# | Avg. Total Reads | Avg. Captured Reads | Avg. Coverage | Avg. Strand Bias | Avg. Total Allele Count | Avg. ALT Allele Count | Avg. ALT Allele Fraction | Avg. ALT Allele Fraction SD | Avg. ALT Allele Fraction CV | Avg. Read Count Log2 Ratio | Avg. Read Count Log2 Ratio SD | Avg. Read Count Log2 Ratio CV |
| --- | --- | --- | --- | --- | --- | --- | --- | --- | --- | --- | --- | --- | --- | --- |
| Intra-assay | *JAK2* | PM12 | 70,245,738 | 86.19% | 87X | 60.94% | 78 | 14 | 17.66% | 5.41% | 0.31 | N/A | N/A | N/A |
|  |  | PM47 | 67,273,933 | 94.67% | 82X | 60.54% | 144 | 14 | 10.07% | 3.55% | 0.35 | N/A | N/A | N/A |
|  |  | PM49 | 69,786,731 | 95.00% | 85X | 56.63% | 130 | 25 | 19.21% | 0.84% | 0.04 | N/A | N/A | N/A |
|  | *EGFR* | PM13 | 67,344,006 | 85.88% | 82X | 50.79% | 71 | 5 | 7.69% | 3.65% | 0.47 | N/A | N/A | N/A |
|  |  | PM126 | 68,408,501 | 89.00% | 90X | 37.06% | 42 | 5 | 12.81% | 2.86% | 0.22 | N/A | N/A | N/A |
|  |  | PM23 | 73,170,470 | 86.72% | 93X | 26.56% | 115 | 16 | 14.02% | 1.72% | 0.12 | N/A | N/A | N/A |
|  | *HER2/neu* | PM98 | 66,617,687 | 89.67% | 88X | 49.89% | N/A | N/A | N/A | N/A | N/A | 0.67 | 0.049 | 0.07 |
|  |  | PM108 | 68,154,661 | 92.33% | 88X | 49.66% | N/A | N/A | N/A | N/A | N/A | 0.9202 | 0.0869 | 0.09 |
|  |  | PM112 | 67,535,754 | 90.10% | 89X | 49.88% | N/A | N/A | N/A | N/A | N/A | 0.5673 | 0.069 | 0.12 |
| Inter-assay | *JAK2* | PM12 | 67,168,563 | 84.29% | 81X | 60.85% | 99 | 16 | 15.74% | 7.84% | 0.50 | N/A | N/A | N/A |
|  |  | PM47 | 69,936,606 | 84.23% | 86X | 61.38% | 145 | 17 | 11.70% | 3.02% | 0.26 | N/A | N/A | N/A |
|  |  | PM49 | 65,975,143 | 84.17% | 81X | 57.34% | 118 | 24 | 20.62% | 2.32% | 0.11 | N/A | N/A | N/A |
|  | *EGFR* | PM13 | 61,396,854 | 87.40% | 76X | 51.13% | 52 | 6 | 10.01% | 3.31% | 0.33 | N/A | N/A | N/A |
|  |  | PM126 | 67,529,503 | 90.91% | 90X | 36.15% | 48 | 8 | 16.12% | 4.03% | 0.25 | N/A | N/A | N/A |
|  |  | PM127 | 79,032,885 | 92.63% | 104X | 37.78% | 99 | 8 | 8.06% | 3.51% | 0.44 | N/A | N/A | N/A |
|  | *HER2/neu* | PM98 | 66,341,622 | 90.23% | 87X | 49.81% | N/A | N/A | N/A | N/A | N/A | 0.7174 | 0.0481 | 0.07 |
|  |  | PM108 | 72,294,929 | 90.87% | 93X | 49.75% | N/A | N/A | N/A | N/A | N/A | 0.987 | 0.0256 | 0.03 |
|  |  | PM112 | 71,316,190 | 90.45% | 96X | 49.85% | N/A | N/A | N/A | N/A | N/A | 0.6218 | 0.0811 | 0.13 |

Suppl. Table 11. Allele frequency comparison study EXaCT-1 vs. AmpliSeq HotSpot assay

| Sample ID# | Sample type | Gene | HGVS mutation | AmpliSeq allele frequency | Type | COSMIC ID | AmpliSeq total coverage | AmpliSeq ALT allele frequency | EXaCT-1 total reads | EXaCT-1 VAF |
| --- | --- | --- | --- | --- | --- | --- | --- | --- | --- | --- |
| 1 | Frozen Tissue | *STK11* | c.833delG,p.C278_fs | 78 | del | NA | 510 | 398 | 30 | 80.0 |
| 2 | FFPE Tissue | *TP53* | c.817C>T, p.R273C | 92.2 | SNV | COSM10659 | 875 | 807 | 65 | 95.38 |
| 3 | FFPE Tissue | *TP53* | c.817C>T, p.R273C | 27.1 | SNV | COSM10659 | 631 | 171 | 120 | 28.33 |
| 4 | FFPE Tissue | *PTEN* | c.388C>G, p.R130G | 28.4 | SNV | COSM5219 | 1994 | 566 | 77 | 19.48 |
| 5 | FFPE Tissue | *PTEN* | c.388C>G, p.R130G | 86.7 | SNV | COSM5219 | 1993 | 1727 | 93 | 84.95 |
| 6 | FFPE Tissue | *TP53* | c.455C>T, p.P152L | 56.6 | SNV | COSM10790 | 1250 | 708 | 75 | 81.33 |
| 7 | Frozen Tissue | *KRAS* | c.35G>T p.G12V | 88.9 | SNV | COSM520 | 1989 | 1769 | 144 | 90.28 |
| 8 | FFPE Tissue | *PIK3CA* | c.3140A>T p.H1047L | 78 | SNV | COSM776 | 1995 | 1557 | 150 | 72.67 |
| 9 | FFPE Tissue | *PIK3CA* | c.3140A>T p.H1047L | 60.8 | SNV | COSM94987 | 1994 | 1212 | 125 | 48.8 |
| 10 | Frozen Tissue | *TP53* | c.536A>G p.H179R | 81.4 | SNV | COSM10889 | 1986 | 1616 | 70 | 82.86 |
| 11 | FFPE Tissue | *IDH1* | c.395G>T p.R132L | 19.4 | SNV | COSM28750 | 1688 | 327 | 56 | 30.36 |
| 12 | Frozen Tissue | *HRAS* | c.34G>A, p.G12S | 71.8 | SNV | COSM 480 | 1998 |  | 155 | 69 |
| 13 | FFPE Tissue | *TP53* | c.538G>T, p.E180* | 28.7 | SNV | COSM43597 | 1347 |  | 206 | 24.3 |
| 14 | FFPE Tissue | *TP53* | c.584T>C, p.I195T | 60.7 | SNV | COSM11089 | 1014 |  | 164 | 90.85 |
| 15 | Frozen Tissue | *IDH1* | c.395G>A p.R132H | 32.4 | SNV | COSM28746 | 1998 | 647 | 37 | 35.14 |
| 16 | Frozen Tissue | *TP53* | c.524G>A p.R175H | 91.8 | SNV | COSM10648 | 1995 | 1831 | 113 | 90.27 |
| 17 | Frozen Tissue | *PIK3CA* | c.223 C>G, p.Q75E | 36.2 | SNV | COSM39166 | 1137 |  | 79 | 37.97 |
| 18 | Frozen Tissue | *TP53* | c.701>A>G, p.Y234C | 61.6 | SNV | COSM10725 | 1900 |  | 171 | 52.6 |
| 19 | Frozen Tissue | *TP53* | c.839G>C, p.R280T | 88.57 | SNV | COSM10724 | 691 |  | 105 | 74 |
| 20 | FFPE Tissue | *CTNNB1* | c.110C>T, p.S37F | 45.99 | SNV | COSM5662 | 411 |  | 215 | 31 |
| 21 | FFPE Tissue | *PTEN* | c.517C>T, p.R173C | 43.84 | SNV | COSM5089 | 828 |  | 36 | 39 |
| 22 | Frozen Tissue | *TP53* | c.536A>G, p.H179R | 82.47 | SNV | COSM10889 | 1997 |  | 70 | 83 |
| 23 | FFPE Tissue | *TP53* | c.711G>T, p.M237I | 55.21 | SNV | COSM11063 | 826 |  | 256 | 48 |
| 24 | Frozen Tissue | *KRAS* | c.35G>T, p.G12V | 35.79 | SNV | COSM520 | 908 |  | 26 | 46 |
| 25 | Frozen Tissue | *TP53* | c.393_395delCAA, p.N131del | 28.83 | del | COSM44212 | 1984 |  | 88 | 31 |
| 26 | Frozen Tissue | *EGFR* | c.1793G>T, p.G598V | 94.99 | SNV | COSM21690 | 1994 |  | 1036 | 94 |

*Not reported on CHP because this variant has no Cosmic ID; Qlty:6055, VAF:0.78, RD:510, SB:0.5). Seen twice on two separate CHP runs. Visible in CHP VCF files.

Suppl. Table 12. EXaCT-1 allele frequency calls using Horizon reference FFPE DNA

| Mutation | Genomic coordinate (hg19) | Expected AF | Average (N=2) | |
| --- | --- | --- | --- | --- |
|  |  |  | Observed AF | Coverage |
| *EGFR* G719S | chr7:55241707 | 26.0% | 13% | 98 |
| *KRAS* G13D | chr12:25398281 | 16.5% | 15% | 50.5 |
| *PI3KCA* H1047R | chr3:178952085 | 15.5% | 16% | 165.5 |
| *NRAS* Q61K | chr1:115256530 | 12.0% | 14% | 160.5 |
| *PI3KCA* E545K | chr3:178936091 | 10.0% | 7% | 55 |
| *BRAF* V600E | chr7:140453136 | 9.5% | 12% | 90.5 |
| *c-Kit* D816V | chr4:55599321 | 9.0% | 14% | 58 |
| *KRAS* G12D | chr12:25398284 | 5.5% | 10% | 50.5 |
| *EGFR L858R* | chr7:55259515 | 2.5% | 3% | 117 |
| *EGFR* ΔE746-A750 (exon 19) | chr7:55242461-55242486 | 1.5% | 0% | 254 |
| *EGFR* T790M | chr7 55249071 | 1.0% | 0% | 219.5 |

**Supplementary Methods**

**Samples and performance evaluation controls**

To address the clinical utility of EXaCT-1, we selected a diverse representation of solid tumors and hematological cancers (blood and bone marrow) submitted to NewYork Presbyterian Hospital/Weill Cornell Medical College (NYPH/WCMC) Clinical Laboratories (New York, NY) for routine clinical testing. For the purposes of developing a New York State Department of Health submission, the sample set was composed of 45 archival formalin-fixed paraffin embedded (FFPE) and 12 fresh/frozen primary tumors with known mutations, comprising were used for the study. For solid tumors, hematoxylin and eosin (H&E) stained slides of both FFPE and frozen tissue blocks were reviewed by the study pathologists for tumor purity assessment and selection of high-density areas for manual macrodissection and DNA extraction. The extracted genomic DNA was stored at -20°C until exome sequencing analysis was performed. All samples had undergone molecular testing by NYPH Clinical Laboratories for one of the five disease relevant mutations prior to validation. The study was approved by the IRB Committee at Weill Cornell Medical College (IRB protocols #1305013903, #1007011157, #1210013164A005), and informed consent was obtained.

The analytical characteristics of the new HaloPlex WES assay were established using HapMap DNA NA12878 and NA19240, thoroughly characterized by a number of genotyping methods ^1^, from Coriell (Camden, NJ) and quantitative multiplex DNA formalin-fixed reference material (HD-C750) (Horizon Diagnostics, Cambridge, UK), 45 normal peripheral blood DNA specimens and 57 patients’ samples, compromising tumors from different tissue and cancer types as indicated above. For accuracy, HapMap DNA NA12878 sequencing results were compared against a whole genome reference material ^1^. This benchmap dataset is a high-confidence, SNP/indel and homozygous reference genotypes, comprised of genotype calls for NA12878 integrated and arbitrated between 14 data sets from five sequencing technologies, seven read mappers and three variant callers ^1^.

The overall assay accuracy was calculated as the number of genotypes that agree vs. total number of genotypes called using the following definitions:

True positives (TP): Number of sequence variations identified by exome sequence and are reported in the database. True negatives (TN): Number of sequence variations absent from the dataset and by exome sequencing. False positives (FP): Number of sequence variations found by exome sequencing but not reported in the HapMap dataset ^1^. False negatives (FN): Number of sequence variations reported in the HapMap dataset, but not found by exome sequencing.

The overall assay technical sensitivity and specificity were determined using the following formulas:
Sensitivity = Number of true positive/(number of true positive + number of false negative),

Specificity = Number of true negative/(number of true negative + number of false positive).

Positive predictive value (PPV) = Number of true positive/(number of true positive + number of false negative).

Analytical sensitivity for specific mutations was determined using cells lines obtained from ATCC (BT474, HEL92.1.7, SK-CO-1, COLO-205) (ATCC (Manassas, VA), and the BRC RIKEN BioResource Center (PC-9) (Tsukuba, Japan), respectively. Detailed characterization of these cells in provided in the Results section. For determining the analytical sensitivity of the assay, DNA extracted from the cell lines harboring the validated mutations was further diluted with HapMap DNA to achieve the desired variant allele frequency as measured using the Ion Torrent PGM System (Life Technologies, Grand Island, NY) and digital PCR (Bio-Rad Laboratories, Hercules, CA), and analyzed in triplicates. The precision (within run), reproducibility (between run) and barcode crosstalk were determined, using three positive patient samples containing variants near the stated sensitivity of the assay (10%, as noted in the Results section) and measured on two different days using different barcodes. Crosstalk (PCR contamination occurring when preparing libraries from two or more genomic DNA samples side by side, each with a unique index) between indexed samples was also assessed throughout the HaloPlex exome using HapMap genomic DNA samples enriched for exome targets as described below, and pooled in the same lane for multiplex sequencing on the instrument.

**DNA extraction and quality assessment**

Genomic DNA was extracted from macrodissected FFPE tumor and/or cored frozen, OCT-embedded tumor, and peripheral blood lymphocytes (PBLs) using the Promega Maxwell 16 MDx according to the manufacturer’s instructions (Promega, Madison, WI). Overall DNA quality was assessed using NanoDrop spectrophotometer (Thermo Fisher Scientific, Waltham, MA) and Qubit 2.0 fluorometer (Life Technologies). FFPE DNA quality was further assessed using a multiplex PCR-based qualification assay as recommended by Agilent Technologies. Briefly, each FFPE DNA sample is used as template for the PCR amplification of two independent *GAPDH* amplicons. The yield of amplicons from the FFPE DNA template is measured with Bioanalyzer (Agilent Technologies, Santa Clara, CA) and compared to the yield of amplicons from an intact reference DNA template. The resulting sample-to-reference yield ratio is then used as a quantitative indicator of DNA integrity, for predicting sample performance in HaloPlex Exome target enrichment. Based on the apparent integrity of the FFPE DNA samples, DNA was categorized into three groups: A (yield ratio >0.2), B (yield ratio 0.05~0.2) and C (yield ratio <0.05), and the input DNA amount for HaloPlex library preparation was adjusted according to the category. Requirements for acceptable DNA included total DNA amount >225 ng for FF HMW DNA, >500ng for high-quality (category A) FFPE DNA and >1000ng for low-quality (category B) FFPE DNA (by Qubit), and absorbance ration A260/A280 (range 1.8~2.0), A260/A230 (>2.0).

**Next generation sequencing using HaloPlex Illumina HiSeq**

For library selection studies, exome captured library preparations were performed following the manufacturers’ protocol (Agilent Technologies, Illumina and NimbleGen) and the Illumina HiSeq 2500 (San Diego, CA), according to the manufacturer’s instructions. WES clinical validation was performed using the Agilent HaloPlex Library. Briefly, 225 ng of genomic DNA (500ng and 1000ng for category A and B FFPE DNA, respectively) were digested using restriction enzymes, purified and assessed for quality using the Agilent 2100 Bioanalyzer. The resulting DNA was hybridized to HaloPlex probes coupled to Illumina adaptors and sample barcodes followed by solid phase capture, ligation to form circular structures and amplification by PCR, followed by purification using the Agencourt AMPure XP beads (Beckman Coulter, Brea, CA). The amplified enriched library was then loaded onto a flow cell for on-board cluster generation and sequencing on an Illumina HiSeq 2500 system (100 bp paired-end reads) in rapid mode (4 samples/lane).

**Confirmatory studies**

All DNA samples of 57 patients included as positive controls in this study were previously analyzed by conventional PCR-based methods. Briefly, DNA samples harboring *KRAS* or *BRAF* mutations were amplified using either exon 2 or exon 15-specific primers, and directly sequenced using an ABI 3130 Genetic Analyzer (ABI-Life Technologies, Grand Island, NY). *EGFR* del19 mutations were tested by real-time PCR, using the EGFR RGQ PCR Kit and the Rotor-Gene Q Instrument according to the manufacturer’s instructions (Qiagen).  *JAK2* mutation was confirmed using an amplification refractory mutation system (ARMS) assay for selectively amplifying the mutated allele. *HER2/Neu* confirmation was performed by fluorescence in situ hybridization (FISH) dual probes using a specific *HER2/Neu* labeled red for detecting human *HER2/neu* at 17q12 and chromosome 17 centromere probe (alpha-satellites DNA D17Z1 ,green) (GeneMed Biotechnologies, Inc., San Francisco, CA). At least 100 nuclei were evaluated per tissue section using a fluorescence microscope (Olympus BX51; Olympus Optical, Tokyo, Japan).

For the analytical sensitivity studies, variant calls for the dilution studies were confirmed using the Ion AmpliSeq Cancer Panel hotspot v2 (Ion Torrent, Life Technologies, Grand Island, NY), according to a CLIA-approved laboratory procedure. Briefly, Genomic DNA is amplified, enriched and sequenced using next generation bidirectional sequencing technology on the Ion Torrent Personal Genome Machine (Life Technologies). The Ion Ampliseq Cancer Panel hotspot v2 targeted gene panel is designed to detect 2,800 mutations/variants in 207 amplicons from 50 key cancer genes. Quality control measures are implemented at multiple levels of the sequencing process. Annotation of variants is performed using the Torrent Suite Software v 4.2 (Life Technologies). *HER2/neu* amplification was confirmed by droplet digital PCR (ddPCR) using the QX200 Droplet Digital PCR system (Bio-Rad Laboratories), according to the manufacturer’s instructions. ddPCR data were collected and analyzed with QuantaLife® software V2.0 (Bio-Rad). An invariant single copy per haplotype gene, RPP30, was assayed on the HEX channel, and employed as internal reference gene.

**Data processing and quality control**

NGS data are analyzed with a custom bioinformatic pipeline. PE 100bp reads from the Illumina sequencers are demultiplexed using Casava 1.8. Company-provided adapter sequences are trimmed using Trimmomatic^2^. The quality of the raw reads is assessed by FastQC, a software suite developed by Babraham Bioinformatics (<http://www.bioinformatics.babraham.ac.uk/projects/fastqc/>). Reads are mapped against the GRC37/hg19 human reference genome using BWA v6.2 with default parameters except –e 50 (to enable long indel detection). GATK v2.3.9 with default parameters is applied for a local realignment and base quality score recalibration of the mapped reads. This results into what we term a “clean” BAM file, ready for QC and variant calling. The alignment quality of the aligned BAM files is obtained by calculating several metrics related to the average coverage at 10x or more and capture rate by calculating the number of aligned reads present within a capture region in the HaloPlex WES assay. Coverage (capture rate) was obtained by calculating the percent of mapped reads found overlapping any capture region in the assay and the total number of mapped reads of any given sample.

Point mutations and indels are detected using an analytics pipeline designed to not miss important mutations. The pipeline uses several tools and workflows to perform analysis of tumor samples with or without matched germline samples. For samples without germline matched controls, we run the GATK SNV caller with default parameters, PINDEL for long indels as well as COSMIC-based mpileup tool that interrogates specific positions known to be recurrently (>1) mutated in tumors according to the COSMIC database (http://cancer.sanger.ac.uk/cosmic/). For samples with germline controls, we run the above analysis plus a de novo somatic SNV caller, SNVseeqer, where mutations found at positions reported in dbSNP are filtered out ^3^ and (2) GATK somatic indel. When a germline sample is present, germline variants are eliminated from the somatic variant list using an FDR corrected Fisher exact test. Furthermore, mutations are filtered by variant allele frequency (VAF) where the mutation has to be present in the tumor with a VAF >25% and in the matched control its presence must have a VAF <1%. To control for known disease-associated loci that would be inappropriately filtered out, we have implemented a mutation rescue mechanism for mutational hotspots according to the COSMIC database of somatic cancer mutations (the Sanger Institute). Mutation hotspots are defined mutations detected in at least 2 patients. This mutation rescue approach is based on “samtools mpileup” and reports mutations that have a VAF >10% in the tumor and <1% in the control. Importantly, this mutation rescue analysis does not use any dbSNP filtering and will therefore, rescue and detect the type of important disease-associated variants present in dbSNP that may be filtered out. Mutations must be covered by at least five aligned reads in both the tumor and control sample. The output of these tools is merged into a germline and somatic variant lists. Point mutations are kept if they are located in coding sequences and caused an amino acid change determined by SNVseeqer. Indels in coding sequences are all kept, irrespective of whether they induce frameshift or not. We also record all filtered variants, i.e. those not meeting the criteria just mentioned, for the sign-out review process. An add-on document allows the pathologist to review both reported and filtered ones and “over-ride” the automated pipeline calls if he or she deemed that it is important to report a variant even if suboptimal. If total allele count is less than 30 for tumor and matched DNA control (we round it up from the 28 of the power calculation to simplify the reporting process) and the mutation is either in a known COSMIC hotspot or a clinical relevant mutation, the variant is included in the clinical report with a “warning” requesting orthogonal validation, e.g. with deep-amplicon sequencing (AmpliSeq). All other variants (i.e. non in COSMIC or in clinical relevant genes) not meeting the 30X coverage are included in the add-on in the filtered section for molecular pathologists’ review. If deemed to be relevant for the clinical case, those suboptimal variants can be included in the report with a warning.

For somatic copy number alterations, the number of aligned reads for captured region in the Agilent HaloPlex Whole Exome Kit was calculated in both the tumor and matched control sample (if present). If absent, we used read counts from pre-specified ten germline controls. Capture regions with a total coverage <100 reads are filtered out, read counts are normalized by the total number of reads aligned, and the ratio of the normalized read counts in the tumor sample and the normalized read count in the control sample is calculated. These capture regions are then ordered karyotypically and sorted by genomic coordinates to segment capture regions according to the log2 value of the ratio of normalized read counts of the tumor sample and control sample in a biologically meaningful way. The normalized ratios of these bins are segmented using the Circular Binary Segmentation algorithm implemented in the R package DNAcopy ^4^. The algorithm outputs segments where every capture region found within these segments is represented by the same log2 value. This log2 value indicates whether the segment has DNA copy number gain (amplification) or DNA copy number loss (deletion)^5^. A negative log2 suggests a segment was lost and a positive value would suggest a segment is gained. To provide a better level of granularity we define the following: segments with a log2 value > 1 and between 0.5 and 1 are considered amplified and areas of copy number gains, respectively. Log2 values < -1 and between -0.5 and -1 are considered deletions and regions of copy number loss, respectively. These thresholds were determined based on valleys in the distribution of log2 ratios (post-segmentation) in > 100 WES runs. To consider the effect of tumor ploidy and neoplastic content in the specimen, we also employ a couple of tools to estimate a corrected log2 value: 1. CLONET, a computational tool which employs a local optimization strategy to estimate tumor purity, ploidy and corrects the log2 ratio accordingly ^6^ and 2. A simple, global correction of the log2 values, based on tumor purity and ploidy determined by CLONET^7^. Given the limitations of WES in detecting CNAs in regions that have sparse exome coverage, as noted by Magi et al.^25^, we include this information as well as log2 plots showing the actual data for the clinically relevant and cancer genes in the add-on report thus enabling the molecular pathologist to review the calls and “over-ride” them, i.e. either include a filtered one back into the report or remove some borderline calls. Using a custom script, we then take the segments called by the algorithm and annotate these segments by RefSeq genes whose transcription start and end sites overlap with the genomic coordinates assigned to these segments. We also annotate if the segments are focal (50 genes or less) or large scale (>50 genes).

Clinically relevant CNAs only include amplifications or deep deletions, (i.e. “homozygous” loss) where more stringent thresholds are considered. Pathologists, when reviewing a case, can also verify the evidence of the WES data via the add-on document and choose to confirm the variant with orthogonal methods. All revisions to the automated pipeline calls are recorded.

The alignment and analysis of the exome data is processed on a Sun Grid Engine computing cluster where each run uses 8 CPU cores and 16Gb of memory. Under these conditions an exome on average takes ~8-10 hours to align and analyze for genomic alterations. Quality control of the process was assessed in three principal phases: 1) The quality of the raw reads (FastQC software), 2) The quality of the alignment (by average coverage and capture rates), 3) The quality of the samples. Short reads are stored for at least 5 years in agreement with NYS-DOH guidelines.

**Report generation**

Mutations detected by the analytical pipeline are consolidated into a set of flat files. These files are used as input to a custom program that generates a report in Portable Document Format (PDF). The report includes mutation list and interpretation for actionable events (drawn from a custom-made database at http://pmkb.weill.cornell.edu), sample identifier, diagnosis and histology, date of sample collection. It also reports number of reads and average coverage. Finally, a report add-on includes copy number alteration profiles as well as link out to Integrated Genome Viewer (IGV) that enables the user to browse the sequencing data supporting the call. The IGV link out will automatically direct IGV to the locus of the variant and display both the log2 ratio of the segment, and the tumor and normal reads.

**References**

1. Zook, J.M. et al. Integrating human sequence data sets provides a resource of benchmark SNP and indel genotype calls. *Nat Biotechnol* **32**, 246-251 (2014).

2. Bolger, A.M., Lohse, M. & Usadel, B. Trimmomatic: a flexible trimmer for Illumina sequence data. *Bioinformatics* **30**, 2114-2120 (2014).

3. Jiang, Y. et al. Deep sequencing reveals clonal evolution patterns and mutation events associated with relapse in B-cell lymphomas. *Genome Biol* **15**, 432 (2014).

4. Olshen, A.B., Venkatraman, E.S., Lucito, R. & Wigler, M. Circular binary segmentation for the analysis of array-based DNA copy number data. *Biostatistics* **5**, 557-572 (2004).

5. Magi, A. et al. EXCAVATOR: detecting copy number variants from whole-exome sequencing data. *Genome Biol* **14**, R120 (2013).

6. Prandi, D. et al. Unraveling the clonal hierarchy of somatic genomic aberrations. *Genome Biol* **15**, 439 (2014).

7. Baca, S.C. et al. Punctuated evolution of prostate cancer genomes. *Cell* **153**, 666-677 (2013).
