# Supplementary material for: Development and validation of a whole-exome sequencing test for simultaneous detection of point mutations, indels and copy-number alterations for precision cancer care
Source: NPJ Genom Med. 2016 Jul 20;1:16019–. doi: 10.1038/npjgenmed.2016.19 (PMC5539963; doi:10.1038/npjgenmed.2016.19)
Supplement: Supplementary File 1 [file npjgenmed201619-s2.pdf]

Whole Exome Sequencing Test for Cancer - EXaCT-1

CLINICAL INFORMATION

**Patient ID:**	PMTEST	**Specimen IDs (case/control)**	
**Requesting physician:**		**Sample type (case/control):**	/
**Tumor Type:**		**Sample collected (case/control):**	( ) / ()
**Primary site:**		**Sample received (case/control):**	() / ()
**Tissue Tested:**		**Neoplastic content:**	(See Notes)

CASE IMAGES

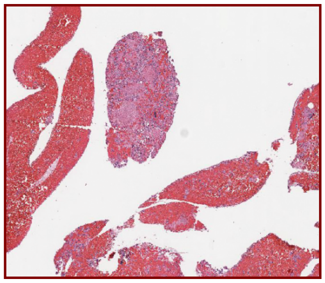

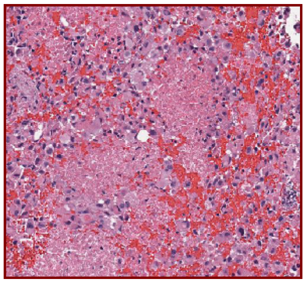

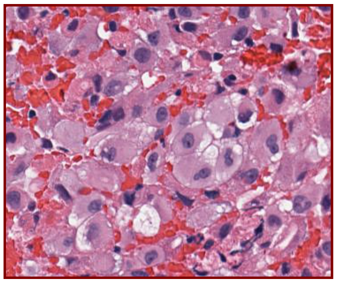

RESULTS

GENOMIC ALTERATIONS: Summary

Somatic alterations in clinically relevant genes

A set of 54 clinically relevant genes was investigated. 1 alteration was found in these genes (listed below).

Other somatic alterations in cancer genes

A set of 574 known cancer genes was investigated. 8 alterations in these cancer associated genes were found (listed below).

Somatic alterations of unknown significance

32 gene(s) with point mutations or indels and 17 copy number alteration(s) were found (listed below).

Clinically relevant genomic alterations

These alterations occur in genes that are deemed clinically relevant because: they are targets of drugs, they confer resistance or susceptibility to treatment, or for other clinically relevant reasons (see Appendix).

Copy number alterations

Gene name (location)	CNA type	FDA approved drugs with indication (if any)	Interpretation
MET 7:115,594,665-117,120,173 q31.2	focal amplification	-	MET amplification may be associated with sensitivity to MET inhibitors. MET amplification is associated with resistance to EGFR inhibitors in EGFR mutated lung cancer. MET alterations are commonly associated with papillary renal-cell carcinoma and it is very rare in clear cell renal cell carcinoma.

Genomic coordinates are based on human reference GRC37/hg19. See Appendix for all definitions.

Notes

The status of alterations in gene(s) **FLT3, KRAS** is **indeterminate** because the coverage was below the optimal levels of this method (<30 reads). If this finding will alter clinical management, then confirmation by an independent testing method should be performed.

* MET alterations are commonly associated with papillary renal-cell carcinoma; however, this tumor is positive for MET amplification even though it is very rare in ccRCC.

* The tumor demonstrates increased chromosome 7 copy number which encodes MET but also involves other genes.

Other genomic alterations in cancer genes

These alterations occur in genes that are cancer associated (see Appendix).

Somatic mutations and indels

Gene name	Classification	Reference Allele	Tumor Allele 1	Tumor Allele 2	AA change	Tumor (Normal) read depth	Tumor VAF
WT1 11:32,414,251	missense	G	G	A	p.R434C	53 (103)	11.32%

AA: amino-acid; VAF: variant allele frequency; Genomic coordinates are based on human reference GRC37/hg19 and are 1-based.
Alterations with VAF < 10%, coverage < 30x or < 5 mutated reads are below optimal detection conditions and should be considered as putative.

Copy number alterations

Altered region	Classification of CNA	Number of cancer genes	Cancer genes
14:102,551,699-107,283,201 q32.31-q32.33	broad copy number loss	1	AKT1
7:117,149,140-124,477,264 q31.2-q31.33	focal copy number gain; partial	1	POT1
7:128,040,906-135,661,810 q32.1-q33	broad copy number gain	1	SMO
7:2,255,575-4,007,006 p22.2-p22.3	focal copy number gain	1	CARD11
7:44,270,627-66,240,289 p11.1-q11.21	broad copy number gain	2	EGFR, IKZF1
7:5,104,785-40,899,994 p14.1-p22.1	broad copy number gain	8	HOXA11, RAC1, HOXA13, HOXA9, JAZF1, PMS2, HNRNPA2B1, ETV1
7:75,695,705-99,227,226 q11.23-q22.1	broad copy number gain	2	CDK6, AKAP9

Genomic coordinates are based on human reference GRC37/hg19. See Appendix for all definitions.

Genomic alterations of unknown significance

These alterations are not known to have any effect on the disease, but are here reported in the event that in the future progress in scientific knowledge could determine their role (see Appendix).

Somatic mutations and indels

Gene name	Classification	Reference Allele	Tumor Allele 1	Tumor Allele 2	AA change	Tumor (Normal) read depth	Tumor VAF
WDR24 16:735,965	missense	C	C	G	p.G493R	80 (126)	46.2%
NOS2 17:26,101,453	missense	G	G	T	p.H436N	34 (113)	32.4%
MIR138-1 3:44,155,717	frameshift insertion	-	+G	-	p.W6_fs	18 (28)	33.3%
MAP4K2 11:64,566,281	missense	T	T	A	p.K361M	54 (56)	51.9%
KIAA1683 19:18,376,380	missense	A	A	G	p.L657P	98 (112)	33.7%
DST 6:56,464,898	missense	A	A	G	p.V1769A	131 (109)	42.7%
VPS37A 8:17,137,801	missense	G	G	T	p.D299Y	23 (35)	60.9%
IGFN1 1:201,180,317	missense	A	A	G	p.E2099G	146 (108)	28.1%
MYO19 17:34,852,241	missense	T	T	C	p.K923E	27 (51)	40.7%
HECTD1 14:31,598,111	missense	G	G	A	p.P1489L	147 (210)	42.2%
HNRNPA1 12:54,675,583	missense	T	T	C	p.M46T	94 (87)	26.6%
BCKDHA 19:41,916,704	missense	C	C	A	p.P91T	74 (85)	40.5%
MYO9A 15:72,144,533	frameshift deletion	AAGCC	-	AAGCC	p.R2137_fs	21 (33)	52.4%
TTF2 1:117,618,165	missense	A	A	C	p.H320P	203 (124)	25.6%
TIMD4 5:156,376,689	missense	C	C	T	p.A245T	235 (185)	31.1%
MESDC2 15:81,282,126	missense	C	C	T	p.A3T	40 (49)	67.5%
FAM136A 2:70,524,488	missense	T	T	C	p.D117G	43 (124)	37.2%
ANKRD10 13:111,567,160	missense	A	A	T	p.L41Q	45 (61)	48.9%
MORF4L1 15:79,170,574	missense	T	T	A	p.F20L	38 (67)	39.5%

Gene name	Classification	Reference Allele	Tumor Allele 1	Tumor Allele 2	AA change	Tumor (Normal) read depth	Tumor VAF
DNAH8<br>6:38,709,496	missense	G	G	C	p.E376Q	89 (60)	29.2%
SLC35F6<br>2:26,997,987	missense	T	T	G	p.S76A	52 (62)	36.5%
UBE2J1<br>6:90,045,086	missense	A	A	G	p.S165P	54 (43)	44.4%
FLG2<br>1:152,327,773	missense	G	G	T	p.S830Y	176 (281)	42.6%
MAGEC3<br>X:140,985,485	missense	T	T	A	p.F600Y	132 (109)	65.2%
C11orf87<br>11:109,294,576	missense	T	T	C	p.C73R	69 (146)	59.4%
PKHD1L1<br>8:110,471,831	nonsense	C	C	T	p.Q2338X	64 (67)	39.1%
TKT<br>3:53,267,183	missense	C	C	T	p.R254Q	32 (49)	31.2%
DNAH9<br>17:11,535,915	missense	T	T	G	p.N510K	62 (93)	40.3%
VCPIP1<br>8:67,577,498	missense	T	T	C	p.R566G	17 (27)	58.8%
TMEM139<br>7:142,983,780	missense	C	C	T	p.A170V	142 (117)	28.9%
TAS2R7<br>12:10,954,392	missense	T	T	A	p.M260L	281 (208)	33.5%
FAM120B<br>6:170,627,739	missense	G	G	C	p.A444P	36 (52)	47.2%

AA: amino-acid; VAF: variant allele frequency; Genomic coordinates are based on human reference GRC37/hg19 and are 1-based.
Alterations with VAF < 10%, coverage < 30x or < 5 mutated reads are below optimal detection conditions and should be considered as putative.

Copy number alterations

Altered region	Classification of CNA	Number of genes	Gene names (if less than 15)
11:126,135,915-126,136,735<br>q24.2	focal deletion; partial	1	SRPR
14:102,452,560-102,551,234<br>q32.31	focal deletion	2	HSP90AA1; DYNC1H1
14:102,551,699-107,283,201<br>q32.31-q32.33	broad copy number loss	64	too many to show
17:78,263,545-78,367,219<br>q25.3	focal copy number loss	2	LOC100294362; RNF213
3:121,634,499-121,658,267<br>q13.33	focal copy number loss; partial	1	SLC15A2

Altered region	Classification of CNA	Number of genes	Gene names (if less than 15)
7:115,594,665-117,120,173 q31.2	focal amplification	13	TFEC; CAV2; ST7; CFTR; WNT2; TES; ASZ1; ST7-AS1; CAPZA2; ST7-AS2; ST7-OT3; CAV1; ST7-OT4
7:117,149,140-124,477,264 q31.2-q31.33	focal copy number gain	29	too many to show
7:128,040,906-135,661,810 q32.1-q33	broad copy number gain	71	too many to show
7:148,823,262-158,937,225 q36.1-q36.3	broad copy number gain	98	too many to show
7:2,255,575-4,007,006 p22.2-p22.3	focal copy number gain	14	NUDT1; SNX8; MAD1L1; FTSJ2; AMZ1; MIR4648; TTYH3; BRAT1; GNA12; CHST12; LFNG; IQCE; SDK1; EIF3B
7:44,270,627-66,240,289 p11.1-q11.21	broad copy number gain	106	too many to show
7:5,104,785-40,899,994 p14.1-p22.1	broad copy number gain	217	too many to show
7:75,695,705-99,227,226 q11.23-q22.1	broad copy number gain	136	too many to show
7:99,261,652-115,590,963 q22.1-q31.2	broad copy number gain	159	too many to show
8:142,500,294-143,310,890 q24.3	focal copy number loss	4	MROH5; MIR4472-1; TSNARE1; LINC00051
X:200,916-2,688,609 p22.33	focal copy number loss	2	LINC00102; XG
Y:2,655,335-23,763,771 p11.1-q11.223	broad deletion	74	too many to show

Genomic coordinates are based on human reference GRC37/hg19. See Appendix for all definitions.

Method

Genomic DNA was extracted from macrodissected formalin-fixed paraffin-embedded (FFPE) tumor, or cored frozen, OCT-embedded tumor and peripheral blood lymphocytes of the patient's specimens using the Promega Maxwell 16 MDx. Estimation of tumor content is based on analysis of the sequencing data using CLONET version 1.0 [1]. Sequencing was performed using Illumina HiSeq 2500 (2x100bp). A total of 21,522 genes were analyzed with an average coverage of 80x (88x) using Agilent HaloPlex. 60,383,384 (69,325,152) short reads were aligned to GRC37/hg19 reference using BWA [2] and processed accordingly to Whole Exome Sequencing Test for Cancer - ExaCT1 - pipeline v0.9. The capture efficiency was 91.95% (86.46%).

NB: numbers in parentheses refer to the corresponding patient's control sample.

1. Prandi D. et al. Unraveling the clonal hierarchy of somatic genomic aberrations. Genome Biol 2014;15:439. doi:10.1186/s13059-014-0439-6.
2. Li, Heng, and Durbin Richard. Fast and Accurate Long-read Alignment with Burrows-Wheeler Transform. Bioinformatics 2010;26(5)(March 1):589–595. doi:10.1093/bioinformatics/btp698

Limitations of the assay

1. The analytical sensitivity of the assay is approximately 10% (with a minimum neoplastic content of 20%), thus, mutations present in a lower percentage of cells may not be identified by this assay. Use of insufficient DNA template can result in low PCR product yields, and sequence signals may fall below detection limits.

2. The human exome is not captured in its entirety, because not all human genes are identified and some genes may not be amendable to capture. Pathogenic mutations located in genes that are non-coding, have corresponding pseudogenes, contain repetitive or high GC-region will not be detected. Information about low coverage regions by this test is provided on our website at: http://trp.med.cornell.edu/IPMWES/HaloPlex_low_coverage_region.xlsx
3. Medium to large indels above 30% of the read length (>60bp) may not be detected due to the short (~200 bp) Illumina reads.
4. The ability of this assay to identify copy number alterations is reduced in cases with low tumor percentage (e.g., less than 50% tumor); in such cases, copy number alteration data (including the apparent absence of copy number alterations) should be interpreted with caution since the findings may not be representative.
5. Any actionable sequence variant detected by this test (or lack of thereof) requires confirmation by an independent testing method before altering clinical management based on the findings.
6. Some regions of genes cannot be fully evaluated for mutations or indels because of lack of sufficient coverage.

Disclaimer

The products and procedures used in this evaluation are for experimental or research use only. Any findings that will be used to alter clinical management require confirmation by an independent method.
This method has not been cleared by the FDA. The analytical performance characteristics have been determined by the Englander Institute for Precision Medicine/New York Hospital Laboratories.
The lack of a given genetic alteration in this report does not necessarily indicate the absence of the alteration in the tumor since technical aspects of the assay, including inadequate coverage of some genes, limit the data that can be acquired in some genetic regions.
Alterations that occur in the germline are not reported and borderline copy number alterations may not be reported depending on the quality of the copy number signal(s) and background.

Appendix

Clinically relevant genes: These genes are deemed clinically relevant because: they are targets of drugs, they confer resistance or susceptibility to treatment, or for other clinically relevant reasons. As the scientific knowledge increases, this list will be updated accordingly. A total of 141 alterations in 54 genes are considered in this report.

Somatic alterations of unknown significance in cancer genes or in other genes: These genes may not be related to the disease. Current scientific knowledge cannot determine the impact of these alterations on the disease. These genes are included herein in the event they become clinically relevant as our knowledge increases. Specifically, this report considers a total of 574 cancer genes that are listed in the section 'Other genomic alterations in cancer genes' if alterations are found.

Alterations are not listed in ranked order: The order of the alterations reported as clinically relevant or of unknown significance is **not** associated with predicted effect on tumor development, progression, or resistance to treatment.

Copy number alterations: These alterations involve duplication of loss of genomic material. The following definitions are used:

- *Focal*: A genomic alteration in a region involving less than 50 genes.
- *Broad*: A genomic alteration in a region involving 50 genes or more.
- *Copy Number Gain*: A genomic alteration leading to increased copies in tumor relative to the control sample (log2 ratio between 0.5 and 1.0).
- *Copy Number Loss*: A genomic alteration leading to decreased copies in tumor relative to the control sample (log2 ratio between -0.5 and -1.0).
- *Amplification*: Focal, high copy number gain (log2 ratio >= 1.0).
- *Deletion*: Extensive copy number loss, likely corresponding to homozygous deletions (log2 ratio <= -1.0)
- *Partial*: A genomic alteration affecting part of a gene.
- Note that all genomic coordinates are based on human reference GRC37/hg19.

Treatement decisions: The treating physician is responsible to select the most appropriate course of treatment. Decision making about therapy should not be based solely on the information contained in this report.

List of clinically relevant genes:

ABL1, ABL2, AKT1, AKT2, AKT3, ALK, AR, AURKA, BCL2, BRAF, BRCA1, BRCA2, CALR, CD79B, CDK4, CDK6, CDKN2A, CDKN2B, CEBPA, CRKL, DNMT3A, EGFR, ERBB2, ERBB3, ERBB4, FGFR1, FGFR2, FGFR3, FGFR4, FLT3, GNA11, GNAQ, GNAS, HRAS, IDH1, IDH2, IKZF1, JAK2, KDM5C, KIT, KRAS, MAP2K1, MAP2K2, MCL1, MDM2, MET, NOTCH2, NRAS, PDGFRA, PIK3CA, PTCH1, PTEN, SMO, TSC1.

List of known cancer genes:

ABI1, ABL1, ABL2, ACKR3, ACSL3, ACSL6, ACVR1, AFF1, AFF3, AFF4, AKAP9, AKT1, AKT2, ALDH2, ALK, AMER1, APC, AR, ARHGAP26, ARHGEF12, ARID1A, ARID1B, ARID2, ARNT, ASPSCR1, ASXL1, ATF1, ATIC, ATM, ATP1A1, ATP2B3, ATR, ATRX, AXIN1, AXIN2, BAP1, BCL10, BCL11A, BCL11B, BCL2, BCL3, BCL5, BCL6, BCL7A, BCL9, BCOR, BCR, BIRC3, BLM, BMPR1A, BRAF, BRCA1, BRCA2, BRD3, BRD4, BRIP1, BTG1, BUB1B, C12orf9, C15orf65, C2orf44, CACNA1D, CALR, CAMTA1, CANT1, CARD11, CARS, CASC5, CASP8, CBFA2T3, CBFB, CBL, CBLB, CBLC, CCDC6, CCNB1IP1, CCND1, CCND2, CCND3, CCNE1, CD274, CD74, CD79A, CD79B, CDC73, CDH1, CDH11, CDK12, CDK4, CDK6, CDKN1B, CDKN2A, CDKN2C, CDKN2a(p14), CDX2, CEBPA, CEP89, CHCHD7, CHEK2, CHIC2, CHN1, CIC, CIITA, CLIP1, CLP1, CLTC, CLTCL1, CNBP, CNOT3, CNTRL, COL1A1, COL2A1, COX6C, CREB1, CREB3L1, CREB3L2, CREBBP, CRLF2, CRTC1, CRTC3, CSF3R, CTNNB1, CUX1, CYLD, DAXX, DCTN1, DDB2, DDIT3, DDX10, DDX5, DDX6, DEK, DICER1, DNM2, DNMT3A, DUX4L1, EBF1, ECT2L, EGFR, EIF3E, EIF4A2, ELF4, ELK4, ELL, ELN, EML4, EPHA3, EP300, EPS15, ERBB2, ERBB3, ERC1, ERCC2, ERCC3, ERCC4, ERCC5, ERG, ESR1, ETNK1, ETV1, ETV4, ETV5, ETV6, EWSR1, EXT1, EXT2, EZH2, EZR, FAM131B, FAM46C, FANCA, FANCC, FANCD2, FANCE, FANCF, FANCG, FAS, FBXO11, FBXW7, FCGR2B, FCRL4, FEV, FGFR1, FGFR1OP, FGFR2, FGFR3, FGFR4, FH, FHIT, FIP1L1, FLCN, FLI1, FLT3, FLT4, FNBP1, FOXA1, FOXL2, FOXO1, FOXO3, FOXO4, FOXP1, FSTL3, FUBP1, FUS, GAS7, GATA1, GATA2, GATA3, GMPS, GNA11, GNAQ, GNAS, GOLGA5, GOPC, GPC3, GPHN, GRIN2A, H3F3A, H3F3B, HERPUD1, HEY1, HIP1, HIST1H3B, HIST1H4I, HLA-A, HLF, HMGA1, HMGA2, HMGN2P46, HNF1A, HNRNPA2B1, HOOK3, HOXA11, HOXA13, HOXA9, HOXC11, HOXC13, HOXD11, HOXD13, HRAS, HSP90AA1, HSP90AB1, IDH1, IDH2, IGH, IGK, IGL, IKBKB, IKZF1, IL2, IL21R, IL6ST, IL7R, IRF4, ITK, JAK1, JAK2, JAK3, JAZF1, JUN, KAT6A, KAT6B, KCNJ5, KDM5A, KDM5C, KDM6A, KDR, KDSR, KIAA1549, KIAA1598, KIF5B, KIT, KLF4, KLF6, KLK2, KMT2A, KMT2C, KMT2D, KRAS, KTN1, LASP1, LCK, LCP1, LHFP, LIFR, LMNA, LMO1, LMO2, LPP, LRIG3, LSM14A, LYL1, MAF, MAFB, MALAT1, MALT1, MAML2, MAP2K1, MAP2K2, MAP2K4, MAP3K1, MAP3K13, MAX, MDM2, MDM4, MDS2, MECOM, MED12, MEN1, MET, MITF, MKL1, MLF1, MLH1, MLLT1, MLLT10, MLLT11, MLLT3, MLLT4, MLLT6, MN1, MNX1, MPL, MSH2, MSH6, MSI2, MSN, MTCP1, MUC1, MUTYH, MYB, MYC, MYCL, MYCN, MYD88, MYH11, MYH9, MYO5A, MYOD1, NAB2, NACA, NBN, NCKIPSD, NCOA1, NCOA2, NCOA4, NCOR1, NDRG1, NF1, NF2, NFATC2, NFE2L2, NFIB, NFKB2, NFKBIE, NIN, NKX2-1, NONO, NOTCH1, NOTCH2, NOTCH3, NPM1, NR4A3, NRAS, NRG1, NSD1, NT5C2, NTRK1, NTRK3, NUMA1, NUP214, NUP98, NUTM1, NUTM2A, NUTM2B, OLIG2, OMD, P2RY8, PAFAH1B2, PALB2, PAX3, PAX5, PAX7, PAX8, PBRM1, PBX1, PCM1, PCSK7, PDCD1LG2, PDE4DIP, PDGFB, PDGFRA, PDGFRB, PER1, PHF6, PHOX2B, PICALM, PIK3CA, PIK3R1, PIM1, PLAG1, PLCG1, PML, PMS1, PMS2, POLE, POT1, POU2AF1, POU5F1, PPARG, PPFIBP1, PPP2R1A, PPP6C, PRCC, PRDM1, PRDM16, PRF1, PRKAR1A, PRRX1, PSIP1, PTCH1, PTEN, PTPN11, PTPRB, PTPRC, PTPRK, PWWP2A, RABEP1, RAC1, RAD21, RAD51B, RAF1, RALGDS, RANBP17, RANBP2, RAP1GDS1, RARA, RB1, RBM15, RECQL4, REL, RET, RHOA, RHOH, RMI2, RNF213, RNF217-AS1, RNF43, ROS1, RPL10, RPL22, RPL5, RPN1, RSPO2, RSPO3, RUNDC2A, RUNX1, RUNX1T1, SBDS, SDC4, SDHAF2, SDHB, SDHC, SDHD, SEPT5, SEPT6, SEPT9, SET, SETBP1, SETD2, SF3B1, SFPQ, SH2B3, SH3GL1, SLC34A2, SLC45A3, SMAD4, SMARCA4, SMARCB1, SMARCD1, SMARCE1, SMO, SND1, SOCS1, SOX2, SPECC1, SPEN, SPOP, SRGAP3, SRSF2, SRSF3, SS18, SS18L1, SSX1, SSX2, SSX4, STAG2, STAT3, STAT5B, STAT6, STIL, STK11, STRN, SUFU, SUZ12, SYK, TAF15, TAL1, TAL2, TBL1XR1, TBX3, TCEA1, TCF12, TCF3, TCF7L2, TCL1A, TCL6, TERT, TET1, TET2, TFE3, TFEB, TFG, TFPT, TFRC, THRAP3, TLX1, TLX3, TMPRSS2, TNFAIP3, TNFRSF14, TNFRSF17, TOP1, TP53, TPM3, TPM4, TPR, TRA, TRAF7, TRB, TRD, TRIM24, TRIM27, TRIM33, TRIP11, TRRAP, TSC1, TSC2, TSHR, TTL, U2AF1, UBR5, USP6, VHL, VTI1A, WAS, WHSC1, WHSC1L1, WIF1, WRN, WT1, WWTR1, XPA, XPC, XPO1, YWHAE, ZBTB16, ZCCHC8, ZNF198, ZNF278, ZNF331, ZNF384, ZNF521, ZRSR2.

The report was generated at 13:34:29 EDT - Apr 11, 2016; based on version v1.6-14-g34708f2 of software IPM-reportGenerator, on version 0.1-86-g13c3a8c of the IPM knowledge base and cancer genes census, and on version 235e7f2-dirty of the results.

Copyright© 2013 - 2016 Cornell University. All Rights Reserved.
