# Supplementary material for: Development and validation of a whole-exome sequencing test for simultaneous detection of point mutations, indels and copy-number alterations for precision cancer care
Source: NPJ Genom Med. 2016 Jul 20;1:16019–. doi: 10.1038/npjgenmed.2016.19 (PMC5539963; doi:10.1038/npjgenmed.2016.19)
Supplement: Supplementary File 2 [file npjgenmed201619-s3.pdf]

CLINICAL INFORMATION

Patient ID: PMTEST
Requesting physician:

Tumor Type:
Primary site:
Tissue Tested:

Specimen IDs (case/control)
Sample type (case/control): /
Sample collected (case/control): ( ) / ()
Sample received (case/control): () / ()
Neoplastic content: (See Notes)
Ploidy: 1.76

SCNA Plot

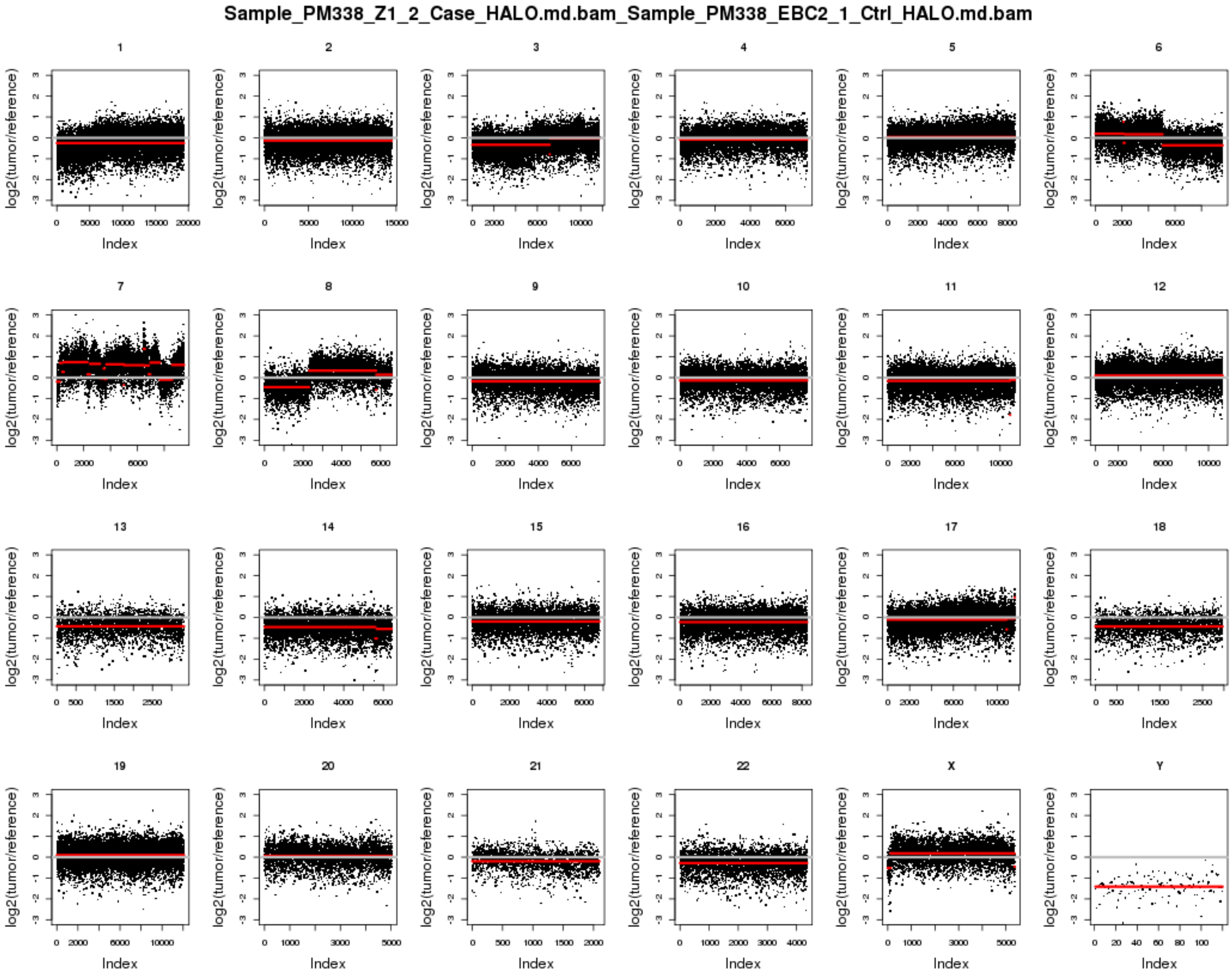

Alterations on chromosome Y (if any) should not be considered for female patients.

Tier 1 Somatic Copy Number Alterations

	Altered region (coordinates/cytoband)	CNA type	Genes	log2 (T/N)	Adj. log2 (CLONET)	CN (CLONET)	Absolute CN (GLOBAL)	CN Relative to ploidy
igv	7:115,594,665-117,120,173<br>q31.2	focal amplification	MET	1.38	1.37	NA	5.29	3.00

Depth: tumor read depth; VAF: variant allele frequency

Tier 2 somatic mutations and indels

	Gene name	Classification	Ref.	Allele 1	Allele 2	AA change	Tumor (normal) read depth	Tumor (normal) VAF	Tier
igv	WT1 chr11:32414251	missense	G	G	A	p.R434C	53 (103)	11.32% (0.00%)	Tier2

AA: amino-acid; VAF: variant allele frequency; Genomic coordinates are based on human reference GRC37/hg19 and are 1-based.
Alterations with VAF < 10%, coverage < 30x or < 5 mutated reads are below optimal detection conditions and should be considered as putative.

Tier 2 Somatic Copy Number Alterations

	Altered region (coordinates/cytoband)	CNA type	Genes	log2 (T/N)	Adj. log2 (CLONET)	CN (CLONET)	Absolute CN (GLOBAL)	CN Relative to ploidy
igv	14:102,551,699-107,283,201 q32.31-q32.33	broad copy number loss	AKT1	-0.55	-0.97	1	1.06	0.60
igv	7:117,149,140-124,477,264 q31.2-q31.33	focal copy number gain; partial	POT1	0.58	0.48	2	2.85	1.62
igv	7:128,040,906-135,661,810 q32.1-q33	broad copy number gain	SMO	0.72	0.64	NA	3.20	1.82
igv	7:2,255,575-4,007,006 p22.2-p22.3	focal copy number gain	CARD11	0.71	0.62	NA	3.15	1.79
igv	7:44,270,627-66,240,289 p11.1-q11.21	broad copy number gain	EGFR; IKZF1	0.66	0.57	3	3.04	1.73
igv	7:5,104,785-40,899,994 p14.1-p22.1	broad copy number gain	HOXA11; RAC1; HOXA13; HOXA9; JAZF1; PMS2; HNRNPA2B1; ETV1	0.74	0.66	NA	3.25	1.84
igv	7:75,695,705-99,227,226 q11.23-q22.1	broad copy number gain	CDK6; AKAP9	0.64	0.54	2	2.99	1.70

Depth: tumor read depth; VAF: variant allele frequency

Patient ID: PMTEST **Tumor type:** tumor type test **Primary site:** primary site test **Report date:** Apr. 11, 2016

Tier 3 somatic mutations and indels

	Gene name	Classification	Ref.	Allele 1	Allele 2	AA change	Tumor (normal) read depth	Tumor (normal) VAF	Tier
igv	WDR24<br>chr16:735965	missense	C	C	G	p.G493R	80 (126)	46.2% (0.0%)	Tier3
igv	NOS2<br>chr17:26101453	missense	G	G	T	p.H436N	34 (113)	32.4% (0.0%)	Tier3
igv	MIR138-1<br>chr3:44155717	frameshift insertion	-	+G	-	p.W6_fs	18 (28)	33.3% (0.0%)	Tier3
igv	MAP4K2<br>chr11:64566281	missense	T	T	A	p.K361M	54 (56)	51.9% (0.0%)	Tier3
igv	KIAA1683<br>chr19:18376380	missense	A	A	G	p.L657P	98 (112)	33.7% (0.0%)	Tier3
igv	DST<br>chr6:56464898	missense	A	A	G	p.V1769A	131 (109)	42.7% (0.0%)	Tier3
igv	VPS37A<br>chr8:17137801	missense	G	G	T	p.D299Y	23 (35)	60.9% (0.0%)	Tier3
igv	IGFN1<br>chr1:201180317	missense	A	A	G	p.E2099G	146 (108)	28.1% (0.0%)	Tier3
igv	MYO19<br>chr17:34852241	missense	T	T	C	p.K923E	27 (51)	40.7% (0.0%)	Tier3
igv	HECTD1<br>chr14:31598111	missense	G	G	A	p.P1489L	147 (210)	42.2% (0.0%)	Tier3
igv	HNRNPA1<br>chr12:54675583	missense	T	T	C	p.M46T	94 (87)	26.6% (0.0%)	Tier3
igv	BCKDHA<br>chr19:41916704	missense	C	C	A	p.P91T	74 (85)	40.5% (0.0%)	Tier3
igv	MYO9A<br>chr15:72144533	frameshift deletion	AAGCC	-	AAGCC	p.R2137_fs	21 (33)	52.4% (0.0%)	Tier3
igv	TTF2<br>chr1:117618165	missense	A	A	C	p.H320P	203 (124)	25.6% (0.0%)	Tier3
igv	TIMD4<br>chr5:156376689	missense	C	C	T	p.A245T	235 (185)	31.1% (0.0%)	Tier3
igv	MESDC2<br>chr15:81282126	missense	C	C	T	p.A3T	40 (49)	67.5% (0.0%)	Tier3
igv	FAM136A<br>chr2:70524488	missense	T	T	C	p.D117G	43 (124)	37.2% (0.0%)	Tier3
igv	ANKRD10<br>chr13:111567160	missense	A	A	T	p.L41Q	45 (61)	48.9% (0.0%)	Tier3
igv	MORF4L1<br>chr15:79170574	missense	T	T	A	p.F20L	38 (67)	39.5% (0.0%)	Tier3
igv	DNAH8<br>chr6:38709496	missense	G	G	C	p.E376Q	89 (60)	29.2% (0.0%)	Tier3

Patient ID: PMTEST **Tumor type:** tumor type test **Primary site:** primary site test **Report date:** Apr. 11, 2016

	Gene name	Classification	Ref.	Allele 1	Allele 2	AA change	Tumor (normal) read depth	Tumor (normal) VAF	Tier
igv	SLC35F6 chr2:26997987	missense	T	T	G	p.S76A	52 (62)	36.5% (0.0%)	Tier3
igv	UBE2J1 chr6:90045086	missense	A	A	G	p.S165P	54 (43)	44.4% (0.0%)	Tier3
igv	FLG2 chr1:152327773	missense	G	G	T	p.S830Y	176 (281)	42.6% (0.0%)	Tier3
igv	MAGEC3 chrX:140985485	missense	T	T	A	p.F600Y	132 (109)	65.2% (0.0%)	Tier3
igv	C11orf87 chr11:109294576	missense	T	T	C	p.C73R	69 (146)	59.4% (0.0%)	Tier3
igv	PKHD1L1 chr8:110471831	nonsense	C	C	T	p.Q2338X	64 (67)	39.1% (0.0%)	Tier3
igv	TKT chr3:53267183	missense	C	C	T	p.R254Q	32 (49)	31.2% (0.0%)	Tier3
igv	DNAH9 chr17:11535915	missense	T	T	G	p.N510K	62 (93)	40.3% (0.0%)	Tier3
igv	VCPIP1 chr8:67577498	missense	T	T	C	p.R566G	17 (27)	58.8% (0.0%)	Tier3
igv	TMEM139 chr7:142983780	missense	C	C	T	p.A170V	142 (117)	28.9% (0.0%)	Tier3
igv	TAS2R7 chr12:10954392	missense	T	T	A	p.M260L	281 (208)	33.5% (0.0%)	Tier3
igv	FAM120B chr6:170627739	missense	G	G	C	p.A444P	36 (52)	47.2% (0.0%)	Tier3

AA: amino-acid; VAF: variant allele frequency; Genomic coordinates are based on human reference GRC37/hg19 and are 1-based.
Alterations with VAF < 10%, coverage < 30x or < 5 mutated reads are below optimal detection conditions and should be considered as putative.

Tier 3 Somatic Copy Number Alterations

	Altered region (coordinates/cytoband)	CNA type	Genes	log2 (T/N)	Adj. log2 (CLONET)	CN (CLONET)	Absolute CN (GLOBAL)	CN Relative to ploidy	# genes
igv	11:126,135,915-126,136,735 q24.2	focal deletion; partial	SRPR	-1.79	-3.47	NA	0.19	0.11	1
igv	14:102,452,560-102,551,234 q32.31	focal deletion	HSP90AA1; DYNC1H1	-1.01	-1.67	0	0.65	0.37	2
igv	14:102,551,699-107,283,201 q32.31-q32.33	broad copy number loss	too many to show	-0.55	-0.97	1	1.06	0.60	64
igv	17:78,263,545-78,367,219 q25.3	focal copy number loss	LOC100294362; RNF213	-0.57	-1.00	1	1.03	0.59	2
igv	3:121,634,499-121,658,267 q13.33	focal copy number loss; partial	SLC15A2	-0.79	-1.32	0	0.83	0.47	1
igv	7:115,594,665-117,120,173 q31.2	focal amplification	too many to show	1.38	1.37	NA	5.29	3.00	13
igv	7:117,149,140-124,477,264 q31.2-q31.33	focal copy number gain	too many to show	0.58	0.48	2	2.85	1.62	29
igv	7:128,040,906-135,661,810 q32.1-q33	broad copy number gain	too many to show	0.72	0.64	NA	3.20	1.82	71
igv	7:148,823,262-158,937,225 q36.1-q36.3	broad copy number gain	too many to show	0.61	0.51	3	2.93	1.66	98
igv	7:2,255,575-4,007,006 p22.2-p22.3	focal copy number gain	too many to show	0.71	0.62	NA	3.15	1.79	14
igv	7:44,270,627-66,240,289 p11.1-q11.21	broad copy number gain	too many to show	0.66	0.57	3	3.04	1.73	106

	Altered region (coordinates/cytoband)	CNA type	Genes	log2 (T/N)	Adj. log2 (CLONET)	CN (CLONET)	Absolute CN (GLOBAL)	CN Relative to ploidy	# genes
igv	7:5,104,785-40,899,994 p14.1-p22.1	broad copy number gain	too many to show	0.74	0.66	NA	3.25	1.84	217
igv	7:75,695,705-99,227,226 q11.23-q22.1	broad copy number gain	too many to show	0.64	0.54	2	2.99	1.70	136
igv	7:99,261,652-115,590,963 q22.1-q31.2	broad copy number gain	too many to show	0.60	0.50	3	2.89	1.64	159
igv	8:142,500,294-143,310,890 q24.3	focal copy number loss	too many to show	-0.58	-1.01	0	1.02	0.58	4
igv	X:200,916-2,688,609 p22.33	focal copy number loss	LINC00102; XG	-0.52	-0.92	NA	1.10	0.62	2
igv	Y:2,655,335-23,763,771 p11.1-q11.223	broad deletion	too many to show	-1.41	-2.47	NA	0.38	0.22	74

Depth: tumor read depth; VAF: variant allele frequency

HSP90AA1

14:102546073-102607086

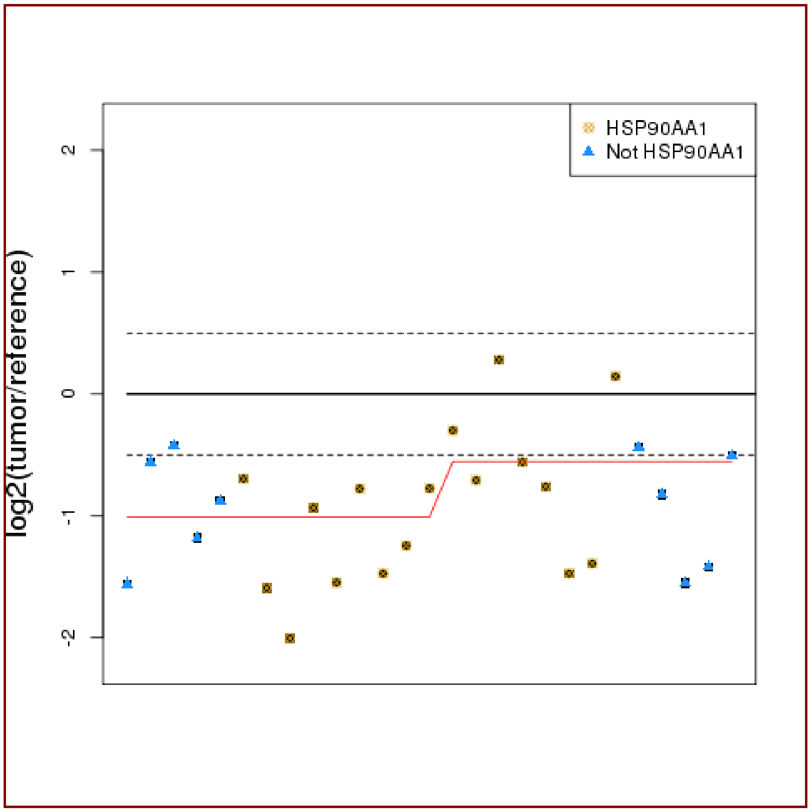

AKT1

14:105234685-105263080

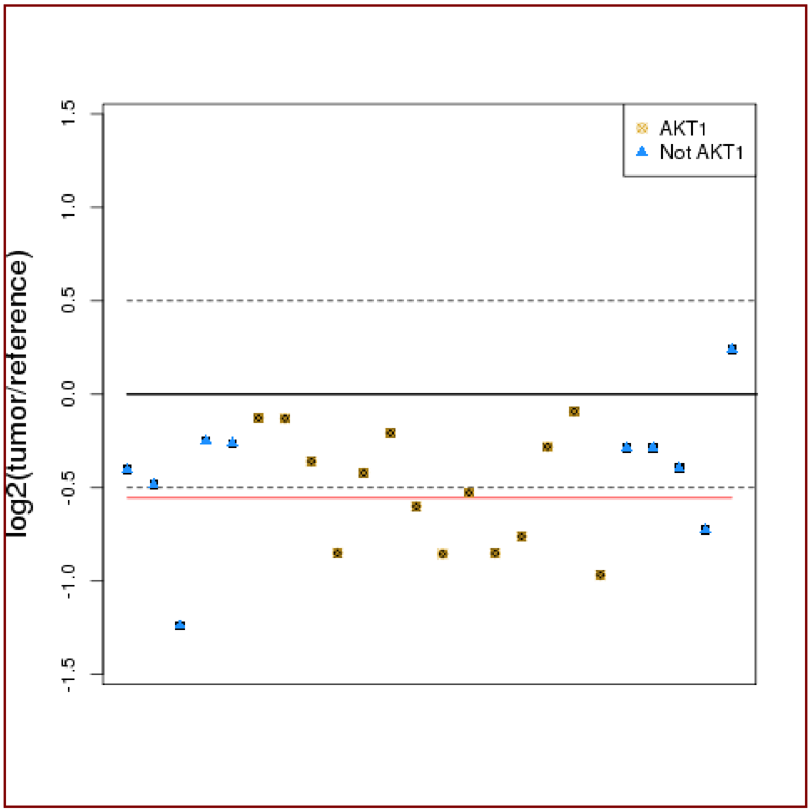

MET

7:116311457-116439440

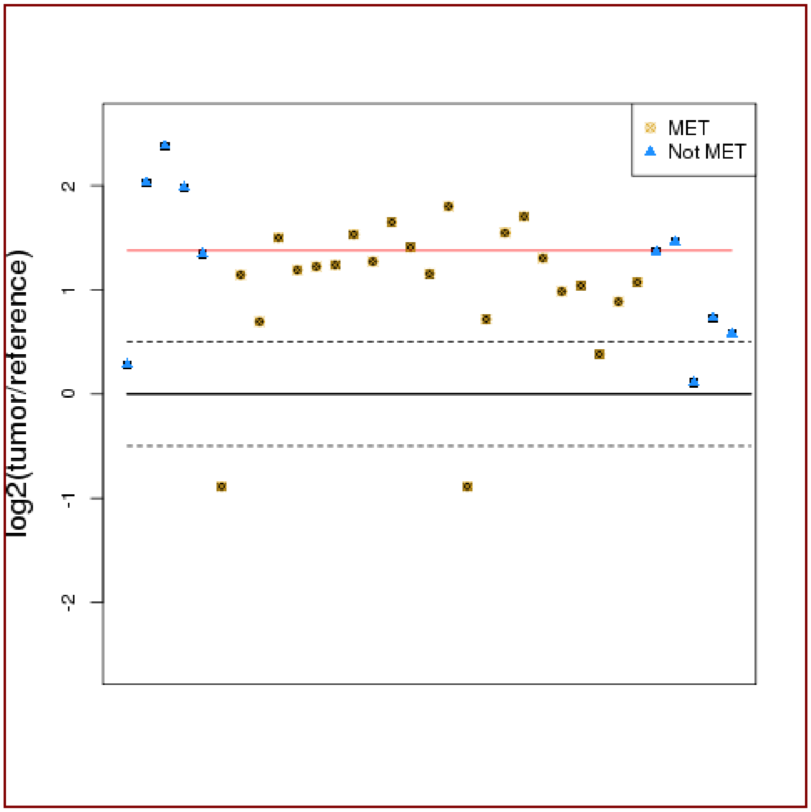

POT1

7:124461438-124571037

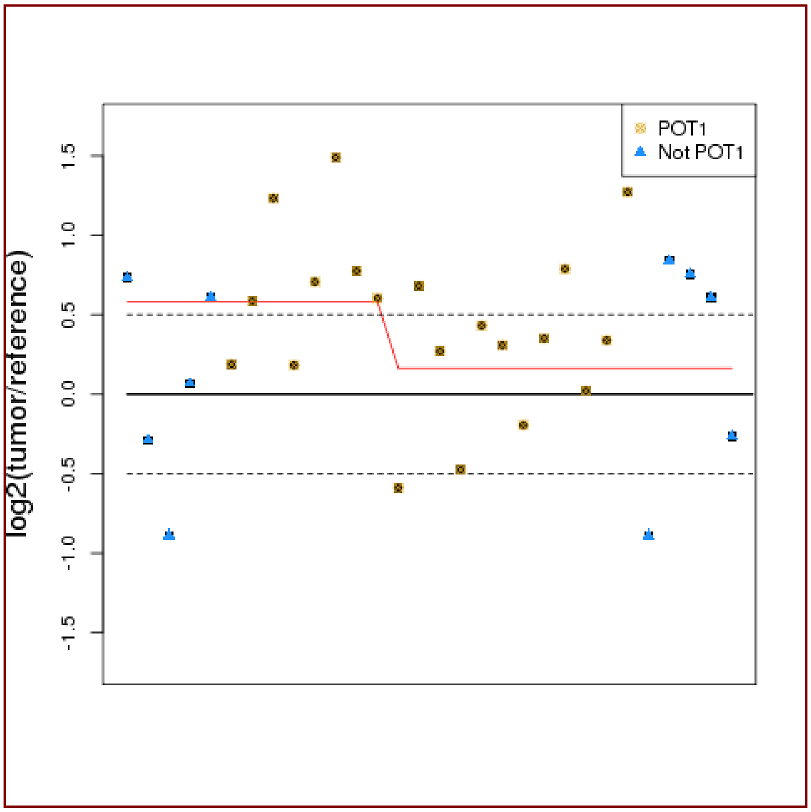

SMO
7:128827711-128854385

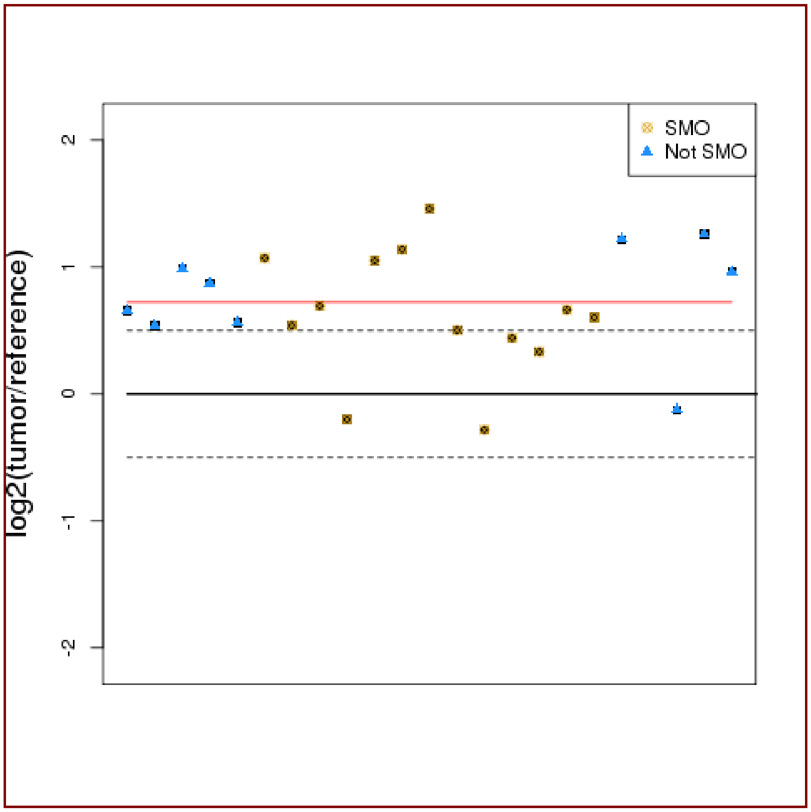

ETV1
7:13929854-14032050

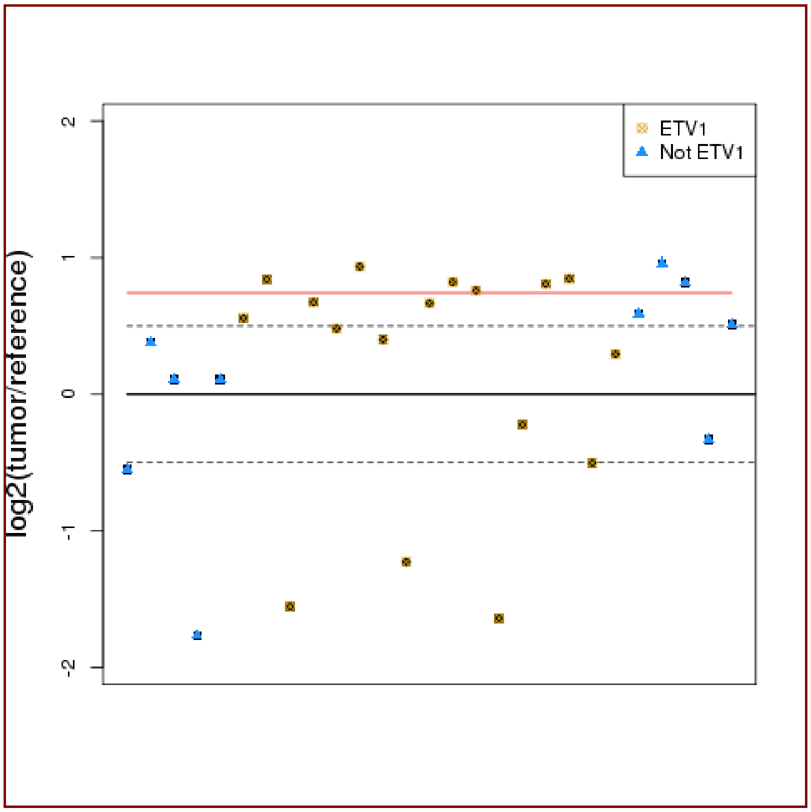

HNRNPA2B1

7:26228554-26241413

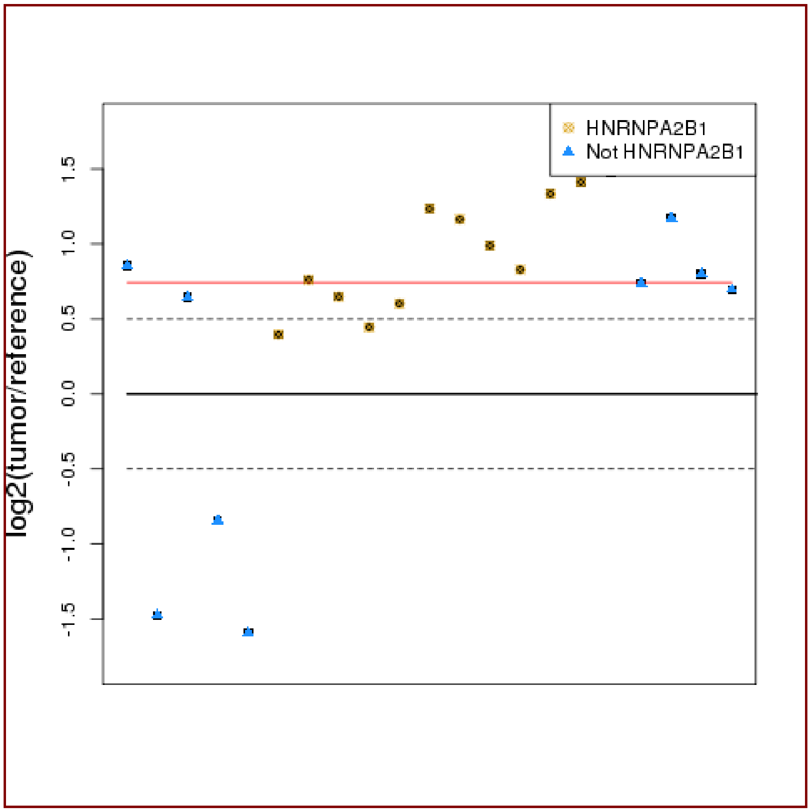

HOXA9

7:27201055-27206149

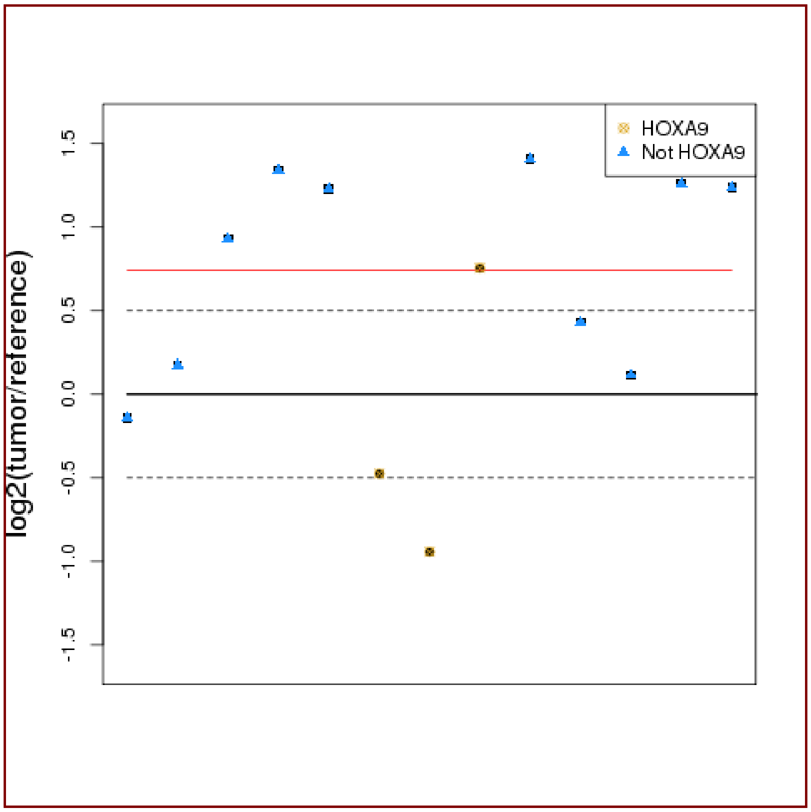

HOXA11
7:27219774-27225835

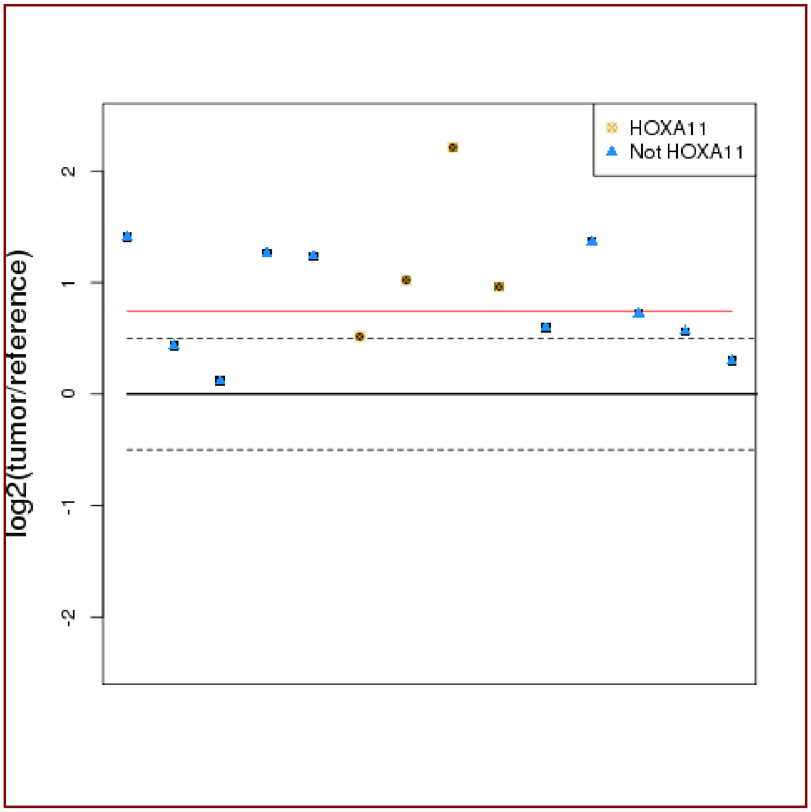

HOXA13
7:27235497-27240725

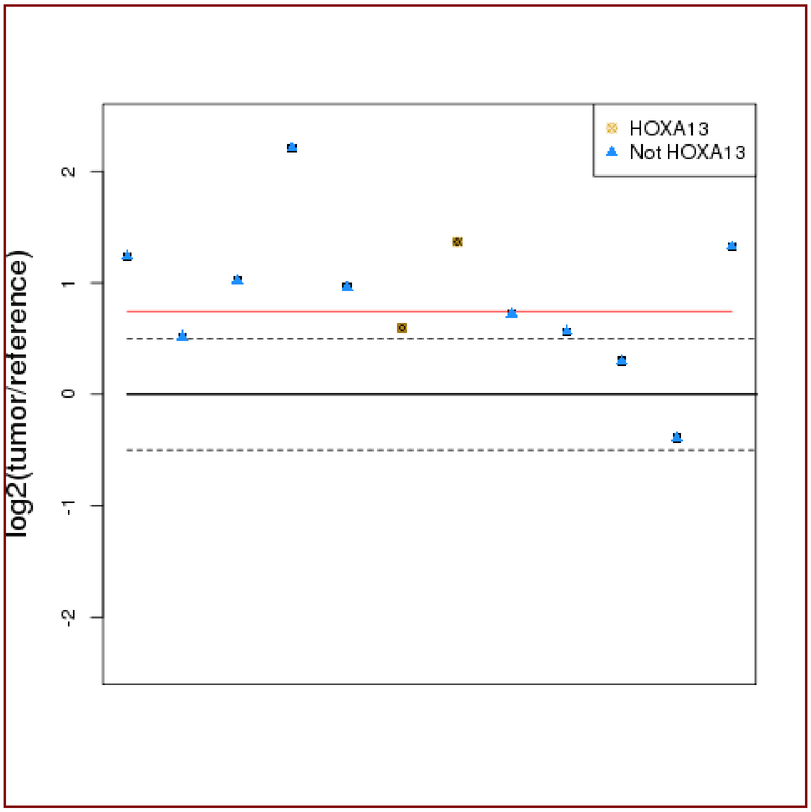

JAZF1

7:27869191-28221437

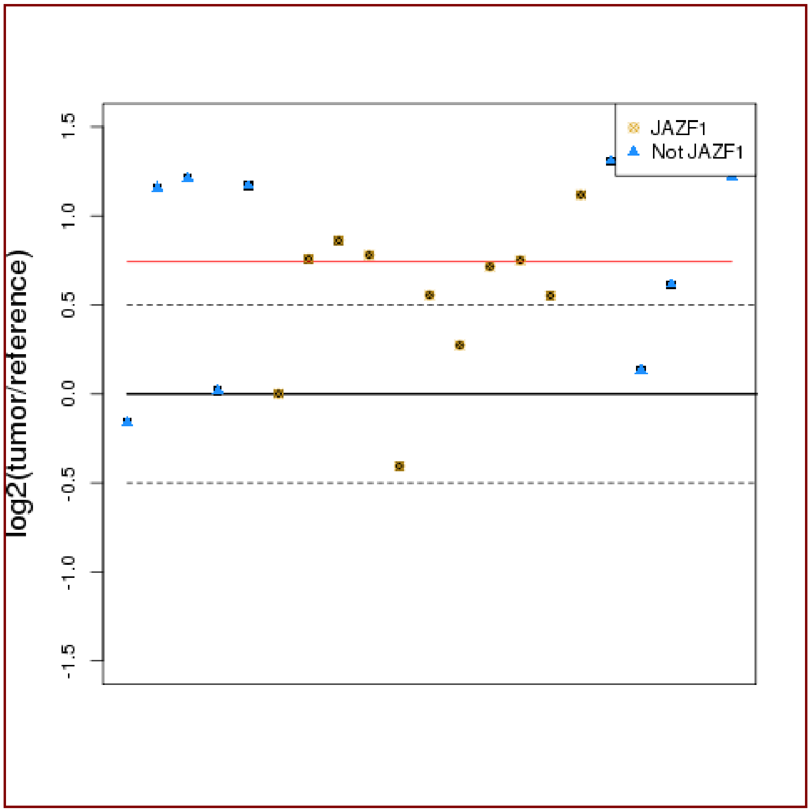

CARD11

7:2944708-3084509

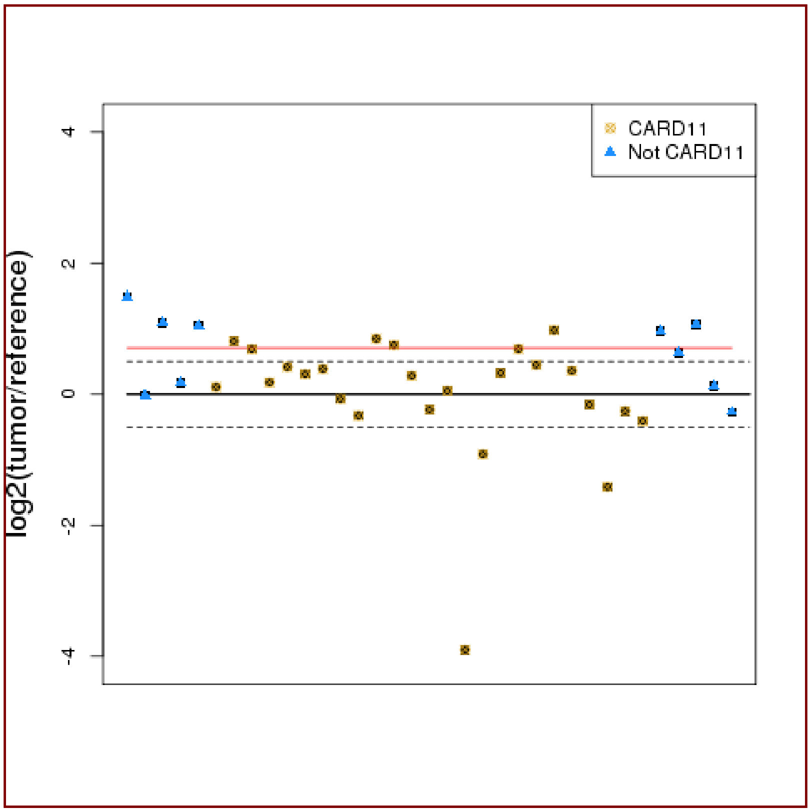

FKBP9

7:32996003-33047543

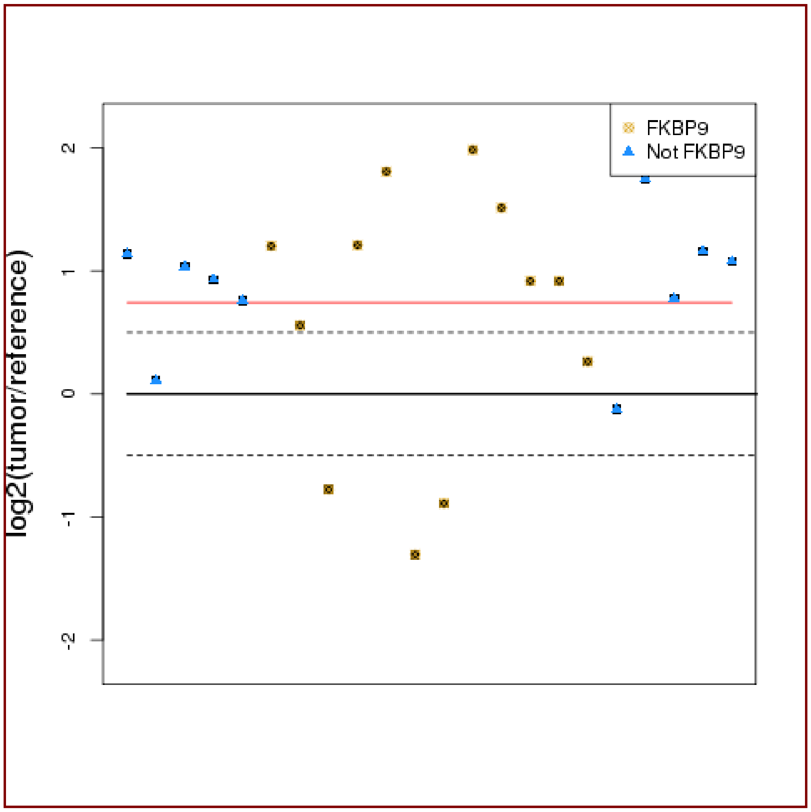

IKZF1

7:50343376-50473798

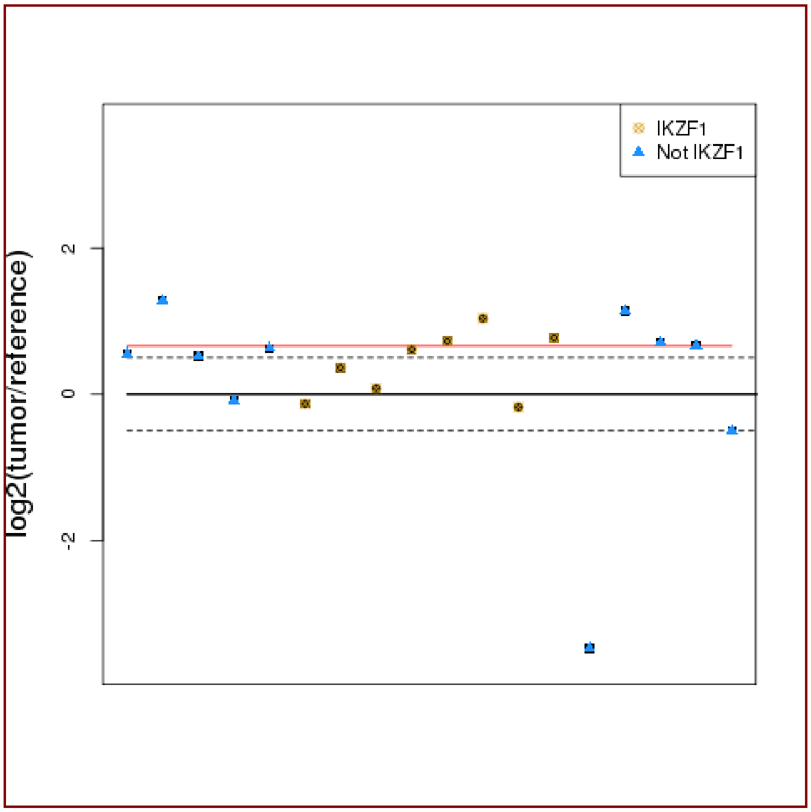

EGFR
7:55085723-55276031

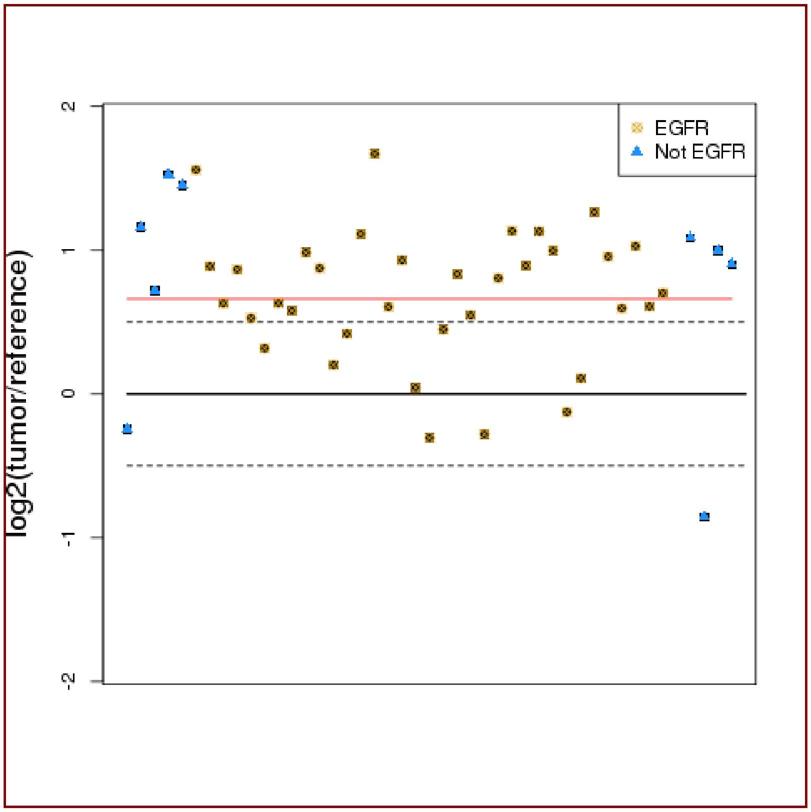

PMS2
7:6011868-6049737

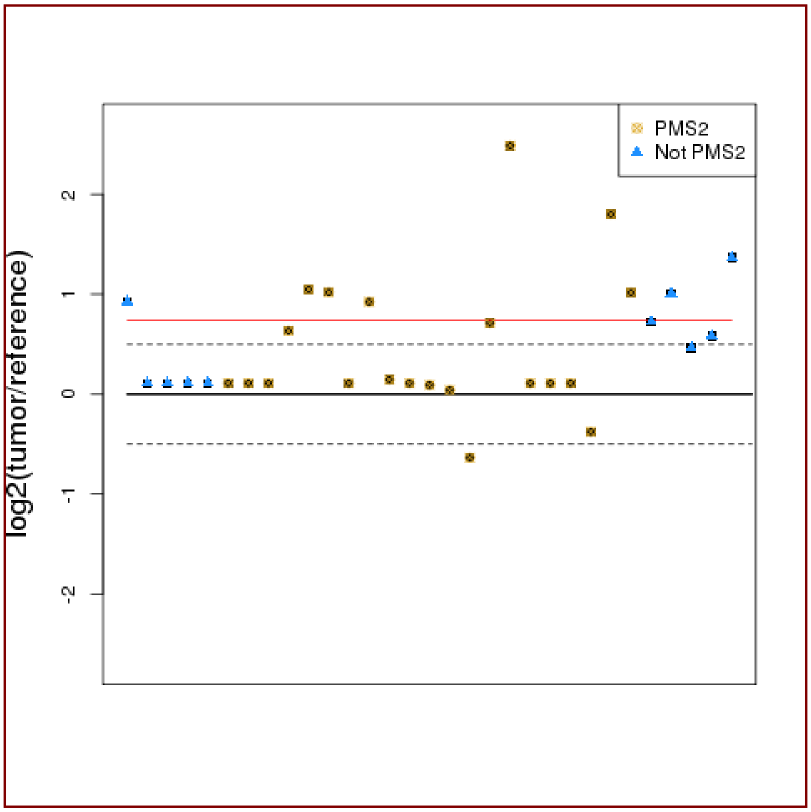

RAC1

7:6413124-6444598

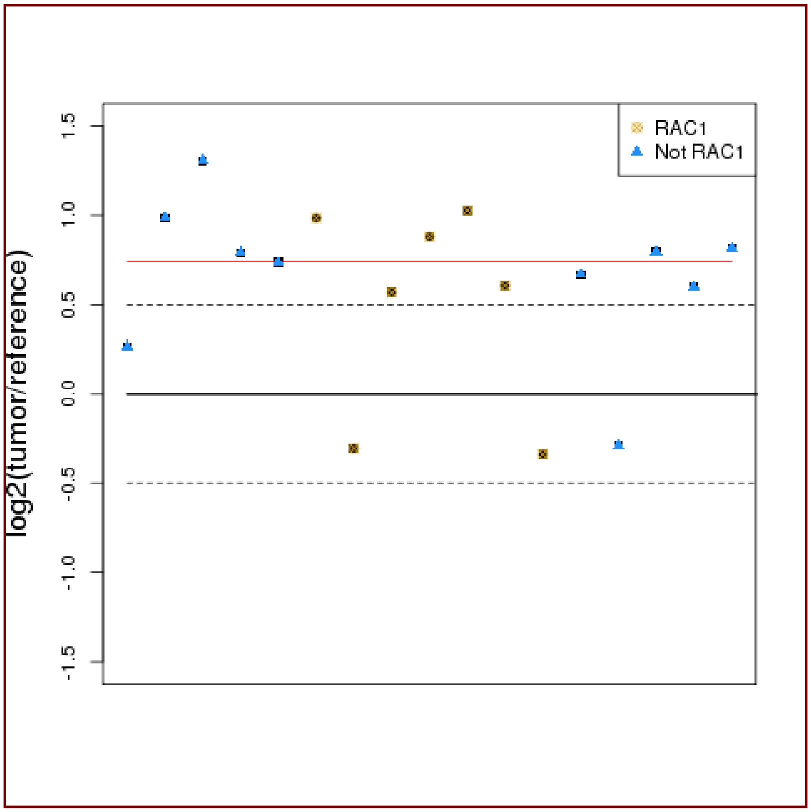

AKAP9

7:91569187-91740987

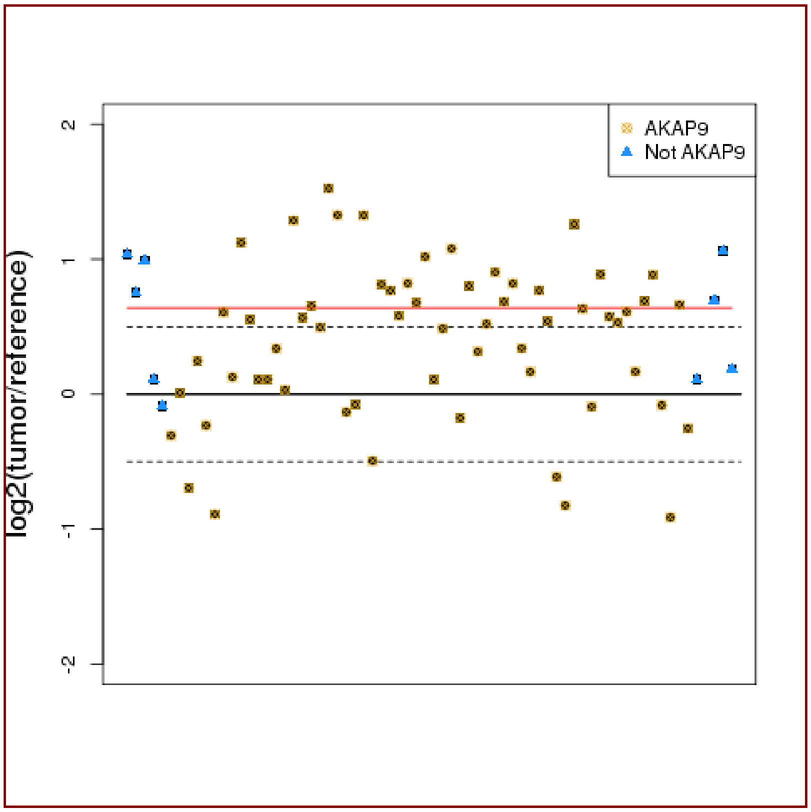

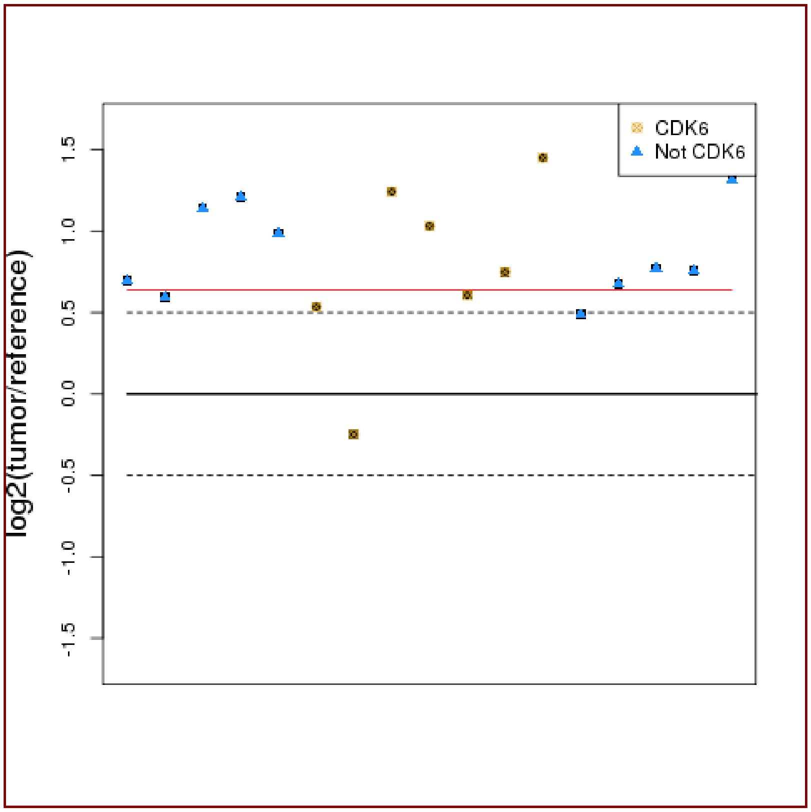

Manually filtered somatic mutations and indels

	Gene name	Classification	Ref.	Allele 1	Allele 2	AA change	Tumor (normal) read depth	Tumor (normal) VAF	Tier
igv	FGFR4 chr5:176518692	missense	C	C	T	p.H204Y	40 (98)	5.00% (0.00%)	curated
igv	NOTCH1 chr9:139399237	missense	C	C	T	p.E1636K	50 (40)	8.00% (0.00%)	curated
igv	NF2 chr22:30069387	missense	C	C	T	p.R418C	44 (78)	9.09% (0.00%)	curated
igv	JAK2 chr9:5089702	missense	G	G	A	p.R867Q	62 (52)	6.45% (0.00%)	curated
igv	NOTCH1 chr9:139401281	missense	C	C	T	p.R1263H	43 (74)	9.30% (0.00%)	curated
igv	APC chr5:112177902	missense	G	G	T	p.R2204L	33 (50)	6.06% (0.00%)	curated
igv	JAK2 chr9:5073756	missense	A	A	G	p.N612S	31 (45)	6.45% (0.00%)	curated
igv	MAP2K4 chr17:12032525	missense	G	G	A	p.V332M	45 (60)	8.89% (0.00%)	curated
igv	PTPN11 chr12:112926270	missense	C	C	T	p.T468M	37 (64)	5.41% (0.00%)	curated

Patient ID: PMTEST **Tumor type:** tumor type test **Primary site:** primary site test **Report date:** Apr. 11, 2016

	Gene name	Classification	Ref.	Allele 1	Allele 2	AA change	Tumor (normal) read depth	Tumor (normal) VAF	Tier
igv	APC chr5:112173704	nonsense	C	C	T	p.R805X	120 (147)	5.00% (0.00%)	curated
igv	KDR chr4:55958791	missense	G	G	A	p.S1021L	69 (74)	5.80% (0.00%)	curated
igv	RICTOR chr5:38955713	missense	G	G	A	p.R865C	58 (77)	6.90% (0.00%)	curated
igv	TGFBR2 chr3:30713753	missense	C	C	T	p.H385Y	34 (46)	5.88% (0.00%)	curated
igv	FLT4 chr5:180048796	missense	C	C	T	p.R589H	66 (43)	6.06% (0.00%)	curated
igv	TGFBR2 chr3:30713751	missense	C	C	T	p.A384V	34 (46)	5.88% (0.00%)	curated
igv	ZNF668 chr16:31072900	missense	G	G	A	p.A473V	37 (84)	5.41% (0.00%)	curated
igv	TGFBR2 chr3:30713763	missense	G	G	T	p.S388I	34 (47)	5.88% (0.00%)	curated
igv	KEAP1 chr19:10599969	missense	C	C	T	p.R536H	57 (125)	5.26% (0.00%)	curated
igv	MRE11A chr11:94203768	missense	T	T	C	p.M296V	40 (66)	5.00% (0.00%)	curated
igv	GUCY1A2 chr11:106579268	missense	C	C	T	p.R685Q	33 (37)	6.06% (0.00%)	curated
igv	KEAP1 chr19:10602854	missense	C	C	T	p.E242K	31 (54)	6.45% (0.00%)	curated
igv	KRTAP4-6 chr17:39296223	frameshift deletion	AC	-	AC	p.P172_fs	55 (52)	67.3% (0.0%)	curated
igv	KRTAP4-6 chr17:39296218	frameshift deletion	A	-	A	p.V174_fs	48 (30)	77.1% (0.0%)	curated
igv	KRTAP4-6 chr17:39296238	missense	A	A	T	p.C168S	57 (52)	68.4% (0.0%)	curated
igv	KCNN3 chr1:154842266	frameshift deletion	C	-	C	p.L58_fs	27 (33)	40.7% (0.0%)	curated
igv	KCNN3 chr1:154842241	inframe deletion	GAA	-	GAA	p.Q66_nofs	12 (42)	33.3% (0.0%)	curated
igv	KCNN3 chr1:154842264	frameshift deletion	A	-	A	p.L59_fs	22 (20)	50.0% (0.0%)	curated

AA: amino-acid; VAF: variant allele frequency; Genomic coordinates are based on human reference GRC37/hg19 and are 1-based.
Alterations with VAF < 10%, coverage < 30x or < 5 mutated reads are below optimal detection conditions and should be considered as putative.

Filtered somatic mutations and indels

This table includes all filtered variants with: **a.** tumor VAF >= 5%; **b.** (tumor VAF) – (normal VAF) >= 5%. **c.** tumor and normal depth >= 5x; No silent mutations are reported.
Up to 3000 alterations are listed. For the full list of alterations, please contact the IPM computational team at pmcomp@med.cornell.edu.

	Gene name	Classification	Ref.	Allele 1	Allele 2	AA change	Tumor (normal) read depth	Tumor (normal) VAF	Tier
igv	PTPRD chr9:8485834	missense	G	G	A	p.R995C	158 (105)	77.22% (32.38%)	filtered
igv	NOTCH1 chr9:139401309	missense	T	T	C	p.T1254A	6 (34)	33.33% (0.00%)	filtered
igv	NOTCH1 chr9:139401310	nonsense	G	G	T	p.C1253X	6 (34)	33.33% (0.00%)	filtered
igv	MYC chr8:128750761	missense	G	G	T	p.D100Y	7 (16)	28.57% (0.00%)	filtered
igv	TET2 chr4:106155751	missense	G	G	A	p.V218M	33 (55)	84.85% (58.18%)	filtered
igv	KIT chr4:55593464	missense	A	A	C	p.M541L	11 (26)	90.91% (65.38%)	filtered
igv	CTNNB1 chr3:41266998	missense	T	T	A	p.H223Q	12 (33)	25.00% (3.03%)	filtered
igv	PIK3R1 chr5:67588148	missense	G	G	A	p.M326I	131 (152)	63.36% (44.08%)	filtered
igv	NTRK1 chr1:156843535	missense	G	G	A	p.V321M	11 (14)	18.18% (0.00%)	filtered
igv	CTNNB1 chr3:41267183	frameshift deletion	T	T	T	c.767_767delins-T	6 (28)	16.67% (0.00%)	filtered
igv	RICTOR chr5:38947454	missense	A	A	G	p.V1433A	6 (14)	16.67% (0.00%)	filtered
igv	NOTCH1 chr9:139391518	missense	G	G	T	p.Q2225K	14 (30)	14.29% (0.00%)	filtered
igv	PTCH1 chr9:98248121	missense	G	G	A	p.R144C	14 (17)	14.29% (0.00%)	filtered
igv	FGFR3 chr4:1803606	missense	A	A	T	p.N262Y	7 (13)	14.29% (0.00%)	filtered
igv	KDR chr4:55961025	nonsense	G	G	T	p.S972X	7 (13)	14.29% (0.00%)	filtered
igv	PIK3CA chr3:178921522	missense	G	G	A	p.R335K	7 (18)	14.29% (0.00%)	filtered
igv	NOTCH2 chr1:120458338	missense	A	A	T	p.L2336Q	16 (13)	12.50% (0.00%)	filtered
igv	FLT3 chr13:28608440	missense	T	T	C	p.K568E	17 (18)	11.76% (0.00%)	filtered

Patient ID: PMTEST **Tumor type:** tumor type test **Primary site:** primary site test **Report date:** Apr. 11, 2016

	Gene name	Classification	Ref.	Allele 1	Allele 2	AA change	Tumor (normal) read depth	Tumor (normal) VAF	Tier
igv	ABL2 chr1:179077955	missense	A	A	T	p.V816E	9 (16)	11.11% (0.00%)	filtered
igv	BCL2 chr18:60985542	missense	G	G	C	p.H120D	19 (18)	10.53% (0.00%)	filtered
igv	RB1 chr13:48881459	missense	T	T	C	p.C61R	19 (35)	10.53% (0.00%)	filtered
igv	NOTCH4 chr6:32191659	frameshift deletion	AGC	AGC	AGC	c.45_47GCT-	64 (98)	46.88% (36.73%)	filtered
igv	APC chr5:112177907	frameshift deletion	A	A	A	c.6616_6616delins-A	20 (41)	10.00% (0.00%)	filtered
igv	CEBPA chr19:33792929	missense	T	T	C	p.Y166C	20 (10)	10.00% (0.00%)	filtered
igv	ERBB2 chr17:37881348	missense	C	C	A	p.A847D	20 (59)	10.00% (0.00%)	filtered
igv	TSC1 chr9:135781313	missense	G	G	T	p.A551D	20 (50)	10.00% (0.00%)	filtered
igv	SMARCA4 chr19:11169536	missense	G	G	A	p.A1568T	10 (28)	10.00% (0.00%)	filtered
igv	KIT chr4:55594195	missense	A	A	G	p.E633G	21 (34)	9.52% (0.00%)	filtered
igv	JAK1 chr1:65332800	missense	A	A	G	p.F247L	11 (53)	9.09% (0.00%)	filtered
igv	TET2 chr4:106158366	missense	A	A	T	p.E1089D	11 (30)	9.09% (0.00%)	filtered
igv	SMARCA4 chr19:11143999	missense	G	G	A	p.G1194R	23 (16)	8.70% (0.00%)	filtered
igv	NOTCH1 chr9:139391020	missense	G	G	C	p.Q2391E	12 (38)	8.33% (0.00%)	filtered
igv	CEBPA chr19:33793124	missense	G	G	T	p.A101D	25 (15)	8.00% (0.00%)	filtered
igv	HNF1A chr12:121416650	missense	A	A	C	p.I27L	363 (177)	57.02% (49.15%)	filtered
igv	NF1 chr17:29588775	missense	C	C	A	p.L1542I	26 (43)	7.69% (0.00%)	filtered
igv	NTRK3 chr15:88476388	missense	G	G	A	p.R582W	13 (15)	7.69% (0.00%)	filtered
igv	APC chr5:112162848	missense	A	A	T	p.E484D	14 (71)	7.14% (0.00%)	filtered
igv	CDKN2A chr9:21971137	missense	T	T	G	p.D74A	14 (17)	7.14% (0.00%)	filtered
igv	FBXW7 chr4:153244185	nonsense	G	G	A	p.R658X	14 (41)	7.14% (0.00%)	filtered

Patient ID: PMTEST **Tumor type:** tumor type test **Primary site:** primary site test **Report date:** Apr. 11, 2016

	Gene name	Classification	Ref.	Allele 1	Allele 2	AA change	Tumor (normal) read depth	Tumor (normal) VAF	Tier
igv	FLT1 chr13:29004242	missense	G	G	A	p.R351W	14 (43)	7.14% (0.00%)	filtered
igv	IGF1R chr15:99456462	missense	T	T	A	p.H593Q	14 (54)	7.14% (0.00%)	filtered
igv	JAK2 chr9:5050712	missense	G	G	T	p.W165C	14 (39)	7.14% (0.00%)	filtered
igv	NF1 chr17:29587491	nonsense	G	G	A	p.W1512X	14 (46)	7.14% (0.00%)	filtered
igv	NTRK1 chr1:156843515	missense	G	G	A	p.R314H	14 (31)	7.14% (0.00%)	filtered
igv	TOP1 chr20:39744035	missense	A	A	T	p.M555L	14 (27)	7.14% (0.00%)	filtered
igv	ATM chr11:108186765	missense	G	G	T	p.M2041I	15 (45)	6.67% (0.00%)	filtered
igv	EPHA5 chr4:66201773	nonsense	A	A	T	p.L911X	15 (31)	6.67% (0.00%)	filtered
igv	MAP2K4 chr17:12028632	missense	G	G	A	p.A290T	15 (59)	6.67% (0.00%)	filtered
igv	MSH6 chr2:48026861	missense	C	C	T	p.S580L	15 (50)	6.67% (0.00%)	filtered
igv	RUNX1T1 chr8:92982935	missense	G	G	A	p.A556V	15 (29)	6.67% (0.00%)	filtered
igv	TET2 chr4:106196895	missense	A	A	T	p.N1743I	15 (30)	6.67% (0.00%)	filtered
igv	AURKA chr20:54945247	nonsense	G	G	T	p.C393X	16 (41)	6.25% (0.00%)	filtered
igv	MLH1 chr3:37081725	missense	C	C	T	p.P536L	16 (47)	6.25% (0.00%)	filtered
igv	CDH1 chr16:68835780	missense	G	G	A	p.R124H	17 (42)	5.88% (0.00%)	filtered
igv	FBXW7 chr4:153259064	missense	G	G	A	p.L251F	17 (22)	5.88% (0.00%)	filtered
igv	RB1 chr13:48916818	frameshift deletion	T	T	T	c.348_348delins-T	17 (40)	5.88% (0.00%)	filtered
igv	NOTCH4 chr6:32191658		T	T	TAGC	c.48_48delinsGCTA	64 (98)	34.38% (28.57%)	filtered
igv	CALR chr19:13054614	nonsense	G	G	T	p.E381X	18 (23)	5.56% (0.00%)	filtered
igv	PTPRD chr9:8521514	missense	G	G	T	p.P242T	18 (42)	5.56% (0.00%)	filtered
igv	PTPRD chr9:8521516	missense	G	G	T	p.P241Q	18 (42)	5.56% (0.00%)	filtered

Patient ID: PMTEST **Tumor type:** tumor type test **Primary site:** primary site test **Report date:** Apr. 11, 2016

	Gene name	Classification	Ref.	Allele 1	Allele 2	AA change	Tumor (normal) read depth	Tumor (normal) VAF	Tier
igv	STK11<br>chr19:1220625	missense	G	G	A	p.G215S	18 (34)	5.56% (0.00%)	filtered
igv	APC<br>chr5:112175330	missense	G	G	T	p.A1347S	19 (34)	5.26% (0.00%)	filtered
igv	TNFAIP3<br>chr6:138192482	missense	T	T	C	p.F40L	19 (53)	5.26% (0.00%)	filtered
igv	MYC<br>chr8:128752952	missense	G	G	T	p.R371S	20 (30)	5.00% (0.00%)	filtered
igv	PTPRD<br>chr9:8389287	missense	C	C	A	p.W1444L	20 (41)	5.00% (0.00%)	filtered
igv	TSHR<br>chr14:81609530	missense	G	G	T	p.E376D	20 (37)	5.00% (0.00%)	filtered

AA: amino-acid; VAF: variant allele frequency; Genomic coordinates are based on human reference GRC37/hg19 and are 1-based.
Alterations with VAF < 10%, coverage < 30x or < 5 mutated reads are below optimal detection conditions and should be considered as putative.

Files used:

- PM338_Z1_2_Case_HALO_Phase1_uid1092__PM338_EBC2_1_Ctrl_HALO_Phase1_uid854_Phase2_uid1093/CLONET_clonalityTable_1.0.txt
- PM338_Z1_2_Case_HALO_Phase1_uid1092__PM338_EBC2_1_Ctrl_HALO_Phase1_uid854_Phase2_uid1093/CLONET_global.Adm.Table_1.0.txt
- PM338_Z1_2_Case_HALO_Phase1_uid1092__PM338_EBC2_1_Ctrl_HALO_Phase1_uid854_Phase2_uid1093/Sample_PM338_Z1_2_Case_HALO.Sample_PM338_EBC2_1_Ctrl_HALO.CLIA.MAF
- PM338_Z1_2_Case_HALO_Phase1_uid1092__PM338_EBC2_1_Ctrl_HALO_Phase1_uid854_Phase2_uid1093/Sample_PM338_Z1_2_Case_HALO.Sample_PM338_EBC2_1_Ctrl_HALO.COSMIC.MAF
- PM338_Z1_2_Case_HALO_Phase1_uid1092__PM338_EBC2_1_Ctrl_HALO_Phase1_uid854_Phase2_uid1093/Sample_PM338_Z1_2_Case_HALO.Sample_PM338_EBC2_1_Ctrl_HALO.COSMIC.filtered.MAF
- PM338_Z1_2_Case_HALO_Phase1_uid1092__PM338_EBC2_1_Ctrl_HALO_Phase1_uid854_Phase2_uid1093/Sample_PM338_Z1_2_Case_HALO.Sample_PM338_EBC2_1_Ctrl_HALO.CancerGenes.MAF
- PM338_Z1_2_Case_HALO_Phase1_uid1092__PM338_EBC2_1_Ctrl_HALO_Phase1_uid854_Phase2_uid1093/Sample_PM338_Z1_2_Case_HALO.Sample_PM338_EBC2_1_Ctrl_HALO.ClinicallyRelevant.MAF
- PM338_Z1_2_Case_HALO_Phase1_uid1092__PM338_EBC2_1_Ctrl_HALO_Phase1_uid854_Phase2_uid1093/Sample_PM338_Z1_2_Case_HALO.Sample_PM338_EBC2_1_Ctrl_HALO.SNV.MAF
- PM338_Z1_2_Case_HALO_Phase1_uid1092__PM338_EBC2_1_Ctrl_HALO_Phase1_uid854_Phase2_uid1093/Sample_PM338_Z1_2_Case_HALO.Sample_PM338_EBC2_1_Ctrl_HALO.Unknown.MAF
- PM338_Z1_2_Case_HALO_Phase1_uid1092__PM338_EBC2_1_Ctrl_HALO_Phase1_uid854_Phase2_uid1093/Sample_PM338_Z1_2_Case_HALO.Sample_PM338_EBC2_1_Ctrl_HALO.annot.segments.CancerGenes.txt
- PM338_Z1_2_Case_HALO_Phase1_uid1092__PM338_EBC2_1_Ctrl_HALO_Phase1_uid854_Phase2_uid1093/Sample_PM338_Z1_2_Case_HALO.Sample_PM338_EBC2_1_Ctrl_HALO.annot.segments.ClinicallyRelevant.txt
- PM338_Z1_2_Case_HALO_Phase1_uid1092__PM338_EBC2_1_Ctrl_HALO_Phase1_uid854_Phase2_uid1093/Sample_PM338_Z1_2_Case_HALO.Sample_PM338_EBC2_1_Ctrl_HALO.annot.segments.Unknown.txt
- PM338_Z1_2_Case_HALO_Phase1_uid1092__PM338_EBC2_1_Ctrl_HALO_Phase1_uid854_Phase2_uid1093/Sample_PM338_Z1_2_Case_HALO.Sample_PM338_EBC2_1_Ctrl_HALO.indels.MAF
- PM338_Z1_2_Case_HALO_Phase1_uid1092__PM338_EBC2_1_Ctrl_HALO_Phase1_uid854_Phase2_uid1093/Sample_PM338_Z1_2_Case_HALO.Sample_PM338_EBC2_1_Ctrl_HALO.manually.filtered.MAF
- PM338_Z1_2_Case_HALO_Phase1_uid1092__PM338_EBC2_1_Ctrl_HALO_Phase1_uid854_Phase2_uid1093/Sample_PM338_Z1_2_Case_HALO.Sample_PM338_EBC2_1_Ctrl_HALO_CNV.png
- PM338_Z1_2_Case_HALO_Phase1_uid1092/Sample_PM338_Z1_2_Case_HALO.CLIA.coverage.txt
- PM338_Z1_2_Case_HALO_Phase1_uid1092/Sample_PM338_Z1_2_Case_HALO.covcapt.txt
- PM338_Z1_2_Case_HALO_Phase1_uid1092/Sample_PM338_Z1_2_Case_HALO.relevantGenes.coverage.txt
- PM338_EBC2_1_Ctrl_HALO_Phase1_uid854/Sample_PM338_EBC2_1_Ctrl_HALO.CLIA.coverage.txt
- PM338_EBC2_1_Ctrl_HALO_Phase1_uid854/Sample_PM338_EBC2_1_Ctrl_HALO.covcapt.txt
- PM338_EBC2_1_Ctrl_HALO_Phase1_uid854/Sample_PM338_EBC2_1_Ctrl_HALO.relevantGenes.coverage.txt

The report and appendix were generated at 13:34:36 EDT - Apr 11, 2016; based on version 235e7f2-dirty of the results data.

The report was generated at 13:34:36 EDT - Apr 11, 2016; based on version v1.6-14-g34708f2 of software IPM-reportGenerator, on version 0.1-86-g13c3a8c of the IPM knowledge base and cancer genes census, and on version 235e7f2-dirty of the results.

Copyright© 2013 - 2016 Cornell University. All Rights Reserved.
